# A light-sensing system in the common ancestor of the fungi

**DOI:** 10.1016/j.cub.2022.05.034

**Published:** 2022-07-25

**Authors:** Luis Javier Galindo, David S. Milner, Suely Lopes Gomes, Thomas A. Richards

**Affiliations:** 1 Department of Zoology, University of Oxford, Oxford OX1 3SZ, UK; 2 Departamento de Bioquímica, Instituto de Química, Universidade de São Paulo, São Paulo 05508-000, Brazil

**Keywords:** eyespot, rhodopsin, chytrid, protist, fungi, guanylyl cyclase, cGMP signaling, phototaxis

## Abstract

Diverse light-sensing organs (i.e., eyes) have evolved across animals. Interestingly, several subcellular analogs have been found in eukaryotic microbes.^1^ All of these systems have a common “recipe”: a light occluding or refractory surface juxtaposed to a membrane-layer enriched in type I rhodopsins.^1^, ^2^, ^3^, ^4^ In the fungi, several lineages have been shown to detect light using a diversity of non-homologous photo-responsive proteins.^5^, ^6^, ^7^ However, these systems are not associated with an eyespot-like organelle with one exception found in the zoosporic fungus *Blastocladiella emersonii* (*Be*).^8^*Be* possesses both elements of this recipe: an eyespot composed of lipid-filled structures (often called the side-body complex [SBC]), co-localized with a membrane enriched with a gene-fusion protein composed of a type I (microbial) rhodopsin and guanylyl cyclase enzyme domain (CyclOp-fusion protein).^8^^,^^9^ Here, we identify homologous pathway components in four Chytridiomycota orders (Chytridiales, Synchytriales, Rhizophydiales, and Monoblepharidiales). To further explore the architecture of the fungal zoospore and its lipid organelles, we reviewed electron microscopy data (e.g., the works of Barr and Hartmann^10^ and Reichle and Fuller^11^) and performed fluorescence-microscopy imaging of four CyclOp-carrying zoosporic fungal species, showing the presence of a variety of candidate eyespot-cytoskeletal ultrastructure systems. We then assessed the presence of canonical photoreceptors across the fungi and inferred that the last common fungal ancestor was able to sense light across a range of wavelengths using a variety of systems, including blue-green-light detection. Our data imply, independently of how the fungal tree of life is rooted, that the apparatus for a CyclOp-organelle light perception system was an ancestral feature of the fungi.

## Highlights

• A wide diversity of flagellated fungi possess the CyclOp light response circuit
• The same fungi possess the subcellular equipment to build lipid-based eyespots
• The last common ancestor of fungi possessed the CyclOp eyespot system
• The ancestral fungus could see a rainbow of light wavelengths

## Graphical abstract

**Figure:**
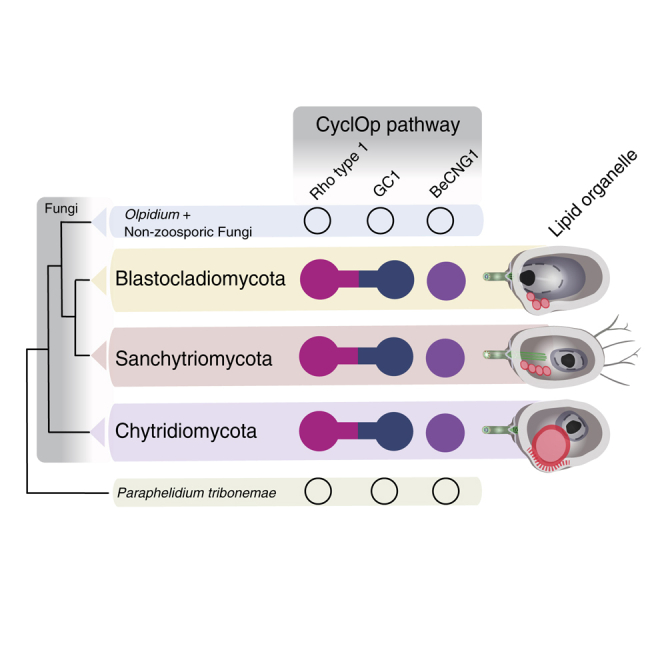

## Teaser

Galindo et al. demonstrate that a light response circuit, which controls swimming behavior, is present across diverse fungi. The circuit functions in coordination with a lipid-filled subcellular body. Divergent fungi possess similar cellular bodies. Phylogenies show that the ancestral fungus possessed this light-sensing system, along with other photoreceptors.

## Results and discussion

### Presence of the CyclOp-organelle components across the fungi

The CyclOp protein^9^ was first found to be in close spatial association with lipid-filled structures of the side-body complex (SBC) in *Blastocladiella emersonii* (*Be*),^8^ so this system is named here as the “CyclOp organelle.” The system was shown to control the phototaxis behavior of *Be* zoospores by amending intracellular cyclic guanosine monophosphate (cGMP) levels, which trigger the function of a cyclic nucleotide-gated ion channel (BeCNG1)^12^ and, therefore, regulates a green-light-sensing cascade, which results in flagellum beating.^8^

**Figure 1:**
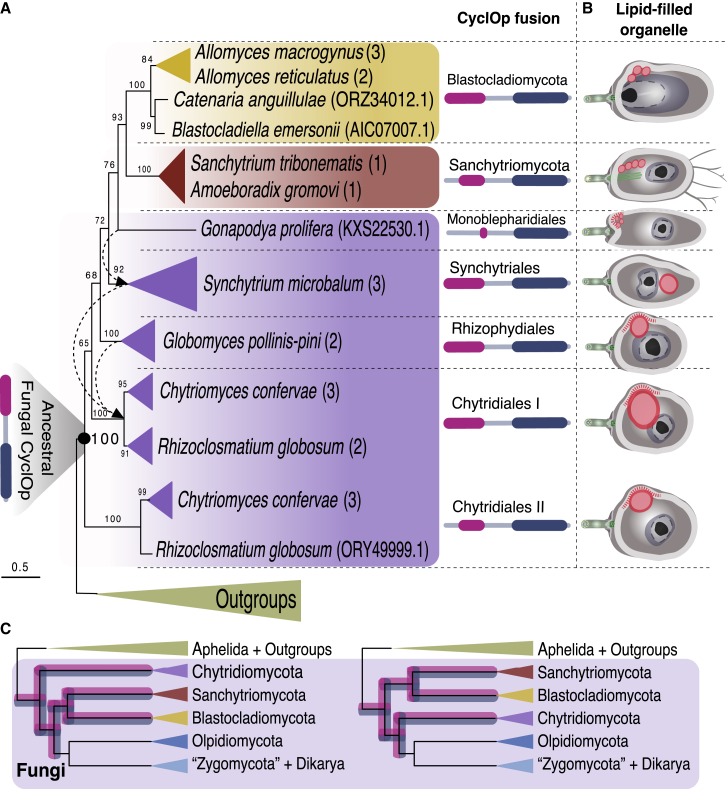
Evolution of the CyclOp system across the ancestral branches of the fungi (A) Schematic representation of a maximum likelihood phylogenetic tree of the CyclOp protein gene fusion. The tree was reconstructed using 84 sequences with 3,095 amino acid positions and was inferred with IQ-TREE under the LG + F + I + G4 model with 1,000 ultrafast bootstrap replicates to assess statistical support. Dashed-lined arrows indicate the position of a given clade in the trimmed analysis (with only the type I rhodopsin domain; see supplemental information). Bootstrap support values are indicated before the nodes. Numbers within brackets on the fungal clade labels indicate the number of sequences sampled. The overall protein domain structure of the CyclOp was identified using homology-based 3D structure modeling^8^ and is represented by rhodopsin (pink) and GC domains of the fusion (blue) (Figures S1–S3). (B) Depiction of candidate CyclOp-organelle systems within the zoospore ultrastructure based on the review of a wide range of microscopy data. Next to the tree, we show schematic depictions of the presence of lipid-filled organelles (red circles) in the zoospores of fungi that possess the CyclOp gene. These depictions are based on a comprehensive review of published microscopy data (Table S2). Dashed red lines represent the presence of a rumposome (Table S2). (C) Cladograms for the variant root hypotheses of the fungal tree and the evolution of the CyclOp system. Chytridiomycota-first (left) and Blastocladiomycota + Sanchytrids-first (right) hypotheses.^13^ Pink-blue highlight on the branches indicates the presence of the CyclOp system across the tree. See also Figures S1–S3 and Table S2.

The proteins needed for light sensing in a CyclOp organelle (CyclOp and BeCNG1 proteins) have been found in the genomes of Blastocladiomycota and Sanchytriomycota^8^^,^^13^ species. Using reciprocal BLAST and HMM protein sequence searches across the GenBank non-redundant databases^14^ and 45 publicly available proteomes from fungi and one aphelid species (a protist closely related to the fungi^15^) (Table S1), we identified all the elements that are known to function in the CyclOp pathway (CyclOp and BeCNG1 proteins) in two chytrid orders: Chytridiales (*Chytriomyces confervae* CBS 675.73 and *Rhizoclosmatium globosum* JEL800) and Synchytriales (*Synchytrium microbalum* JEL517) (Figures 1A, S1A–S1C, and S2). We found multiple distinct CygclOp proteins in *C. confervae* and *R. globosum*, indicating that duplicated gene forms are present in Chytridiales. Furthermore, in the *Globomyces pollinis-pini* (Rhizophydiales) genome, we detected the CyclOp fusion-protein-encoding gene but did not detect a BeCNG1 channel-encoding gene. The BeCNG1 channel protein was also missing from the *Homolaphlyctis polyrhiza* genome assembly, and, furthermore, we detected two unfused domains (rhodopsin type I and GC1) putatively encoded as separate genes 547 nucleotides apart on this genome assembly, demonstrating that this gene has undergone gene fission, a phenomenon shown to be common in fungi.^16^ Additionally, a member of one the earliest-diverging chytrid orders, Monoblepharidiales (*Gonapodya prolifera* JEL478), showed a partial CyclOp protein with a complete GC1 domain and a type I rhodopsin domain with a truncated N terminus (Figures 1A and S1A–S1C). The lack of a BeCNG1 protein and a truncated rhodopsin domain of the CyclOp protein indicates that this pathway is no longer functional or, alternatively, functions in a variant cGMP cascade in *Gonapodya*. In the Spizellomycetales species *Spizellomyces punctatus*, we found only the channel protein BeCNG1. We did not find the CyclOp system in the zoosporic aphelid *Paraphelidium tribonemae*, any of the Dikarya/Zygomycota fungi sampled, the frog pathogenic chytrid *Batrachochytrium dendrobatidis*, or in the Olpidiomycota *Olpidium bornovanus*. These observations suggest that the CyclOp system originated in the ancestor of the fungi, has been lost or reduced in some chytrids, and was lost prior to the large-scale radiation of Olpidiomycota, Zygomycota, and Dikarya fungi that led to the evolution of the “terrestrial” fungal clade (Figure 1C).

Phylogenetic analysis demonstrated the monophyletic relationship of both the CyclOp and the BeCNG1 protein sequences from the Chytridiomycota, Blastocladiomycota, and Sanchytriomycota species with strong statistical support (trimmed and/or complete alignment analysis; Figures 1A, S1A–S1C, and S2). The taxonomic distribution of these homologous proteins and their phylogenetic placement support the idea of an ancestral CyclOp-mediated light-sensing pathway involving an eyespot-organelle in chytrids, blastoclads, and sanchytrids, and therefore, this pathway was present in the common ancestor of all the fungi (Figures 1A and 1C).

SBCs and other prominent lipid organelles have been recognized as subcellular systems in a diversity of zoosporic fungi (known as the microbody-lipid globule complex [MLC] in Chytridiomycota species and the SBC in Blastocladiomycota)^17^ and are composed of microbodies, mitochondria, and several lipid globules enclosed by a membrane,^18^ are associated with ribosomes,^10^^,^^17^ and are usually located along one side of the nucleus. Homology of the SBC and MLC is not proven.^17^ Yet our review of microscopy data identifies similar structures in several representatives of zoosporic fungal phyla^19^ (Table S2). The function of this organelle has been linked to the provision of power for zoospore motility by continuous oxidation of lipids,^19^ but we argue that these cell structures are also used for the formation of an eyespot-like structure. This idea is further supported by the fact that all these species possess different variations of a prominent lipid organelle, arranged in a similar fashion as the CyclOp organelle of *Be* (Figure 1B).^17^ Indeed, ultrastructural studies of *Chytriomyces confervae* have already led to the suggestion that the system resembles an “eyespot”^10^ in addition to an energy storage and provision organelle. Therefore, we propose that the lipid globules could act to refract or obscure the light from one side of the cell, allowing such systems to perceive the direction of a light source, implying that the function of the SBC and MLC organelle systems is linked to the photo-response system. As discussed above, all these taxa also possess homologs of the CyclOp proteins shown to be critical for SBC-orchestrated phototaxis in *Be*.

### Structural confocal fluorescence imaging of fungal zoospores

**Figure 2:**
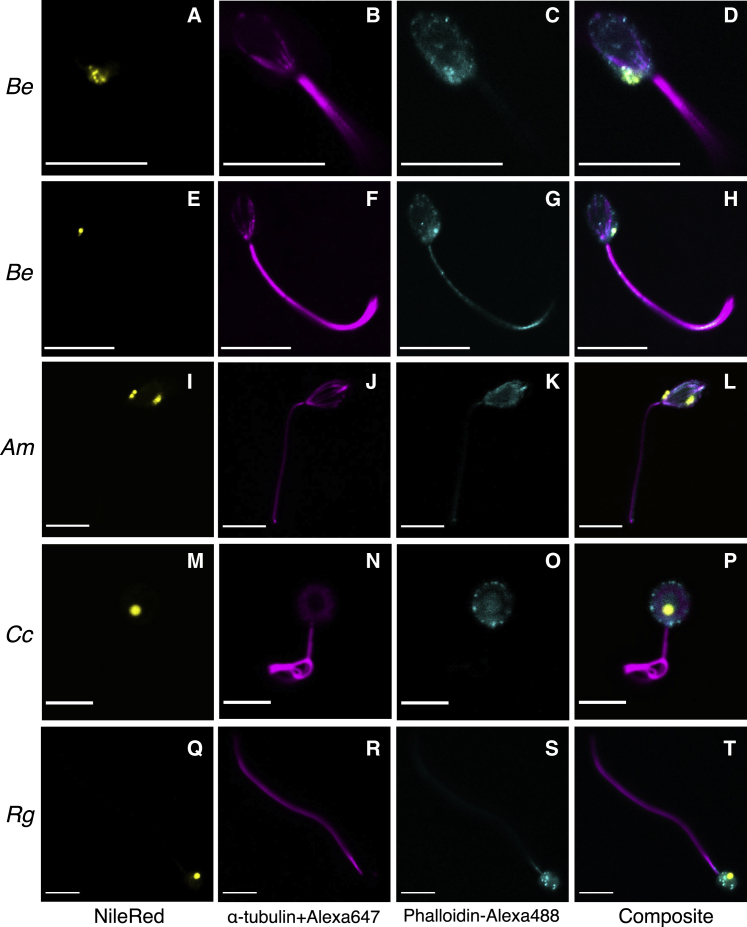
Confocal microscopic images of fluorescently stained fungal zoospores Rows are organized according to the fungal species to which the zoospores correspond: (A–H) *Blastocladiella emersonii* ATCC 22665 (*Be*), (I–L) *Allomyces macrogynus* Australia_3 (*Am*), (M–P) *Chytriomyces confervae* CBS 675.73 (*Cc*), and (Q–T) *Rhizoclosmatium globosum* JEL800 (*Rg*). Columns are ordered according to the stains used: (A–Q) Nile red staining for lipid droplets, (B–R) α-tubulin DM1A + Alexa Fluor 647 fluorescence staining for microtubules, (C–S) Alexa Fluor 488 Phalloidin for actin, and (D–T) composite image of all fluorescence channels. Scale bars, 10 μm (A–L) and 5 μm (M–T). Two rows are shown for *Be*; the first row is shown to identify the details of the cell body, and the second row shows the extent of the flagellum and the presence of Phalloidin stain (actin) within the flagellum. Supplemental information in FigShare shows a diversity of replicate zoospores imaged using this same approach.

To gain further insights into the intracellular organization of fungal zoospores and to understand how the cytoskeleton is arranged relative to the lipid organelle, we conducted confocal microscopy with antibodies/fluorescent dyes that preferentially stain cytoskeleton elements or lipid droplets. We conducted microscopy imaging on zoospores of four species of zoosporic fungi in which the presence of all components of the CyclOp optogenetic circuit was confirmed. Two Blastocladiomycota cultures (*Be* ATCC 22665 and *Allomyces macrogynus* Australia_3) and two Chytridiomycota cultures (*Chytriomyces confervae* CBS 675.73 and *Rhizoclosmatium globosum* JEL800) were imaged. We note it has been reported that *A. macrogynus* zoospores do not show phototaxis behavior unlike other *Allomyces* species.^20^,^9^,^21^ To stain the lipid components of zoospores, we used Nile red, and to stain the main components of the cytoskeleton, we used α-tubulin DM1A + Alexa Fluor 647 to stain tubulin and Alexa Fluor 488 Phalloidin to stain actin (Figure 2), both of which have previously been used to effectively study zoospore cytoskeletal systems in *B. dendrobatidis* and *R. globosum*.^22^^,^^23^

Our micrographs of *Be* (Figures 2A–2H; supplemental information in FigShare) show a large SBC located at the base of the flagellum and between the microtubular cytoskeleton of the zoospore and its cellular membrane. The SBC is composed of multiple lipid particles in close association (Figures 2A–2D). In *Allomyces*, the SBC is also located at the base of the flagellum and between the microtubule cytoskeleton and the cellular membrane but is composed of a lower number of lipid bodies (Figures 2I–2L; supplemental information in FigShare). *Allomyces* also demonstrates an agglomeration of multiple small apical lipid bodies in the anterior portion of the zoospores, located separately to the SBC.^24^ This arrangement of lipid particles imbedded within the SBC matrix in *Blastocladiella* and in *Allomyces* has previously been reported.^11^^,^^24^^,^^25^ However, the relative position of the SBC in relation to the cytoskeleton has not been well characterized. The cytoskeleton of both Blastocladiomycota species demonstrates a complex array of microtubules within the cell body and extending toward the flagellum (Figures 2B, 2F, and 2J). This array of microtubules has previously been observed as a Blastocladiomycota defining trait.^11^^,^^24^^,^^25^ These microtubules run in nine sets of triplets (in the case of *Allomyces*) from the kinetosome to the apical portion of the cell body enclosing the nucleus and the nuclear cap, with the mitochondria and the SCB on the opposite side of the cell. Actin is also present in both Blastocladiomycota species, creating a non-structured network all over the zoospore cell body (Figures 2C, 2G, and 2K), including the flagellum, which is discussed further below.

Our images for the chytrids *C. confervae* (Figures 2M–2P; supplemental information in FigShare) and *R. globosum* (Figures 2Q–2T; supplemental information in FigShare) show similar cytoplasmic organization, consistent with their close taxonomic relationships (order Chytridiales). Both species show one large and prominent lipid body, which corresponds to the MLC located toward the lower-middle region of the cell, and adjacent to the zoospore cell surface, where we know, from previous transmission electron microscopy (TEM) studies, that the enigmatic structure (unique to Chytridiomycota) called the rumposome is present (Figure 1B, dashed red lines).^10^^,^^17^^,^^26^ In these two chytrid species, microtubules are present only in the flagellum of the cells; however, previous TEM studies show that we can expect small microtubular bundles arising from the kinetosome and linking up directly to the rumposome. Higher-resolution imaging approaches would be necessary to observe these unique features that are potentially involved in phototaxis function.^10^ In contrast to what we observed for the Blastocladiomycota zoospores, actin is the main cytoskeletal protein in the cell body of Chytridiomycota studied here and organizes itself into actin patches, which were clearly visible in almost all zoospores observed from both species (Figures 2O and 2S; supplemental information in FigShare). These actin patches have previously been observed in chytrids,^22^^,^^23^ and they may correspond to what some authors refer to as fibrous areas identified from TEM analysis.^10^ In the chytrid *B.dendrobatidis*, these actin patches were observed in a minority of the cells, and it has been argued that these are involved in endocytosis in sporangia. Thus, these structures can be interpreted as a signal that zoospores may have initiated their transition to the sporangial growth stage.^23^ Some actin signal was also detected in the flagellum of a subset of the *R. globosum*, *B. emersonii*, and *A. macrogynus* zoospores (Figure 2; supplemental information in FigShare), a phenomenon previously observed in other eukaryotes.^27^, ^28^, ^29^

### Presence of other photoreceptor proteins across the fungi

**Figure 3:**
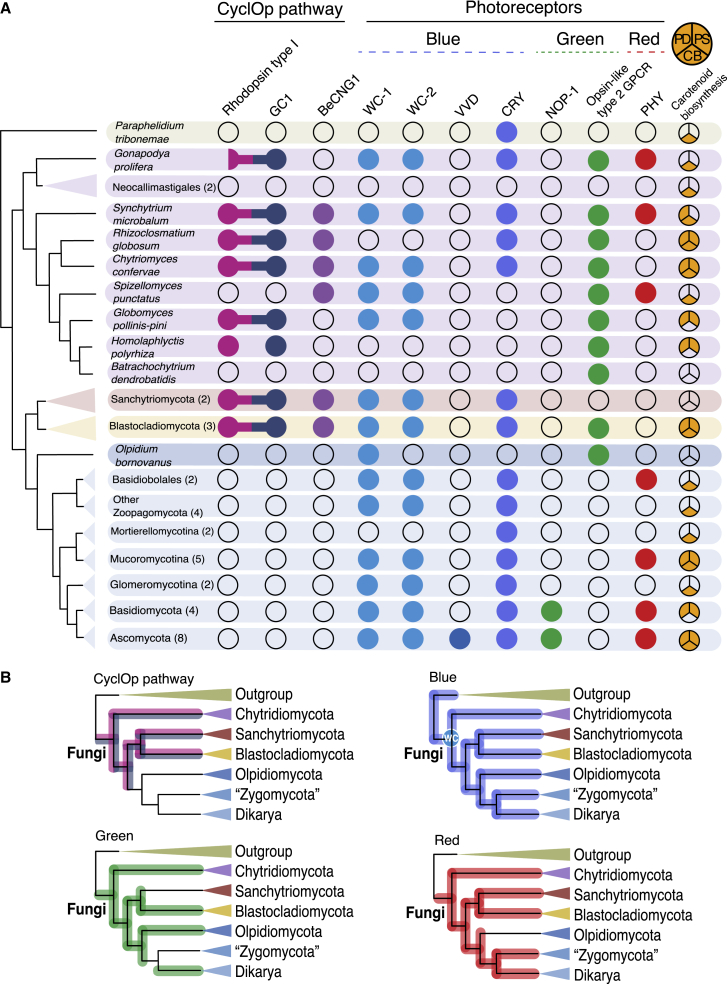
CyclOp pathway and photoreceptors across the fungal tree of life (A) Coulson plot of the presence/absence of the CyclOp pathway and other photoreceptor systems across the fungi. Colored dots indicate the presence of homologs of a given protein of the CyclOp pathway or specific photoreceptors. The type I rhodopsin and the GC1 domain of the CyclOp fusion are shown in pink and blue, respectively, and the BeCNG1 channel protein in purple. Protein domains that are encoded by a gene fusion are indicated by a connection between the dots, and an incomplete protein domain is represented by a half dot. Dots for photoreceptors are indicated in blue for blue light (WC-1 and its partner WC-2, VVD, and the cryptochrome CRY), green for green light (the opsin NOP-1 and the opsin-like type II GPCR), and red for red light (the phytochromes PHY-1 and PHY-2). The orange pie charts represent the presence/absence of the three enzymes involved in carotenoid biosynthesis: phytoene dehydrogenase (PD), phytoene synthase (PS), and carotenoid oxygenase (CB). Numbers between brackets on the fungal clades indicate the number of species proteomes analyzed. Table S3 (also available in FigShare) contains the data used to construct the Coulson plot. (B) Cladograms indicating the evolutionary path of the diversification of the photoreceptors of fungi. The CyclOp pathway (highlighted in pink and blue) and photoreceptors for green (highlighted in green), blue (highlighted in blue), and red (highlighted in red) are shown. WC, white-collar complex. See also Table S3.

A range of photoreceptive proteins have been identified across the fungi with a diversity of different mechanisms of action and a variety of structures and light sensitivities.^5^^,^^30^ To compare and contrast the evolution of the CyclOp system with other fungal photosystems, we performed protein searches of the previously mentioned 45 publicly available fungal/aphelid predicted proteomes (Table S1) in order to evaluate the presence of a diversity of candidate photoreceptors. These data allowed us to identify a picture of the evolution of these canonical photoreceptor systems across the fungi. This analysis includes searches for the following: (1) the conserved fungal photoreceptor proteins for blue light, the white-collar complex (WCC; White-Collar protein 1 [WC-1], White-Collar protein 2 [WC-2], and Vivid [VVD]); (2) the cryptochrome (CRY);^5^^,^^30^ (3) the green-light sensitive type I and II rhodopsins (the opsin NOP-1 and the type II opsin-like GPCR);^31^, ^32^, ^33^ and (4) the red-light-responsive phytochromes PHY-1 and PHY-2^5^^,^^34^^,^^35^ (Figure 3; Table S3, which is also available at FigShare: https://doi.org/10.6084/m9.FigShare.19182086.v4).

Our results show that blue-light sensing complex and variant photoreceptors can be traced back to the ancestor of the fungi, as they are present in all major fungal groups from zoosporic to dikaryan species. In the case of the WCC, WC-1 and WC-2 are found to be co-present in representatives of every major fungal group, consistent with the co-function of this complex, which can also be traced back to the last common ancestor of the fungi. However, the white-collar proteins are not present in the aphelid *Paraphelidium tribonemae* proteome. The VVD protein, which interacts with the WCC,^5^, ^30^ evolved in the ancestor of Ascomycota. By contrast, the blue receptor cryptochrome CRY protein is found throughout the fungi and is present in the aphelid *P. tribonemae*, so it must have evolved prior to the rise of the last common ancestor of the fungi (Figure 3B).

Aside from the rhodopsin present in the CyclOp-fusion protein, other distinct/unfused single-domain rhodopsin-protein-encoding genes have been shown to be present in fungal taxa,^7^ with the first published demonstration of the participation of a rhodopsin protein in phototaxis in the Blastocladiomycota *Allomyces reticulatus*.^20^ Opsins can be generally classified into two types, which share little sequence similarity: type I are typically found in prokaryotes and type II are primarily found in the Metazoa.^31^^,^^36^ Our results show that green-light sensing in fungi through the type I NOP-1 opsin has a limited distribution but can be traced back to the origin of dikaryan fungi (Figure 3). However, a type II rhodopsin was also found using structural comparisons in the zoosporic fungi *Spizellomyces punctatus* and later identified in other zoosporic relatives, e.g., *Allomyces macrogynus*, *Batrachochytrium dendrobatidis*, and *Homolaphlyctis polyrhiza*.^31^ We searched for this type II rhodopsin-like GPCR gene and found it in a range of zoosporic fungi but not in the sanchytrids and the Neocallimastigales (Figure 3). Thus, it seems that the type II rhodopsin was present in the fungal ancestor and was then lost in multiple taxa (e.g., Zoopagomycota, Mucoromycota, and Dikarya). The type I NOP-1 opsin was then acquired by dikaryan fungi, fulfilling green-light-sensing functions.^32^^,^^33^^,^^37^ The early evolution of blue- and green-light receptors in the fungi fits with its essential role in the regulation of circadian clocks and the predominance of blue-green-light wavelengths, which penetrate the aquatic depths,^38^, ^39^, ^40^ where the fungi first diversified.^41^^,^^42^

Lastly, red-light sensing in fungi is performed by the phytochromes PHY-1 and PHY-2.^5^^,^^34^^,^^35^^,^^43^ Our results show that red-light sensing can also be traced back to the dawn of the fungi since the genes encoding these proteins are present across the major fungal clades. However, it seems that the phytochromes have been lost several times within the fungi, including in Sanchytriomycota, Blastocladiomycota, Moretierellomycotina, and Glomeromycotina (Figure 3). It is interesting to highlight the absence of photoreceptor-encoding genes (including CyclOp) in Neocallimastigales, which likely reflects adaptation to a lifestyle within the dark digestive tracts of mammalian herbivores,^44^^,^^45^ whereas the loss of the CyclOp system in *Spizellomyces* may reflect their adaptation to soil environments.^46^^,^^47^

Rhodopsin function depends on the presence of a retinal chromophore co-factor.^8^^,^^48^ We searched the sampled genomes for the carotenoid (β-carotene) biosynthesis pathway enzyme genes necessary for the production of retinal. We found all three enzyme-encoding genes involved in this pathway (bifunctional lycopene cyclase/phytoene synthase, phytoene dehydrogenase, and carotenoid oxygenase)^8^ in the proteomes of the two Chytridiales species—all but the bifunctional lycopene cyclase/phytoene synthase in the proteome of *S. microbalum* and all but the carotenoid oxygenase in Rhizophydiales (except *Batrachochytrium*). In the case of Neocallimastigales, Spizellomycetales, and *G. prolifera*, we could find only one of the three enzymes (carotenoid oxygenase) (Figure 3; Table S3).

### Conclusions

Our results show a wide taxonomic distribution of the CyclOp system across zoosporic fungi. These data demonstrate that the ancestor of the fungi possessed the key elements of the CyclOp system, independent of the fungal root hypothesis favored (Figure 1C), and therefore may have been able to sense light through this pathway. The exploration of the intracellular organization of fungal zoospores using fluorescence microscopy demonstrates that a diversity of fungi produce zoospores containing lipid organelles, which are also likely to be associated with eyespot function. This result is again consistent with the idea that the last common ancestor of the fungi possessed the cellular equipment to build the cytological platform to support the function of the CyclOp optogenetic circuit. These results build upon a body of work demonstrating that early fungal forms sensed and responded to light in numerous different ways. Indeed, our data indicate that the ancestor of the fungi may have been able to sense blue, green, and red light, due to the presence of conserved canonical fungal photoreceptors. Such a result is also consistent with the hypothesis that early fungal forms were co-habiting and trophically interacting with photosynthetic forms since light sensing would allow early fungi to navigate toward environments inhabited by photosynthetic organisms.^19^^,^^41^

## STAR★Methods

### Key resources table

**Table:** 

REAGENT or RESOURCE	SOURCE	IDENTIFIER
**Antibodies**		

α-tubulin DM1A	Sigma-Aldrich	Cat#T6199; RRID: AB_477583
Alexa Fluor 647 Goat anti-mouse IgG1 antibody	Thermo Fisher	Cat#A-21240; RRID: AB_2535809
Alexa Fluor 488 Phalloidin	Invitrogen	Cat#A12379; RRID: AB_2759222

**Stains**		

Nile Red	Thermo Fisher	Cat#N1142

**Reagents**		

Citifluor AF2 Antifadent Mountant Solution	Electron Microscopy Sciences	AF2

**Deposited data**		

CyclOp pathway and photoreceptors in fungi, alignment datasets and trees for the CyclOp and BeCNG1 proteins, trees and aligned datasets used for phylogenetic reconstruction of the CyclOp pathway in the Fungi, table of photoreceptors and retinal synthesis genes.	This study	https://doi.org/10.6084/m9.figshare.19182086.v4

**Experimental models: Organisms/strains**		

*Blastocladiella emersonii* ATCC 22665	ATCC	ATCC 22665
*Chytridium confervae* CBS 675.73	Westerdijk Fungal Biodiversity Institute	CBS 675.73
*Rhizoclosmatium globosum* JEL800	CZEUM	JEL800
*Allomyces macrogynus* Australia_3	CZEUM	Australia_3

**Software and algorithms**		

Fiji 2.3.1	Schindelin et al. ^49^	https://fiji.sc/; RRID: SCR_002285
Black Zen software	ZEN Digital Imaging for Light Microscopy	https://www.zeiss.com/microscopy/int/products/microscope-software/zen.html; RRID: SCR_018163
BLAST	Altschul et al. ^50^	https://blast.ncbi.nlm.nih.gov/Blast.cgi; RRID: SCR_004870
HMMER v3.3.2	Finn et al. ^51^	https://www.ebi.ac.uk/Tools/hmmer/search/hmmscan; RRID: SCR_005305
MAFFT version 7	Katoh et al. ^52^	https://mafft.cbrc.jp/alignment/server/; RRID: SCR_011811
TrimAl v1.2	Capella-Gutiérrez et al.^53^	http://trimal.cgenomics.org/; RRID: SCR_017334
IQ-TREE version 1.6.12	Nguyen et al. ^54^	http://www.iqtree.org/; RRID: SCR_017254
FigTree v1.4.3	Rambaut ^55^	http://tree.bio.ed.ac.uk/software/figtree/; RRID: SCR_008515

### Resource availability

#### Lead contact

Further information and requests for resources and reagents should be directed to and will be fulfilled by the Lead Contact, Thomas A. Richards (thomas.richards@zoo.ox.ac.uk).

#### Materials availability

This study did not generate new unique reagents.

### Experimental model and subject details

Cultures of *Blastocladiella emersonii* ATCC 22665 (ATCC; American Type Culture Collection) and *Chytriomyces confervae* CBS 675.73 (Westerdijk Fungal Biodiversity Institute) were vegetatively grown in Nunc EasYFlask 25cm^2^ culture flasks (ThermoFisher) filled with 25 ml of PYG liquid media (0.13% w/v peptone, 0.13% w/v yeast extract, 0.3% w/v glucose). *Rhizoclosmatium globosum* JEL800 (CZEUM; Collection of Zoosporic Eufungi at University of Michigan) vegetative cells were grown on PYG agar plates (0.13% w/v peptone, 0.13% w/v yeast extract, 0.3% w/v glucose, and 1.5% w/v agar). *Allomyces macrogynus* Australia_3 (CZEUM) was grown in 25cm^2^ culture flasks filled with 25 ml of Emerson YpSs/4 liquid media (0.1% w/v yeast extract, 0.375% w/v soluble starch, 0.025% w/v dipotassium phosphate, 0.01% w/v magnesium sulfate). All fungal vegetative growth was performed at 21°C and transferred every two weeks by inoculating 25 μl of previous culture to a new flask/plate containing 25 ml of media.

To induce sporulation of *Blastocladiella emersonii* ATCC 22665 and *Chytriomyces confervae* CBS 675.73, 400 ml of liquid PYG was inoculated with 20 ml of vegetative cells in a 2.8 L Fernbach flask, and incubated, with 150 rpm agitation, for 24 h at 21°C. Zoospores were then separated from sporangia using a 20 μm pluriStrainer (pluriSelect) in 50 ml falcon tubes.

*Rhizoclosmatium globosum* JEL800 zoospore obtention was performed by growing this strain on PYG agar plates for ∼4 days. Plates were then flooded with 7 ml of distilled water at 21°C for 15 min followed by removal of zoospore-containing water to a 15 ml falcon tube (this process was repeated twice per plate).

To induce sporulation in *Allomyces macrogynus* Australia_3, vegetative growth was performed for ∼5 days in 25 ml of liquid YpSs/4 media. Sporangia were then separated from the culture using sterile tweezers, washed with Volvic Natural Mineral Water, and placed in 20 ml of Volvic water in a 25cm^2^ culture flask at 21°C overnight. Zoospores were separated from sporangia using a 20 μm pluriStrainer (pluriSelect) in 50 ml falcon tubes.

### Method details

#### CyclOp pathway gene database searches and phylogenetic analyses

To study the presence or absence of the CyclOp (BeGC1) fusion proteins and the BeCNG1 light-sensing proteins, BLAST^50^ and HMM^51^ searches were performed against the complete GenBank non-redundant databases^56^ (Last searched on January 2022) using the proteins BeGC1 (GenBank: AIC07007.1) and BeCNG1 (GenBank: AIC07008.1) as queries. Additionally, local BLAST searches were performed on 45 publicly available proteomes from fungal and one aphelid species (Table S1).

The phylogenetic datasets were based on the protein datasets of Avelar et al.^8^ in which the authors BLAST searched both the guanylyl-cyclase GC1 and the rhodopsin domains of the CyclOp fusion protein and the BeCNG1 channel protein of *B. emersonii* against a database of more than 900 genomes from eukaryotic and prokaryotic species from across the tree of life (see Table S1 of Avelar et al.^8^). MAFFT^52^ was used to incorporate identical sequences from the analysed proteomes into a multiple sequence alignment creating a dataset for the complete CyclOp gene fusion, two trimmed datasets for each domain (type 1 rhodopsin and GC1) of the fusion protein and one for the BeCNG1 protein channel^8^ (see Figures S1A–S1C, S2, and S3, with alignments available from FigShare). After trimming with TrimAl^53^ with the automated1 option Maximum Likelihood (ML), trees were inferred using IQ-TREE^54^ under the LG+F+I+G4 model for the complete CyclOp fusion dataset (Figure S1A) and for the type I rhodopsin domain dataset (Figure S1B), LG+R5+C60 model for the GC1 guanylyl cyclase domain dataset (Figure S1C) and LG+C60+I+G4 for the dataset of the BeCNG1 channel protein (Figure S2). All alignments, datasets and trees can be found at Figshare: https://doi.org/10.6084/m9.FigShare.19182086.v4.

#### Fungal photoreceptors searches across proteomes

To test the presence of fungal photoreceptors across the fungal tree, localBLAST and HMM searches were performed on 45 publicly available proteomes from fungal and (one) aphelid species (Table S1). The queries for blue-light photoreceptor proteins were White Collar Complex proteins (WCC) WC-1, WC-2, Vivid (VVD) (GenBank: ESA41977.1, CAA70336.1, AAK08514.1) and the cryptochrome (CRY; GenBank: EAA36486.3);^5^^,^^30^ the green-light type I and II rhodopsin (the opsin NOP-1; GenBank: AAD45253.1 and the type 2 opsin-like GPCR; SPPG_00350T0L [see source reference]);^31^, ^32^, ^33^ and the red light phytochromes PHY-1 and PHY-2^5^^,^^34^^,^^35^ (AAZ57422.1 and AAZ57421.1). All these seed sequences were identified from *Neurospora crassa*, with the exception of the type 2 rhodopsin which came from *Spizellomyces punctatus*^5^^,^^31^ (Table S3).

To study the possible presence of a carotenoid synthesis pathway for retinal production, a combination of previous datasets for carotenoid biosynthesis and cleavage enzymes from one giant virus (ChoanoV1), two choanoflagellates and two haptophytes,^48^ together with the carotenoid biosynthesis enzymes found in *B. emersonii*^8^ was used. This dataset also included three enzymes for early sterol and carotenoid biosynthesis (isoprenoid biosynthesis steps).^48^ The protein sequences from this dataset were BLAST searched^50^ (BLASTp) against the proteomes of the 44 selected fungal species (Table S1). All results were confirmed by reciprocal BLASTp searches of the candidate sequences identified to the NCBI non-redundant protein sequence database and HMMscan searches^51^ (see Table S3).

#### Synthesis of ultrastructure, protein architecture and evolutionary data

Ultrastructural information about intracellular architecture and localization of the side-body complexes and lipid bodies used for the drawings in Figure 1B came from ultrastructural studies of zoosporic fungi^10^^,^^11^^,^^24^^,^^25^^,^^57^, ^58^, ^59^, ^60^, ^61^, ^62^ (Table S2). Representation of the CyclOp protein and its domains from Figure 1 are based on the CyclOp domain alignment architecture (Figure S3) and the domain hits obtained for each sequence with HMMR/BLAST web server tools.^51^^,^^56^

#### Fluorescence microscopy

Zoospores were collected from the culture medium by concentrating them by centrifugation at 1000 x g for 5 min followed by removal of the supernatant. In all cases the remaining pellet was fixed in 0.5 ml of 4% w/v paraformaldehyde in 1X PBS and transferred into a 15 ml Falcon tube for 15 min at room temperature. Cells were concentrated by centrifugation and resuspended in PBS for a first washing step. The cell pellet was then resuspended and permeabilized in 0.5 ml of PBS containing 0.1% v/v Triton X-100. A second washing step in 0.5 ml of PBS was performed and then the cells were blocked with 0.5 ml of 1% w/v BSA in PBS and incubated for 45 min at room temperature, followed by the addition of the primary antibody α-tubulin DM1A (Sigma-Aldrich, Cat# T6199, RRID: AB_477583) at a concentration of 1:500 v/v for 120 min at room temperature. After a third washing step of the primary antibody with 1X PBS, the secondary antibody Alexa Fluor 647 Goat anti-mouse IgG1 antibody was added (Thermo Fisher Scientific, Cat#A-21240, RRID: AB_2535809) to the PBS-resuspended fixed zoospore solution for 60 min at room temperature. Alexa Fluor 488 Phalloidin (Invitrogen, Cat# A12379, RRID: AB_2759222) and Nile Red (Thermo Fisher Scientific, Cat# N1142) were also added at a 1:500 v/v concentration for 60 min at room temperature. After two washing steps with 1X PBS, the final cell pellets were resuspended in 100 μl of 20% v/v Citifluor AF2 Antifadent Mountant Solution and 7 μl was placed on a slide and covered with a coverslip, which was then sealed with transparent nail polish on the edges to avoid evaporation. Cells were imaged on a Zeiss LSM-780 inverted high-resolution laser scanning confocal microscope with a Ph3 ×100 oil objective. Exposures were kept constant during experiments, and images were collected using BLACK ZEN Software (ZEN Digital Imaging for Light Microscopy), and analyzed/formatted with Fiji.^49^

### Quantification and statistical analysis

Best-fitting phylogenetic models were selected with the IQ-TREE^54^ TESTNEW algorithm as per BIC for the all datasets, obtaining the following best-fitting models: LG+F+I+G4 for the complete CyclOp fusion dataset (Figure S1A) and for the type I rhodopsin domain dataset (Figure S1B), LG+R5+C60 model for the GC1 guanylyl cyclase domain dataset (Figure S1C) and LG+C60+I+G4 for the dataset of the BeCNG1 channel protein (Figure S2). Statistical support was evaluated using 1000 ultrafast bootstrap replicates and 1000 replicates of the SH-like approximate likelihood ratio test and the resulting trees were visualized with FigTree.^55^

## Data Availability

All data are available in the figures, tables, and data files associated with this manuscript. This study did not result in any unique code. Any additional information required to reanalyze the data reported in this work paper is available from the Lead Contact upon request

## Supplemental information

Document S1. Figures S1–S3 and Tables S1–S3

Document S2. Article plus supplemental information

## References

[1] Richards T.A., Gomes S.L. (2015). Protistology: how to build a microbial eye. Nature.

[2] Schmidt M., Gessner G., Luff M., Heiland I., Wagner V., Kaminski M., Geimer S., Eitzinger N., Reissenweber T., Voytsekh O. (2006). Proteomic analysis of the eyespot of *Chlamydomonas reinhardtii* provides novel insights into its components and tactic movements. Plant Cell.

[3] Gavelis G.S., Hayakawa S., White R.A., Gojobori T., Suttle C.A., Keeling P.J., Leander B.S. (2015). Eye-like ocelloids are built from different endosymbiotically acquired components. Nature.

[4] Sineshchekov O.A., Govorunova E.G., Jung K.H., Zauner S., Maier U.G., Spudich J.L. (2005). Rhodopsin-mediated photoreception in cryptophyte flagellates. Biophys. J..

[5] Corrochano L.M. (2019). Light in the fungal world: from photoreception to gene transcription and beyond. Annu. Rev. Genet..

[6] Yu Z., Fischer R. (2019). Light sensing and responses in fungi. Nat. Rev. Microbiol..

[7] Brown L.S. (2004). Fungal rhodopsins and opsin-related proteins: eukaryotic homologues of bacteriorhodopsin with unknown functions. Photochem. Photobiol. Sci..

[8] Avelar G.M., Schumacher R.I., Zaini P.A., Leonard G., Richards T.A., Gomes S.L. (2014). A rhodopsin-guanylyl cyclase gene fusion functions in visual perception in a fungus. Curr. Biol..

[9] Gao S., Nagpal J., Schneider M.W., Kozjak-Pavlovic V., Nagel G., Gottschalk A. (2015). Optogenetic manipulation of cGMP in cells and animals by the tightly light-regulated guanylyl-cyclase opsin CyclOp. Nat. Commun..

[10] Barr D.J.S., Hartmann V.E. (1976). Zoospore ultrastructure of three *Chytridium* species and *Rhizoclosmatium globosum*. Can. J. Bot..

[11] Reichle R.E., Fuller M.S. (1967). The fine structure of *Blastocladiella emersonii* zoospores. Am. J. Bot..

[12] Avelar G.M., Glaser T., Leonard G., Richards T.A., Ulrich H., Gomes S.L. (2015). A cyclic GMP-dependent K+ channel in the blastocladiomycete fungus *Blastocladiella emersonii*. Eukaryot. Cell.

[13] Galindo L.J., López-García P., Torruella G., Karpov S., Moreira D. (2021). Phylogenomics of a new fungal phylum reveals multiple waves of reductive evolution across Holomycota. Nat. Commun..

[14] Pruitt K.D., Tatusova T., Maglott D.R. (2007). NCBI reference sequences (RefSeq): a curated non-redundant sequence database of genomes, transcripts and proteins. Nucleic Acids Res..

[15] Torruella G., Grau-Bové X., Moreira D., Karpov S.A., Burns J.A., Sebé-Pedrós A., Völcker E., López-García P. (2018). Global transcriptome analysis of the aphelid *Paraphelidium tribonemae* supports the phagotrophic origin of fungi. Commun. Biol..

[16] Leonard G., Richards T.A. (2012). Genome-scale comparative analysis of gene fusions, gene fissions, and the fungal tree of life. Proc. Natl. Acad. Sci. USA.

[17] Powell M.J. (1978). Phylogenetic implications of the microbody-lipid globule complex in zoosporic fungi. Biosystems.

[18] James T.Y., Porter T.M., Martin W.W., McLaughlin D.J., Spatafora J.W. (2014). Systematics and Evolution.

[19] Powell M.J. (2017). Handbook of the Protists.

[20] Saranak J., Foster K.W. (1997). Rhodopsin guides fungal phototaxis. Nature.

[21] Swafford A.J.M., Oakley T.H. (2018). Multimodal sensorimotor system in unicellular zoospores of a fungus. J. Exp. Biol..

[22] Venard C.M., Vasudevan K.K., Stearns T. (2020). Cilium axoneme internalization and degradation in chytrid fungi. Cytoskeleton (Hoboken).

[23] Prostak S.M., Robinson K.A., Titus M.A., Fritz-Laylin L.K. (2021). The actin networks of chytrid fungi reveal evolutionary loss of cytoskeletal complexity in the fungal kingdom. Curr. Biol..

[24] Fuller M.S., Olson L.W. (1971). The zoospore of *Allomyces*. Microbiology.

[25] Cantino E.C., Truesdell L.C. (1970). Organization and fine structure of the side body and its lipid sac in the zoospore of *Blastocladiella emersonii*. Mycologia.

[26] Powell M.J. (1983). Localization of antimonate-mediated precipitates of cations in zoospores of *Chytriomyces hyalinus*. Exp. Mycol..

[27] Watanabe Y., Hayashi M., Yagi T., Kamiya R. (2004). Turnover of actin in *Chlamydomonas* flagella detected by fluorescence recovery after photobleaching (FRAP). Cell Struct. Funct..

[28] Kiesel P., Alvarez Viar G., Tsoy N., Maraspini R., Gorilak P., Varga V., Honigmann A., Pigino G. (2020). The molecular structure of mammalian primary cilia revealed by cryo-electron tomography. Nat. Struct. Mol. Biol..

[29] Piperno G., Luck D.J. (1979). An actin-like protein is a component of axonemes from *Chlamydomonas* flagella. J. Biol. Chem..

[30] Idnurm A., Verma S., Corrochano L.M. (2010). A glimpse into the basis of vision in the kingdom *Mycota*. Fungal Genet. Biol..

[31] Ahrendt S.R., Medina E.M., Chang C.-E.A., Stajich J.E. (2017). Exploring the binding properties and structural stability of an opsin in the chytrid *Spizellomyces punctatus* using comparative and molecular modeling. PeerJ.

[32] Bieszke J.A., Spudich E.N., Scott K.L., Borkovich K.A., Spudich J.L. (1999). A eukaryotic protein, NOP-1, binds retinal to form an archaeal rhodopsin-like photochemically reactive pigment. Biochemistry.

[33] Wang Z., Wang J., Li N., Li J., Trail F., Dunlap J.C., Townsend J.P. (2018). Light sensing by opsins and fungal ecology: NOP-1 modulates entry into sexual reproduction in response to environmental cues. Mol. Ecol..

[34] Blumenstein A., Vienken K., Tasler R., Purschwitz J., Veith D., Frankenberg-Dinkel N., Fischer R. (2005). The *Aspergillus nidulans* phytochrome FphA represses sexual development in red light. Curr. Biol..

[35] Brandt S., von Stetten D., Günther M., Hildebrandt P., Frankenberg-Dinkel N. (2008). The fungal phytochrome FphA from *Aspergillus nidulans*. J. Biol. Chem..

[36] Shichida Y., Matsuyama T. (2009). Evolution of opsins and phototransduction. Philos. Trans. R. Soc. Lond. B Biol. Sci..

[37] Bieszke J.A., Braun E.L., Bean L.E., Kang S., Natvig D.O., Borkovich K.A. (1999). The nop-1 gene of *Neurospora crassa* encodes a seven transmembrane helix retinal-binding protein homologous to archaeal rhodopsins. Proc. Natl. Acad. Sci. USA.

[38] Williams D.L. (2016). Light and the evolution of vision. Eye (Lond).

[39] Montenegro-Montero A., Canessa P., Larrondo L.F. (2015). Around the fungal clock: recent advances in the molecular study of circadian clocks in *Neurospora* and other fungi. Adv. Genet..

[40] Liu Y., Bell-Pedersen D. (2006). Circadian rhythms in *Neurospora crassa* and other filamentous fungi. Eukaryot. Cell.

[41] Lutzoni F., Nowak M.D., Alfaro M.E., Reeb V., Miadlikowska J., Krug M., Arnold A.E., Lewis L.A., Swofford D.L., Hibbett D. (2018). Contemporaneous radiations of fungi and plants linked to symbiosis. Nat. Commun..

[42] Naranjo-Ortiz M.A., Gabaldón T. (2019). Fungal evolution: major ecological adaptations and evolutionary transitions. Biol. Rev. Camb. Philos. Soc..

[43] Wang Z., Li N., Li J., Dunlap J.C., Trail F., Townsend J.P. (2016). The fast-evolving phy-2 gene modulates sexual development in response to light in the model fungus *Neurospora crassa*. mBio.

[44] Powell M.J. (2017). Handbook of the Protists.

[45] Gruninger R.J., Puniya A.K., Callaghan T.M., Edwards J.E., Youssef N., Dagar S.S., Fliegerova K., Griffith G.W., Forster R., Tsang A. (2014). Anaerobic fungi (phylum *Neocallimastigomycota*): advances in understanding their taxonomy, life cycle, ecology, role and biotechnological potential. FEMS Microbiol. Ecol..

[46] Paulitz T.C., Menge J.A. (1984). Is *Spizellomyces punctatum* a parasite or saprophyte of vesicular-arbuscular mycorrhizal fungi?. Mycologia.

[47] Lozupone C.A., Klein D.A. (2002). Molecular and cultural assessment of chytrid and *Spizellomyces* populations in grassland soils. Mycologia.

[48] Needham D.M., Yoshizawa S., Hosaka T., Poirier C., Choi C.J., Hehenberger E., Irwin N.A.T., Wilken S., Yung C.M., Bachy C. (2019). A distinct lineage of giant viruses brings a rhodopsin photosystem to unicellular marine predators. Proc. Natl. Acad. Sci. USA.

[49] Schindelin J., Arganda-Carreras I., Frise E., Kaynig V., Longair M., Pietzsch T., Preibisch S., Rueden C., Saalfeld S., Schmid B. (2012). Fiji: an open-source platform for biological-image analysis. Nat. Methods.

[50] Altschul S.F., Gish W., Miller W., Myers E.W., Lipman D.J. (1990). Basic local alignment search tool. J. Mol. Biol..

[51] Finn R.D., Clements J., Eddy S.R. (2011). HMMER web server: interactive sequence similarity searching. Nucleic Acids Res..

[52] Katoh K., Misawa K., Kuma K.I., Miyata T. (2002). MAFFT: a novel method for rapid multiple sequence alignment based on fast Fourier transform. Nucleic Acids Res..

[53] Capella-Gutiérrez S., Silla-Martínez J.M., Gabaldón T. (2009). trimAl: a tool for automated alignment trimming in large-scale phylogenetic analyses. Bioinformatics.

[54] Nguyen L.T., Schmidt H.A., Von Haeseler A., Minh B.Q. (2015). IQ-TREE: a fast and effective stochastic algorithm for estimating maximum-likelihood phylogenies. Mol. Biol. Evol..

[55] Rambaut A. (2016). http://tree.bio.ed.ac.uk/software/figtree/.

[56] Clark K., Karsch-Mizrachi I., Lipman D.J., Ostell J., Sayers E.W. (2016). GenBank. Nucleic Acids Res..

[57] Mollicone M.R.N., Longcore J.E. (1999). Zoospore ultrastructure of *Gonapodya polymorpha*. Mycologia.

[58] Manier J.-F. (1977). Cycle, ultrastructure d’une *Catenaria* (Phycomycètes, Blastocladiales) parasite de Crustacés Cyclopoides. Ann. Parasitol. Hum. Comp..

[59] Karpov S.A., Vishnyakov A.E., Moreira D., López-García P. (2019). The ultrastructure of *Sanchytrium tribonematis* (Sanchytriaceae, Fungi *incertae sedis*) confirms its close relationship to *Amoeboradix*. J. Eukaryot. Microbiol..

[60] Karpov S.A., López-García P., Mamkaeva M.A., Klimov V.I., Vishnyakov A.E., Tcvetkova V.S., Moreira D. (2018). The chytrid-like parasites of algae *Amoeboradix gromovi* gen. et sp. nov. and *Sanchytrium tribonematis* belong to a new fungal lineage. Protist.

[61] Longcore J.E., Simmons D.R., Letcher P.M. (2016). *Synchytrium microbalum* sp. nov. is a saprobic species in a lineage of parasites. Fungal Biol..

[62] Letcher P.M., Vélez C.G., Barrantes M.E., Powell M.J., Churchill P.F., Wakefield W.S. (2008). Ultrastructural and molecular analyses of *Rhizophydiales* (*Chytridiomycota*) isolates from North America and Argentina. Mycol. Res..

