# Supplementary material for: A light-sensing system in the common ancestor of the fungi
Source: Curr Biol. 2022 Jul 25;32(14):3146–3153.e3. doi: 10.1016/j.cub.2022.05.034 (PMC9616733; doi:10.1016/j.cub.2022.05.034)
Supplement: Document S1. Figures S1–S3 and Tables S1–S3 [file mmc1.pdf]

**Current Biology, Volume *32***

## Supplemental Information

# A light-sensing system
# in the common ancestor of the fungi

**Luis Javier Galindo, David S. Milner, Suely Lopes Gomes, and Thomas A. Richards**

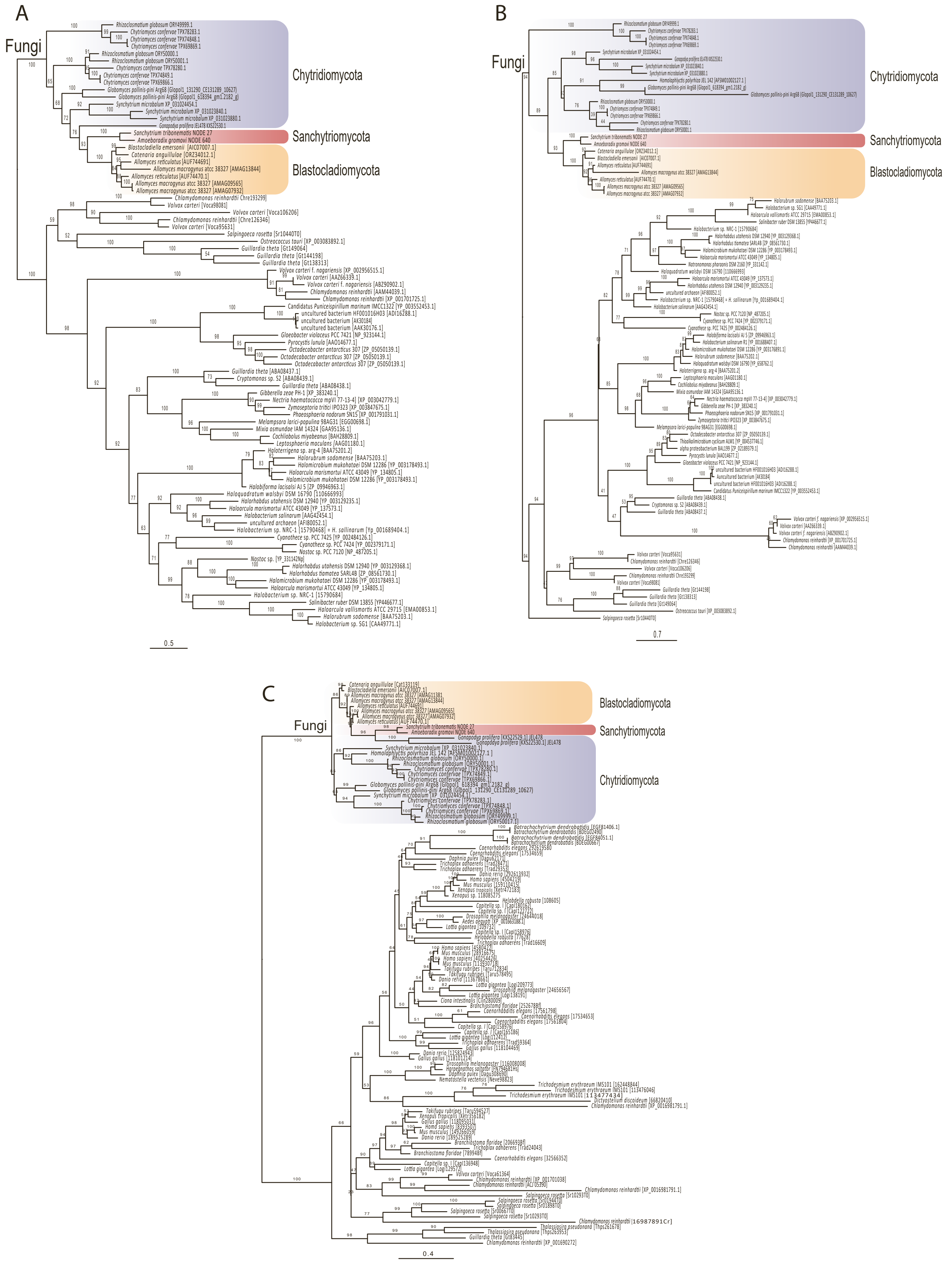
A
Fungi
Chytridiomycota
Sanchytriomycota
Blastocladiomycota
0.5
B
Fungi
Chytridiomycota
Sanchytriomycota
Blastocladiomycota
0.7
C
Fungi
Blastocladiomycota
Sanchytriomycota
Chytridiomycota
0.4

**Figure S1. Maximum likelihood tree based on the protein dataset of Avelar et al. (2014)[S1], related to Figure 1. (A)** Phylogenetic reconstruction of the CyclOp (BeGC1) protein-fusion, with 84 sequences and 3095 amino acidic positions, the tree was inferred using IQ-TREE under the LG+F+I+G4 model with 1000 ultrafast bootstrap as statistical support. **(B)** Phylogenetic reconstruction of the Type I bacterial rhodopsin of the CyclOp (BeGC1) protein-fusion, with 85 sequences and 127 amino acidic positions, the tree was inferred using IQ-TREE under the LG+F+I+G4 model with 1000 ultrafast bootstrap as statistical support. **(C)** Phylogenetic reconstruction of the GC1 guanylyl-cyclase domain of the CyclOp (BeGC1) protein-fusion, with 107 sequences and 157 amino acidic positions, the tree was inferred using IQ-TREE under the LG+R5+C60 model with 1000 ultrafast bootstrap as statistical support. These results underpin the summary data figure shown in Figure 1.

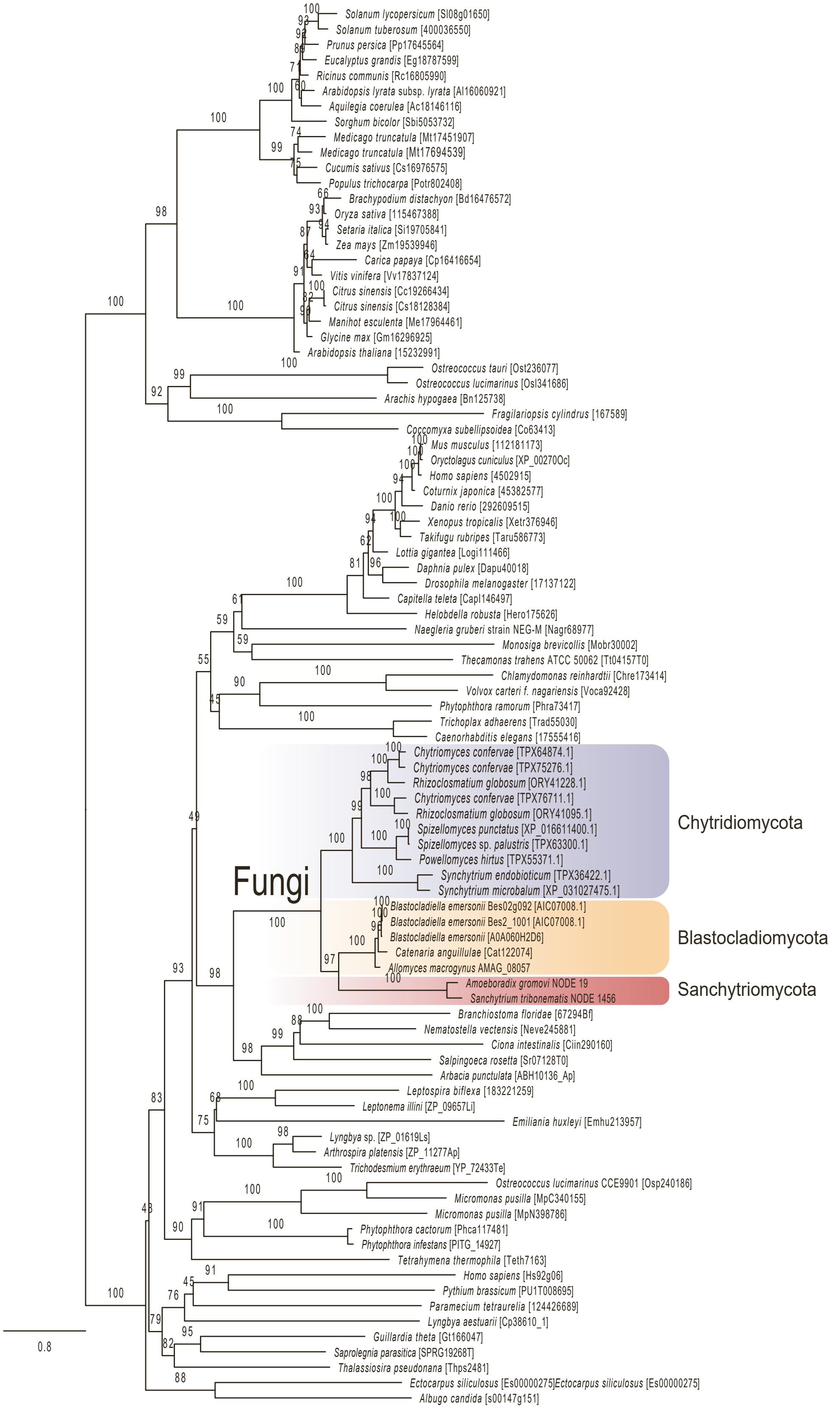


**Figure S2. Maximum likelihood tree based on the protein dataset of Avelar et al. (2014)[S1], related to Figure 1.** Phylogenetic reconstruction of the cyclic nucleotide gated channel BeCNG1, with 91 sequences and 307 amino acidic positions, the tree was inferred using IQ-TREE under the LG+C60+I+G4 model with 1000 ultrafast bootstrap as statistical support. These results underpin the summary data figure shown in Figure 1.

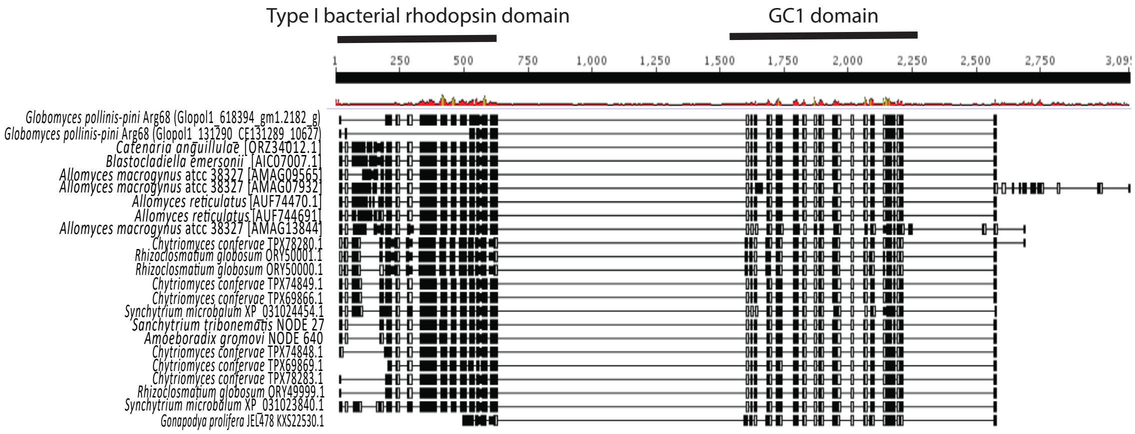


**Figure S3. Alignment of CyclOp sequences from Chytridiomycota, Sanchytriomycota and Blastocladiomycota based on the protein dataset of Avelar et al. (2014)[S1], related to Figure 1.** The alignment was made using MAFFT. These results underpin the summary data figure shown in Figure 1.

| Taxon | Database used | BioProject | BioSample | Sample source | Assembly/SRA/EST ID | Year | Reference publication |
|---|---|---|---|---|---|---|---|
| *Paraphelidium tribonemae* X-108 | NCBI | PRJNA402032 | SAMN07614855 / SAMN07614854 | ESE lab, CNRS, Paris-Saclay University | SRR6014119 / SRR6014120 | 2018 | Torruella et al. [S2] |
| *Gonapodya prolifera* JEL478 | NCBI/JGI | PRJNA207863 | SAMN02746072 | Joint Genome Institute | GCA_001574975.1 Ganpr1 | 2015 | Chang et al. [S3] |
| *Anaeromyces robustus S4* | NCBI | PRJNA330692 | SAMN05421914 | Joint Genome Institute | GCA_002104895.1 Anaeromyces sp. S4 v1.0 | 2017 | Mondo et al. [S4] |
| *Piromyces sp. E2* | NCBI | PRJNA82799 | SAMN00788684 | Joint Genome Institute | GCA_002157105.1 PirspE2 v1.0 | 2017 | Mondo et al. [S4] |
| *Synchytrium microbalum* JEL517 | NCBI | PRJNA576245 | SAMN08987497 | Agriculture and Agri-Food Canada | GCA_006535985.1 ASM653598v1 | 2019 | van de Vossenberg et al. [S5] |
| *Rhizoclosmatium globosum* JEL800 | NCBI | PRJNA330693 | SAMN05421919 | Joint Genome Institute | GCA_002104985.1 Rhihy1 | 2017 | Mondo et al. [S4] |
| *Chytriomyces confervae* CBS 675.73 | NCBI | PRJNA453741 | SAMN08987275 | Agriculture and Agri-Food Canada | GCA_006535975.1 | 2019 | van de Vossenberg et al. [S5] |
| *Spizellomyces punctatus* BR117 | NCBI | PRJNA319336 | SAMN00716787 | Broad Institute | GCA_000182565.2 S_punctatus_V1 | 2016 | Russ et al. [S6] |
| *Globomyces pollinis-pini* Arg68 | Joint Genome Institute | Project ID: 1098991 | Project ID: 1098991 | Joint Genome Institute | Glopol1_AssemblyScaffolds.fasta.gz | 2016 | https://genome.jgi.doe.gov/portal /Glopol1/Glopol1.download.html |
| *Batrachochytrium dendrobatidis* JAM81 | NCBI | PRJNA41157 | SAMN02746048 | Joint Genome Institute | GCA_000203795.1 v1.0 | 2011 | Joneson et al. [7] |
| *Homolaphlyctis polyrhiza* JEL142 | NCBI | PRJNA68115 | SAMN02981350 | University of Idaho | GCA_000235945.1 HOMPOL_1.0 | 2011 | Joneson et al. [7] |
| *Amoeboradix gromovi* X-113 | NCBI | PRJNA668693 | SAMN16418512 | ESE lab, CNRS, Paris-Saclay University | PRJNA668693 | 2021 | Galindo et al.[8] |
| *Sanchytrium tribonematis* X-128 | NCBI | PRJNA668694 | SAMN16418513 | ESE lab, CNRS, Paris-Saclay University | PRJNA668694 | 2021 | Galindo et al.[8] |
| *Allomyces macrogynus* ATCC 38327 | NCBI | PRJNA20563 | SAMN02953744 | Broad Institute | GCA_000151295.1 A_macrogynus_V3 | 2015 | The Genome Sequence of *Allomyces macrogynus* strain ATCC 38327 / Origins of Multicellularity Sequencing Project, Broad Institute of Harvard and MI |
| *Blastocladiella emersonii* ATCC 22665 | NCBI | dbEST-Id 25065829 to 25082878. | dbEST-Id 25065829 to 25082878. | Universidade de São Paulo | dbEST:CO961503-CO978552 | 2005 | Ribichich et al. [S9] |
| *Catenaria anguillulae* PL171 | NCBI | PRJNA330705 | SAMN05421822 | Joint Genome Institute | GCA_002102555.1 Catan2 | 2017 | Mondo et al. [S4] |
| *Olpidium bornovanus* S191 | NCBI | NCBI | SAMN05920846 | Joint Genome Institute | GCA_017911155.1 | 2021 | Chang et al. [S10] |
| *Basidiobolus meristosporus* CBS 931.73 | NCBI | PRJNA196075 | SAMN02745972 | Joint Genome Institute | GCA_002104905.1 Basme2finSC | 2017 | Mondo et al. [S4] |
| *Basidiobolus heterosporus* B8920 | NCBI | PRJNA211915 | SAMN02371514 | Institute for Genome Sciences, University of Maryland | GCA_000697455.1 BasHetB8920-1.0 | 2016 | Chibucos et al.[S11] |
| *Conidiobolus coronatus* NRRL 28638 | NCBI | PRJNA67455 | SAMN00779232 | Joint Genome Institute | GCA_001566745.1 Conidiobolus coronatus NRRL28638 | 2015 | Chang et al. [S3] |
| Entomophthora muscae KVL-14-117 | NCBI | PRJEB10825 | SAMEA3545222 | University of Copenhagen | GCA_900018355.1 Em_KVL-14-118 | 2017 | De Fine Licht et al.[S12] |
| *Coemansia reversa* NRRL 1564 | NCBI | PRJNA68631 | SAMN00631612 | Joint Genome Institute | GCA_002705745.1 Coere1 | 2015 | Chang et al. [S3] |
| Capniomyces stellatus MIS-10-108 | NCBI | PRJNA315385 | SAMN04558131 | University of Toronto | GCA_001661515.1 ASM166151v1 | 2016 | Wang et al.[S13] |
| Glomus cerebriforme DAOM 227022 | NCBI | PRJNA430010 | SAMN08364855 | INRA | GCA_003550305.1 ASM355030v1 | 2019 | Morin et al.[S14] |
| *Rhizophagus irregularis* DAOM 197198 | NCBI | PRJNA471752 | SAMN02744054 | Joint Genome Institute | GCA_000439145.3 ASM43914v3 | 2018 | Chen et al.[S15] |
| *Mortierella antarctica KOD 1229* | NCBI | PRJNA340822 | SAMN05720774 | Joint Genome Institute | GCA_015849325.1_UCR_MspKOD1229_1.0 | 2020 | Vandepol et al.[S16] |
| *Mortiereinala alpina* ATCC 32222 | NCBI | PRJNA41211 | SAMN02981246 | Jiangnan Universit / Nankai University / Wake Forest University School of Medicine | GCA_000240685.2 ASM24068v2 | 2011 | Wang et al.[S17] |
| *Mucor circinelloides* JCM 22480 | NCBI | PRJNA537675 | SAMN11512151 | Joint Genome Institute | GCA_001599575.1 JCM_22480_assembly_v001 | 2019 | Navarro-Mendoza et al.[S18] |
| *Umbelopsis isabellina* B7317 | NCBI | PRJNA211912 | SAMN02370994 | Institute for Genome Sciences, University of Maryland | GCA_000697415.1 UmbIsaB7317-1.0 | 2014 | Chibucos et al.[S11] |
| *Rhizopus delemar* RA 99-880 | NCBI | PRJNA13066 | SAMN02953598 | Broad Institute | GCA_000149305.1_RO3 | 2009 | Ma et al.[S19] |
| *Rhizopus oryzae* 99-892 | NCBI | PRJNA186020 | SAMN02352643 | Institute for Genome Sciences, University of Maryland | GCA_000697725.1 RhiOry99-892-1.0 | 2016 | Chibucos et al.[S11] |
| *Phycomyces blakesleeanus* NRRL 1555 | NCBI | PRJNA342701 | SAMN00189023 | Joint Genome Institute | GCA_001638985.2 Phybl2 | 2016 | Corrochano et al.[S20] |
| *Cryptococcus neoformans* var. grubii H99 | NCBI | PRJNA177334 | SAMN03081441 | Broad Institute/ Duke University | GCA_000149245.3 CNA3 | 2014 | Janbon et al.[S21] |
| *Ustilago maydis* 521 | NCBI | PRJNA14007 | SAMN02900459 | Broad Institute | GCA_000328475.2 Umaydis521_2.0 | 2006 | Kämper et al.[S22] |
| *Puccinia graminis* f. sp. *tritici* CRL 75-36-700-3 | NCBI | PRJNA66375 | SAMN00013043 | Broad Institute | GCA_000149925.1 ASM14992v1 | 2011 | Duplessis et al.[S23] |
| *Coprinopsis cinerea* okayama7#130 | NCBI | PRJNA1447 | SAMN02953595 | Broad Institute | GCF_000182895.1_CC3 | 2010 | Stajich et al.[S24] |
| *Aspergillus nidulans* FGSC A4 | NCBI | PRJNA130 | SAMN02953587 | Broad Institute | GCF_000149205.2_ASM14920v2 | 2005 | Galagan et al.[S25] |

| | | | | | | | |
|---|---|---|---|---|---|---|---|
| *Trichoderma atroviride* IMI 206040 | NCBI | PRJNA19867 | SAMN02744066 | Joint Genome Institute | GCF_000171015.1_TRIAT_v2.0 | 2011 | Kubicek et al.[S26] |
| *Neurospora crassa* OR74A | NCBI | PRJNA13841 | SAMN02953583 | Broad Institute | GCF_000182925.2_NC12 | 2003 | Galagan et al.[S27] |
| *Fusarium oxysporum* NRRL 32931 | NCBI | PRJNA67067 | SAMN02981331 | Broad Institute | GCF_000271745.1_FO_FOSC_3_a_V1 | 2018 | DeIulio et al.[S28] |
| *Saitoella complicata* NRRL Y-17804 | NCBI | PRJNA352842 | SAMN00760712 | Joint Genome Institute | GCA_001661265.1 Saico1 | 2016 | Riley et al.[S29] |
| *Sclerotinia sclerotiorum* 1980 UF-70 | NCBI | PRJNA15530 | SAMN02953621 | Broad Institute | GCA_000146945.2 ASM14694v2 | 2011 | Amselem et al.[S30] |
| *Saccharomyces cerevisiae* S288C | NCBI | PRJNA128 | PRJNA128 | Saccharomyces Genome Database | GCA_000146045.2 | 1996 | Goffeau et al.[S31] |
| *Schizosaccharomyces pombe* (ASM294v2) | NCBI | PRJNA127 | SAMEA3138176 | Universite de Montreal | GCA_000002945.2 ASM294v2 | 2007 | Wood et al.[S32] |

## Table S1. List of the genomes/proteomes used in this study, Related to STAR Methods.

| Taxon | Publication | Year | Light or Electron Microscopy? |
|---|---|---|---|
| *Blastocladiella emersonii* | Reichle et al.[S33] | 1967 | Light and Electron Microscopy |
| *Blastocladiella emersonii* | Cantino et al.[S34] | 1970 | Electron Microscopy |
| *Catenaria anguillulae* | Manier[S35] | 1977 | Electron Microscopy |
| *Allomyces macrogynus* | Fuller et al.[S36] | 1971 | Electron Microscopy |
| *Sanchytrium tribonematis* | Karpov et al.[S37] | 2019 | Light and Electron Microscopy |
| *Amoeboradix gromovi* | Karpov et al.[S38] | 2018 | Light and Electron Microscopy |
| *Chytriomyces confervae* | Barr et al.[S39] | 1976 | Electron Microscopy |
| *Globomyces pollinis-pini* | Letcher et al.[S40] | 2008 | Light and Electron Microscopy |
| *Rhizoclosmatium globosum* | Barr et al.[S39] | 1976 | Electron Microscopy |
| *Synchytrium microbalum* | Longcore et al.[S41] | 2016 | Light and Electron Microscopy |
| *Gonapodya prolifera* | Mollicone et al.[S42] | 1999 | Light and Electron Microscopy |

**Table S2. Origin of the microscopic data used to discuss and represent the lipid droplets in Zoospores, Related to Figure 1 and STAR Methods.**

| | CyclOp pathway | | | | | | | |
|---|---|---|---|---|---|---|---|---|
| Species and strain | CyclOp | evalue | Rhodopsin type I | evalue | GC1 | evalue | BeCNG1 | evalue |
| Paraphelidium tribonemae X-108 | NF | NF | NF | NF | NF | NF | NF | NF |
| Gonapodya prolifera JEL478 | >KXS22530.1_adenylyl_cyclase_[Gonapodya_prolifera_JEL478] | 1.00E-95 | PARTIAL >KXS22530.1_adenylyl_cyclase_[Gonapodya_prolifera_JEL478] | | >KXS22530.1_adenylyl_cyclase_[Gonapodya_prolifera_JEL478] | 4.00E-83 | NF | NF |
| Anaeromyces robustus S4 | NF | NF | NF | NF | NF | NF | NF | NF |
| Piromyces sp. E2 | NF | NF | NF | NF | NF | NF | NF | NF |
| Synchytrium microbalum JEL517 | XP_031023840.1 uncharacterized protein SmJEL517_g04188, XP_031024454.1 uncharacterized protein SmJEL517_g03632 | 0.00E+00 | XP_031023840.1 uncharacterized protein SmJEL517_g04188, XP_031024454.1 uncharacterized protein SmJEL517_g03632 | 2E-27, 9e-12 | XP_031023840.1 uncharacterized protein SmJEL517_g04188, XP_031024454.1 uncharacterized protein SmJEL517_g03632 | 2e-102, 1e-85 | XP_031027475.1 uncharacterized protein SmJEL517_g00458 | 2.00E-118 |
| Rhizoclosmatium globosum JEL800 | ORY50000.1 adenylyl cyclase [Rhizoclosmatium globosum], ORY50001.1 adenylyl cyclase [Rhizoclosmatium globosum], ORY49999.1 adenylyl cyclase [Rhizoclosmatium globosum] | 1e-141, 9e-138, 5e-107 | ORY50000.1 adenylyl cyclase [Rhizoclosmatium globosum], ORY50001.1 adenylyl cyclase [Rhizoclosmatium globosum], ORY49999.1 adenylyl cyclase [Rhizoclosmatium globosum] | 7e-19, 5e-18, 5e-04 | ORY50000.1 adenylyl cyclase [Rhizoclosmatium globosum], ORY50001.1 adenylyl cyclase [Rhizoclosmatium globosum], ORY49999.1 adenylyl cyclase [Rhizoclosmatium globosum] | 1e-93, 3e-93, 1e-86 | ORY41228.1 hypothetical protein BCR33DRAFT_718890, ORY41095.1 camp-binding domain-like protein | 2e-125, 2e-119 |
| Chytriomyces confervae CBS 675.73 | TPX69866.1 hypothetical protein CcCBS67573_g06742, TPX74849.1 hypothetical protein CcCBS67573_g03867, TPX78280.1 hypothetical protein CcCBS67573_g00474 | 2e-141, 8e-141, 3e-135 | TPX69866.1 hypothetical protein CcCBS67573_g06742, TPX74849.1 hypothetical protein CcCBS67573_g03867, TPX78280.1 hypothetical protein CcCBS67573_g00474 | 3e-22, 9e-22, 2e-20 | TPX69866.1 hypothetical protein CcCBS67573_g06742, TPX74849.1 hypothetical protein CcCBS67573_g03867, TPX78280.1 hypothetical protein CcCBS67573_g00474 | 3e-90, 1e-89, 2e-89 | TPX64874.1 hypothetical protein CcCBS67573_g08290 | 4.00E-165 |
| Spizellomyces punctatus BR117 | NF | NF | NF | NF | NF | NF | XP_016611400.1_hypothetical_protein_SPPG_02404 | 1.00E-156 |
| Globomyces pollinis-pini Arg68 | jgi\|Glopol1\|618394\|gm1.2182_g, jgi\|Glopol1\|131290\|CE131289_10627 | 8e-129, 3e-111 | jgi\|Glopol1\|618394\|gm1.2182_g, jgi\|Glopol1\|131290\|CE131289_10627 | 2.00E-21 | jgi\|Glopol1\|618394\|gm1.2182_g, jgi\|Glopol1\|131290\|CE131289_10627 | 2e-99, 1e-96 | NF | NF |
| Batrachochytrium dendrobatidis JAM81 | NF | NF | NF | NF | NF | NF | NF | NF |
| Homolaphlyctis polyrhiza JEL142 | NF | NF | >AFSM01002127.1_Homolaphlyctis_polyrhiza_JEL_142_strain_JEL142_JEL142_contig02416,_whole_genome_shotgun_sequence.p1 type:complete len:162 gc:universal | 8.00E-09 | >AFSM01002127.1_Homolaphlyctis_polyrhiza_JEL_142_strain_JEL142_JEL142_contig02416,_whole_genome_shotgun_sequence.p2 type:complete len:159 gc:universal | 1.00E-74 | NF | NF |
| Amoeboradix gromovi X-113 | NODE_640_length_5666_cov_0.633196.p2 type:complete len:532 | 6.00E-128 | NODE_640_length_5666_cov_0.633196.p2 type:complete len:532 | 6.00E-40 | NODE_640_length_5666_cov_0.633196.p2 type:complete len:532 | 9.00E-95 | NODE_19_length_39463_cov_0.396073.p9 type:complete len:394 | 9.00E-97 |
| Sanchytrium tribonematis X-128 | NODE_27_length_39007_cov_1.590650.p8_type:complete_len:535_gc | 1.00E-125 | NODE_27_length_39007_cov_1.590650.p8_type:complete_len:535_gc | 1.00E-40 | NODE_27_length_39007_cov_1.590650.p8_type:complete_len:535_gc | 1.00E-101 | NODE_1456_length_1026_cov_0.812298.p1_type:3prime_partial_len | 0.00E+00 |
| Allomyces macrogynus ATCC 38327 | KNE62746.1_hypothetical_protein_AMAG_07932, KNE65585.1_hypothetical_protein_AMAG_09565 | 1e-140, 6e-137 | KNE62746.1_hypothetical_protein_AMAG_07932, KNE65585.1_hypothetical_protein_AMAG_09565 | 2e-63, 1e-62 | KNE62746.1_hypothetical_protein_AMAG_07932, KNE65585.1_hypothetical_protein_AMAG_09565 | 3e-144, 5e-143 | KNE62879.1_hypothetical_protein_AMAG_18930, KNE61876.1_hypothetical_protein_AMAG_07148 | 0.00E+00 |
| Blastocladiella emersonii ATCC 22665 | gi\|59276301\|gb\|CO961585.1\|CO961585.p2 type:internal len:223 | 2.00E-84 | gi\|59276301\|gb\|CO961585.1\|CO961585.p2 type:internal len:223 | 1.00E-12 | gi\|59276301\|gb\|CO961585.1\|CO961585.p2 type:internal len:223 | 4.00E-100 | gi\|148886264\|gb\|EE736522.1\|EE7365 | 0.00E+00 |

| | | | | | | | | |
|---|---|---|---|---|---|---|---|---|
| | | | | | | | 22.p1 type:internal len:119 | |
| Catenaria anguillulae PL171 | ORZ32123.1_nucleotide_cyclase_[Catenaria_anguillulae_PL171] | 2.00E-101 | ORZ32123.1_nucleotide_cyclase_[Catenaria_anguillulae_PL171] | 2.00E-53 | ORZ32123.1_nucleotide_cyclase_[Catenaria_anguillulae_PL171] | 5.00E-143 | ORZ36627.1_hypothetical_protein_BCR44DRAFT_1512424 | 0.00E+00 |
| Olpidium bornovanus S191 | NF | NF | NF | NF | NF | NF | NF | NF |
| Basidiobolus meristosporus CBS 931.73 | NF | NF | NF | NF | NF | NF | NF | NF |
| Basidiobolus heterosporus B8920 | NF | NF | NF | NF | NF | NF | NF | NF |
| Conidiobolus coronatus NRRL 28638 | NF | NF | NF | NF | NF | NF | NF | NF |
| Entomophthora muscae | NF | NF | NF | NF | NF | NF | NF | NF |
| Coemansia reversa NRRL 1564 | NF | NF | NF | NF | NF | NF | NF | NF |
| Capniomyces stellatus MIS-10-108 | NF | NF | NF | NF | NF | NF | NF | NF |
| Glomus cerebriforme | NF | NF | NF | NF | NF | NF | NF | NF |
| Rhizophagus irregularis DAOM 197198 | NF | NF | NF | NF | NF | NF | NF | NF |
| Mortierella antarctica | NF | NF | NF | NF | NF | NF | NF | NF |
| Mortiereinala alpina ATCC 32222 | NF | NF | NF | NF | NF | NF | NF | NF |
| Mucor circinelloides JCM 22480 | NF | NF | NF | NF | NF | NF | NF | NF |
| Umbelopsis isabellina B7317 | NF | NF | NF | NF | NF | NF | NF | NF |
| Rhizopus delemar RA 99-880 | NF | NF | NF | NF | NF | NF | NF | NF |
| Rhizopus oryzae 99-892 | NF | NF | NF | NF | NF | NF | NF | NF |
| Phycomyces blakesleeanus NRRL 1555 | NF | NF | NF | NF | NF | NF | NF | NF |
| Cryptococcus neoformans var. grubii H99 | NF | NF | NF | NF | NF | NF | NF | NF |
| Ustilago maydis 521 | NF | NF | NF | NF | NF | NF | NF | NF |
| Puccinia graminis f. sp. tritici CRL 75-36-700-3 | NF | NF | NF | NF | NF | NF | NF | NF |
| Coprinopsis cinerea okayama7#130 | NF | NF | NF | NF | NF | NF | NF | NF |
| Aspergillus nidulans FGSC A4 | NF | NF | NF | NF | NF | NF | NF | NF |
| Trichoderma atroviride IMI 206040 | NF | NF | NF | NF | NF | NF | NF | NF |
| Neurospora crassa OR74A | NF | NF | NF | NF | NF | NF | NF | NF |
| Fusarium oxysporum NRRL 32931 | NF | NF | NF | NF | NF | NF | NF | NF |
| Saitoella complicata NRRL Y-17804 | NF | NF | NF | NF | NF | NF | NF | NF |
| Sclerotinia sclerotiorum 1980 UF-70 | NF | NF | NF | NF | NF | NF | NF | NF |
| Saccharomyces cerevisiae S288C | NF | NF | NF | NF | NF | NF | NF | NF |

| | | | | | | | | |
|---|---|---|---|---|---|---|---|---|
| Schizosaccharomyces pombe (ASM294v2) | NF | NF | NF | NF | NF | NF | NF | NF |

| | Carotenoid biosynthesis | | | | |
|---|---|---|---|---|---|
| **Species and strain** | **Phytoene dehydrogenase-related protein** | **evalue** | **Phytoene synthase** | **evalue** | **Carotenoid oxygenase** |
| Paraphelidium tribonemae X-108 | NF | NF | NF | NF | Partr_v1_DN27125_c1_g1_i1_m15640_putative_dioxygenase |
| Gonapodya prolifera JEL478 | NF | NF | NF | NF | >KXS15063.1 hypothetical protein M427DRAFT_70166 [Gonapodya prolifera JEL478] |
| Anaeromyces robustus S4 | NF | NF | NF | NF | ORX81586.1_hypothetical_protein_BCR32DRAFT_279584_[Anaeromyces |
| Piromyces sp. E2 | NF | NF | NF | NF | ORX81586.1 |
| Synchytrium microbalum JEL517 | >XP_031025712.1 uncharacterized protein SmJEL517_g02419 | 0.00E+00 | NF | NF | >XP_031025338.1 uncharacterized protein SmJEL517_g02720 [Synchytrium microbalum] |
| Rhizoclosmatium globosum JEL800 | >ORY40098.1 phytoene desaturase [Rhizoclosmatium globosum] | 1.00E-153 | >ORY52898.1 terpenoid synthase [Rhizoclosmatium globosum] | 7.00E-90 | >ORY47689.1 hypothetical protein BCR33DRAFT_735871 [Rhizoclosmatium globosum] |
| Chytriomyces confervae CBS 675.73 | >TPX74165.1 hypothetical protein CcCBS67573_g04564 [Chytriomyces confervae] | 1.00E-178 | >TPX77582.1 hypothetical protein CcCBS67573_g01152 | 9.00E-49 | >TPX66659.1 hypothetical protein CcCBS67573_g07753 [Chytriomyces confervae] |
| Spizellomyces punctatus BR117 | NF | NF | NF | NF | XP_016607807.1_hypothetical_protein_SPPG_05143 |
| Globomyces pollinis-pini Arg68 | jgi\|Glopol1\|529184\|e_gw1.200.18.1 | 0.00E+00 | jgi\|Glopol1\|573654\|fgenesh1_kg.6_#_299_#_TRINITY_DN5419_c0_g1_i1 | 3.00E-60 | NF |
| Batrachochytrium dendrobatidis JAM81 | NF | NF | NF | NF | NF |
| Homolaphlyctis polyrhiza JEL142 | JH393157.1_Rhizophydiales_sp._JEL142_unplaced_genomic_scaffold | 3.00E-56 | AFSM01007168.1_Homolaphlyctis_polyrhiza_JEL_142_strain_JEL142_J | 3.00E-23 | NF |
| Amoeboradix gromovi X-113 | NF | NF | NF | NF | NF |
| Sanchytrium tribonematis X-128 | NF | NF | NF | NF | NF |
| Allomyces macrogynus ATCC 38327 | >KNE66202.1_phytoene_desaturase_[Allomyces_macrogynus_ATCC_38327] / >KNE57458.1_phytoene_desaturase_[Allomyces_macrogynus_ATCC_38327] | 0.00E+00 | >KNE57899.1_lycopene_cyclase_domain-containing_protein_[Allomyces_macrogynus_ATCC_38327] / >KNE59078.1_lycopene_cyclase_domain-containing_protein_[Allomyces_macrogynus_ATCC_38327] | 0.00E+00 | >KNE55150.1_hypothetical_protein_AMAG_01073_[Allomyces_macrogynus_ATCC_38327] / >KNE59794.1_hypothetical_protein_AMAG_05253_[Allomyces_macrogynus_ATCC_38327] |
| Blastocladiella emersonii ATCC 22665 | >AIC07010.1_putative_phytoene_dehydrogenase_[Blastocladiella_emersonii] | 0.00E+00 | >AIC07009.1_putative_lycopene_cyclase_phytoene_synthase_[Blastocladiella_emersonii] | 0.00E+00 | >AIC07011.1_putative_carotenoid_dioxygenase_[Blastocladiella_emersonii] |
| Catenaria anguillulae PL171 | >ORZ34110.1_hypothetical_protein_BCR44DRAFT_1479970_[Catenaria_anguillulae_PL171] | 0.00E+00 | >ORZ39450.1_Squalene/phytoene_synthase-domain-containing_protein_[Catenaria_anguillulae_PL171] | 1.00E-66 | >ORZ40243.1_retinal_pigment_epithelial_membrane_protein-domain-containing_protein_[Catenaria_anguillulae_PL171] / ORZ31212.1_carotenoid_oxygenase_[Catenaria_anguillulae_PL171] |
| Olpidium bornovanus S191 | NF | NF | NF | NF | NF |
| Basidiobolus meristosporus CBS 931.73 | NF | NF | NF | NF | ORY02234.1 carotenoid oxygenase [Basidiobolus meristosporus |
| Basidiobolus heterosporus B8920 | NF | NF | NF | NF | JNET01030896.1_Basidiobolus_heterosporus_B8920_jtg7180000624177 |
| Conidiobolus coronatus NRRL 28638 | NF | NF | NF | NF | NF |
| Entomophthora muscae | NF | NF | NF | NF | NF |
| Coemansia reversa NRRL 1564 | NF | NF | NF | NF | PIA18333.1_hypothetical_protein_COEREDRAFT_79821 |
| Capniomyces stellatus MIS-10-108 | NF | NF | NF | NF | NF |
| Glomus cerebriforme | NF | NF | NF | NF | RIA91701.1 carotenoid oxygenase, partial [Glomus cerebriforme] |
| Rhizophagus irregularis DAOM 197198 | NF | NF | NF | NF | XP_025172812.1 hypothetical protein GLOIN_2v1781123 |
| Mortierella antarctica | NF | NF | NF | NF | KAF9987450.1 hypothetical protein BGZ75_000607 |
| Mortiereinala alpina ATCC 32222 | NF | NF | NF | NF | ADAG01001055.1\|m.19462_ADAG01001055.1\|g.19462_type:complete |

| | | | | | |
|---|---|---|---|---|---|
| Mucor circinelloides JCM 22480 | KAF1801867.1 phytoene dehydrogenase [Mucor circinelloides] | 0.00E+00 | KAF1801868.1 Lycopene beta-cyclase [Mucor circinelloides] | 3.00E-95 | KAF1807426.1 putative carotene oxygenase [Mucor circinelloides] |
| Umbelopsis isabellina B7317 | JNEQ01000058.1_Umbelopsis_isabellina_B7317_jtg7180000025250f_71 | 1.00E-123 | JNEQ01000058.1_Umbelopsis_isabellina_B7317_jtg7180000025250f_71 | 2.00E-42 | JNEQ01000040.1_Umbelopsis_isabellina_B7317_ctg7180000025259_1 |
| Rhizopus delemar RA 99-880 | EIE91958.1 hypothetical protein RO3G_16669 | 0.00E+00 | EIE78626.1 hypothetical protein RO3G_03330 | 7.00E-56 | NF |
| Rhizopus oryzae 99-892 | KK997965.1\|m.1783 KK997965.1\|g.1783 type:complete len:288 gc | 4.00E-105 | KK997965.1\|m.1782 KK997965.1\|g.1782 type:complete len:313 gc | 4.00E-36 | KK998411.1\|m.11289 KK998411.1\|g.11289 type:complete len:426 |
| Phycomyces blakesleeanus NRRL 1555 | XP_018294564.1 Phytoene dehydrogenase | 0.00E+00 | XP_018294563.1 Phytoene Synthase/Lycopene cyclase | 5.00E-89 | XP_018284903.1 hypothetical protein PHYBLDRAFT_183749 |
| Cryptococcus neoformans var. grubii H99 | NF | NF | NF | NF | NF |
| Ustilago maydis 521 | XP_011390692.1 putative phytoene dehydrogenase | 2.00E-165 | XP_011388293.1 hypothetical protein UMAG_06287 | 4.00E-45 | NF |
| Puccinia graminis f. sp. tritici CRL 75-36-700-3 | XP_003337345.2 hypothetical protein PGTG_19044 | 3.00E-180 | XP_003329970.2 hypothetical protein PGTG_11907 | 6.00E-56 | NF |
| Coprinopsis cinerea okayama7#130 | NF | NF | NF | NF | NF |
| Aspergillus nidulans FGSC A4 | NF | NF | NF | NF | XP_659308.1 hypothetical protein AN1704.2 |
| Trichoderma atroviride IMI 206040 | NF | NF | NF | NF | XP_013942699.1 hypothetical protein TRIATDRAFT_223967 |
| Neurospora crassa OR74A | XP_964713.1 phytoene desaturase [Neurospora crassa OR74A] | 8.00E-148 | XP_965725.3 phytoene synthase [Neurospora crassa OR74A] | 6.00E-60 | XP_001727958.1 carotenoid oxygenase 2 [Neurospora crassa OR74A] |
| Fusarium oxysporum NRRL 32931 | XP_031030490.1 uncharacterized protein FOYG_15803 | 3.00E-174 | XP_031030489.1 uncharacterized protein FOYG_15802 | 6.00E-76 | XP_031040765.1 uncharacterized protein FOYG_08800 |
| Saitoella complicata NRRL Y-17804 | XP_019021262.1 phytoene dehydrogenase | 0.00E+00 | XP_019022354.1 terpenoid synthase | 2.00E-67 | NF |
| Sclerotinia sclerotiorum 1980 UF-70 | XP_001594533.1 hypothetical protein SS1G_04340 | 9.00E-158 | XP_001594534.1 hypothetical protein SS1G_04341 | 3.00E-12 | NF |
| Saccharomyces cerevisiae S288C | NF | NF | NF | NF | NF |
| Schizosaccharomyces pombe (ASM294v2) | NF | NF | NF | NF | NF |

| | Photoreceptors | | | | | | | |
|---|---|---|---|---|---|---|---|---|
| **Species and strain** | **WC-1 (White Collar 1)** | **evalue** | **WC-2 (White Collar 2)** | **evalue** | **VVD (VIVID)** | **evalue** | **CRY (cryptochrome)** | **evalue** |
| Paraphelidium tribonemae X-108 | NF | NF | NF | NF | NF | NF | Partr_v1_DN26245_c1_g1_i3_m48392_putative_cryptochrome | 2.00E-30 |
| Gonapodya prolifera JEL478 | KXS17110.1 hypothetical protein M427DRAFT_153942 | 4E-54, 2e-44 | KXS18876.1 hypothetical protein M427DRAFT_53357, KXS09899.1 hypothetical protein M427DRAFT_149316 | 3e-16, 4e-12 | NF | NF | KXS15620.1 cryptochrome [Gonapodya prolifera JEL478] | 5.00E-73 |
| Anaeromyces robustus S4 | NF | NF | NF | NF | NF | NF | NF | NF |
| Piromyces sp. E2 | NF | NF | NF | NF | NF | NF | NF | NF |
| Synchytrium microbalum JEL517 | XP_031027930.1 uncharacterized protein SmJEL517_g00414 | 5.00E-105 | XP_031026964.1 uncharacterized protein SmJEL517_g01144 | 3.00E-09 | NF | NF | XP_031023438.1 uncharacterized protein SmJEL517_g04615 | 2.00E-87 |
| Rhizoclosmatium globosum JEL800 | NF | NF | NF | NF | NF | NF | ORY50805.1 cryptochrome [Rhizoclosmatium globosum] | 2.00E-65 |
| Chytriomyces confervae CBS 675.73 | TPX74047.1 hypothetical protein CcCBS67573_g04675 | 2.00E-66 | TPX53564.1 hypothetical protein CcCBS67573_g09686 | 1.00E-13 | NF | NF | TPX70061.1 hypothetical protein CcCBS67573_g06678 | 2.00E-56 |
| Spizellomyces punctatus BR117 | XP_016610190.1_hypothetical_protein_SPPG_09046 | 3.00E-84 | XP_016606504.1_PAS_domain_S-box_protein, XP_016612182.1_white_collar_2_protein | 8e-17, 3e-13 | NF | NF | NF | NF |
| Globomyces pollinis-pini Arg68 | jgi\|Glopol1\|579169\|fgenesh1_kg.24_#_249_#_TRINITY_DN4747_c0_g1_i7 | 1.00E-74 | jgi\|Glopol1\|437558\|CE437557_9169 | 2.00E-14 | NF | NF | NF | NF |
| Batrachochytrium | NF | NF | NF | NF | NF | NF | NF | NF |

| | | | | | | | | |
|---|---|---|---|---|---|---|---|---|
| dendrobatidis JAM81 | | | | | | | | |
| Homolaphlyctis polyrhiza JEL142 | NF | NF | NF | NF | NF | NF | NF | NF |
| Amoeboradix gromovi X-113 | NODE_13_length_42895_cov_0.518413.p8 type:complete l | 6.00E-70 | NODE_154_length_16838_cov_0.293327.p8 type:complete len:220 gc | 6.00E-22 | NF | NF | NODE_14_length_42432_cov_0.433799.p8 type:complete len:490 | 1.00E-15 |
| Sanchytrium tribonematis X-128 | NODE_754_length_4199_cov_1.046829.p1_type:complete_len:558_gc:universal, NODE_244_length_11882_cov_0.560214.p4_type:complete_len:343_gc, NODE_1173_length_1905_cov_0.460133.p1_type:3prime_partial_len | 4E-62, 1e-59, 5e-51 | NODE_287_length_10726_cov_0.240614.p3_type:complete_len:219_gc, NODE_10_length_61504_cov_0.956502.p34_type:complete_len:204_gc | 3.00E-23 | NF | NF | NODE_31_length_37178_cov_0.413576.p6_type:complete_len:479 , NODE_911_length_3104_cov_0.287188.p1_type:complete_len:509 | 2e-71, 4e-20 |
| Allomyces macrogynus ATCC 38327 | KNE69080.1_PAS_domain_S-box_protein | 1.00E-53 | KNE64375.1_PAS_domain_S-box_protein, KNE70175.1_hypothetical_protein_AMAG_15144 | 9E-20, 5e-14 | NF | NF | KNE59778.1_hypothetical_protein_AMAG_05242, | 2.00E-25 |
| Blastocladiella emersonii ATCC 22665 | NF | NF | gi\|59287008\|gb\|CO972292.1\|CO972292.p1 type:internal | 1.00E-08 | NF | NF | NF | NF |
| Catenaria anguillulae PL171 | ORZ34267.1_PAS_domain-domain-containing_protein_[Catenaria_ | 1.00E-119 | ORZ34902.1_hypothetical_protein_BCR44DRAFT_49868 | 1.00E-18 | NF | NF | ORZ31450.1_FAD_binding_domain_of_DNA_photolyase-domain-containing_protein, ORZ37966.1_DNA_photolyase,_FAD-binding/Cryptochrome | 5E-29, 1e-20 |
| Olpidium bornovanus S191 | KAG5462463.1 hypothetical protein BJ554DRAFT_5016, partial [Olpidium bornovanus] | 3.00E-30 | NF | NF | NF | NF | NF | NF |
| Basidiobolus meristosporus CBS 931.73 | ORY03092.1 hypothetical protein K493DRAFT_311836 [Basidiobolus meristosporus CBS 931.73], ORX92749.1 hypothetical protein K493DRAFT_285400, ORX78940.1 hypothetical protein K493DRAFT_293436, ORX82576.1 hypothetical protein K493DRAFT_270021, ORX82533.1 hypothetical protein K493DRAFT_91526, ORY05666.1 hypothetical protein K493DRAFT_274936, ORY06962.1 hypothetical protein K493DRAFT_251462 | 3E-162, 2e-135, 3e-134, 4e-131, 6e-131, 2e-129, 3e-128 | ORY05759.1 hypothetical protein K493DRAFT_159907, ORX90491.1 hypothetical protein K493DRAFT_317922, ORY00637.1 hypothetical protein K493DRAFT_278641 | 4E-35, 3e-34, 6e-34 | NF | NF | ORX90230.1 cryptochrome [Basidiobolus meristosporus CBS 931.73], ORX77140.1 cryptochrome [Basidiobolus meristosporus CBS 931.73] | 3e-84, 4e-84 |
| Basidiobolus heterosporus B8920 | JNET01032621.1_Basidiobolus_heterosporus_B8920_jtg7180000793710 | 7.00E-164 | JNET01033074.1_Basidiobolus_heterosporus_B8920_jtg7180000785454 | 3.00E-36 | NF | NF | JNET01017388.1_Basidiobolus_heterosporus_B8920_ctg7180000781388 | 1.00E-24 |
| Conidiobolus coronatus NRRL 28638 | KXN70665.1 hypothetical protein CONCODRAFT_75611, KXN67054.1 hypothetical protein CONCODRAFT_19863 | 8E-141, 2e-82 | KXN71654.1 hypothetical protein CONCODRAFT_57247 [Conidiobolus | 1.00E-37 | NF | NF | NF | NF |
| Entomophthora muscae | KAF7747831.1 blue light receptor [Entomophthora muscae], KAF7750602.1 blue light receptor [Entomophthora muscae, KAF7748558.1 blue light receptor [Entomophthora muscae] | 2E-126, 2e-118, 1e-117 | KAF7744128.1 blue light receptor [Entomophthora muscae], KAF7744124.1 blue light receptor [Entomophthora muscae] | 5e-41, 5e-41 | NF | NF | KAF7758463.1 hypothetical protein DSO57_009719, KAF7723467.1 hypothetical protein DSO57_008515 | 4e-31, 5e-31 |
| Coemansia reversa NRRL 1564 | NF | NF | NF | NF | NF | NF | NF | NF |
| Capniomyces stellatus MIS-10-108 | NF | NF | NF | NF | NF | NF | NF | NF |
| Glomus cerebriforme | RIA85642.1 PAS domain-containing protein [Glomus cerebriforme] | 8.00E-170 | RIA81527.1 hypothetical protein C1645_810010 [Glomus cerebriforme] | 1.00E-58 | NF | NF | NF | NF |
| Rhizophagus irregularis DAOM 197198 | XP_025178457.1 hypothetical protein GLOIN_2v1604095 | 1.00E-164 | XP_025179286.1 hypothetical protein GLOIN_2v1477793 | 2.00E-39 | NF | NF | ORY50805.1 cryptochrome [Rhizoclosmatium globosum] | 2.00E-65 |
| Mortierella antarctica | NF | NF | NF | NF | NF | NF | NF | NF |
| Mortiereinala alpina ATCC 32222 | NF | NF | NF | NF | NF | NF | KAF9986046.1 hypothetical protein BGZ75_002275 | 1.00E-21 |

<table>
<tr><td>Mucor circinelloides JCM 22480</td><td>KAF1797780.1 hypothetical protein FB192DRAFT_1333251, KAF1804453.1 hypothetical protein FB192DRAFT_1357808, KAF1802616.1 putative white-collar-1b protein</td><td>3e-149, 2e-139, 5e-131</td><td>KAF1800623.1 hypothetical protein FB192DRAFT_1459016</td><td>1.00E-50</td><td>NF</td><td>NF</td><td>KAF1806586.1 hypothetical protein FB192DRAFT_1351151</td><td>4.00E-81</td></tr>
<tr><td>Umbelopsis isabellina B7317</td><td>JNEQ01000052.1_Umbelopsis_isabellina_B7317_ctg7180000025278_1</td><td>6.00E-85</td><td>JNEQ01000055.1_Umbelopsis_isabellina_B7317_jtg7180000025233f_71, JNEQ01000041.1_Umbelopsis_isabellina_B7317_ctg7180000025260_</td><td>6e-40, 3e-35</td><td>NF</td><td>NF</td><td>NF</td><td>NF</td></tr>
<tr><td>Rhizopus delemar RA 99-880</td><td>EIE91627.1 hypothetical protein RO3G_16338 [Rhizopus delemar, EIE85287.1 hypothetical protein RO3G_09997 [Rhizopus delemar, EIE89562.1 hypothetical protein RO3G_14273 [Rhizopus delemar</td><td>2e-145, 1e-131, 3e-112</td><td>EIE76746.1 hypothetical protein RO3G_01450, EIE79173.1 hypothetical protein RO3G_03878, EIE77397.1 hypothetical protein RO3G_02101, EIE89380.1 hypothetical protein RO3G_14091, EIE77889.1 hypothetical protein RO3G_02593</td><td>2e-29, 5e-29, 6e-29, 6e-27, 1e-23</td><td>NF</td><td>NF</td><td>EIE91586.1 hypothetical protein RO3G_16297</td><td>6.00E-66</td></tr>
<tr><td>Rhizopus oryzae 99-892</td><td>KK998731.1|m.24138 KK998731.1|g.24138</td><td>2.00E-63</td><td>KK998731.1|m.24138 KK998731.1|g.24138 type:complete len:355, KK997983.1|m.2044 KK997983.1|g.2044 type:complete len:350, KK998731.1|m.24143 KK998731.1|g.24143 type:complete len:269, KK998423.1|m.11777 KK998423.1|g.11777 type:complete len:251</td><td>2e-63, 4e-39, 1e-30,1e-29</td><td>NF</td><td>NF</td><td>KK997983.1|m.2048 KK997983.1|g.2048 type:complete len:333</td><td>7.00E-53</td></tr>
<tr><td>Phycomyces blakesleeanus NRRL 1555</td><td>XP_018283657.1 GATA-type zinc finger transcription factor, XP_018295305.1 hypothetical protein PHYBLDRAFT_76314, XP_018288826.1 GATA-type zinc finger transcription factor</td><td>6e-153, 2e-130, 4e-118</td><td>XP_018290827.1 GATA-type zinc finger transcription factor, XP_018296558.1 GATA-type zinc finger transcription factor, XP_018291784.1 GATA-type zinc finger transcription factor, XP_018296714.1 GATA-type zinc finger transcription factor</td><td>1e-53, 7e-51, 7e-26, 3e-25</td><td>NF</td><td>NF</td><td>XP_018298182.1 hypothetical protein PHYBLDRAFT_85761</td><td>6.00E-82</td></tr>
<tr><td>Cryptococcus neoformans var. grubii H99</td><td>XP_012048765.1 white collar 1 protein</td><td>1.00E-110</td><td>XP_012050148.1 white collar 2 protein [Cryptococcus neoformans]</td><td>8.00E-14</td><td>NF</td><td>NF</td><td>NF</td><td>NF</td></tr>
<tr><td>Ustilago maydis 521</td><td>XP_011389633.1 hypothetical protein UMAG_03180</td><td>1.00E-94</td><td>XP_011389049.1 hypothetical protein UMAG_02664</td><td>9.00E-31</td><td>NF</td><td>NF</td><td>XP_011392255.1 hypothetical protein UMAG_05917, XP_011387082.1 hypothetical protein UMAG_01131</td><td>6e-87, 3e-84</td></tr>
<tr><td>Puccinia graminis f. sp. tritici CRL 75-36-700-3</td><td>XP_003321153.2 hypothetical protein PGTG_02195</td><td>3.00E-88</td><td>XP_003321153.2 hypothetical protein PGTG_02195</td><td>4.00E-32</td><td>NF</td><td>NF</td><td>XP_003326630.1 hypothetical protein PGTG_07608</td><td>3.00E-100</td></tr>
<tr><td>Coprinopsis cinerea okayama7#130</td><td>XP_001832659.1 photoreceptor A [Coprinopsis cinerea okayama7#130]</td><td>1.00E-72</td><td>XP_001831710.2 hypothetical protein CC1G_12230</td><td>2.00E-12</td><td>NF</td><td>NF</td><td>NF</td><td>NF</td></tr>
<tr><td>Aspergillus nidulans FGSC A4</td><td>XP_661040.1 hypothetical protein AN3436.2</td><td>3.00E-157</td><td>XP_661211.1 hypothetical protein AN3607.2</td><td>4.00E-60</td><td>XP_661039.1 hypothetical protein AN3435.2</td><td>2.00E-53</td><td>NF</td><td>NF</td></tr>
<tr><td>Trichoderma atroviride IMI 206040</td><td>XP_013938043.1 blue light photoreceptor BLR1</td><td>0.00E+00</td><td>XP_013938453.1 blue light receptor BLR2</td><td>6.00E-147</td><td>XP_013942393.1 putative PAS-domain protein envoy</td><td>3.00E-43</td><td>XP_013941790.1 photolyase [Trichoderma atroviride IMI 206040]</td><td>5.00E-99</td></tr>
<tr><td>Neurospora crassa OR74A</td><td>XP_011395153.1 white collar 1 protein, variant 3, XP_011395152.1 white collar 1 protein, variant 1, XP_011395152.1 white collar 1 protein, variant 2, XP_011395151.1 white collar 1 protein</td><td>0.00E+00</td><td>XP_963819.3 zinc finger white collar protein WC2</td><td>0.00E+00</td><td>AAK08514.1_vivid_PAS_protein_VVD_[Neurospora_crassa]</td><td>0.00E+00</td><td>XP_965722.3 cryptochrome DASH [Neurospora crassa OR74A]</td><td>0.00E+00</td></tr>
<tr><td>Fusarium oxysporum NRRL 32931</td><td>XP_031050442.1 uncharacterized protein FOYG_01134</td><td>0.00E+00</td><td>XP_031041715.1 cutinase palindrome-binding protein</td><td>0.00E+00</td><td>XP_031037528.1 uncharacterized protein FOYG_09732</td><td>2.00E-47</td><td>XP_031038092.1 uncharacterized protein FOYG_10105</td><td>0.00E+00</td></tr>
</table>

| | | | | | | | | |
|---|---|---|---|---|---|---|---|---|
| Saitoella complicata NRRL Y-17804 | XP_019027442.1 hypothetical protein SAICODRAFT_106826, XP_019022537.1 hypothetical protein SAICODRAFT_83374, partial | 2E-141, 3e-97 | XP_019027026.1 hypothetical protein SAICODRAFT_16945 | 8.00E-72 | NF | NF | NF | NF |
| Sclerotinia sclerotiorum 1980 UF-70 | XP_001586924.1 hypothetical protein SS1G_11953 | 0.00E+00 | XP_001587208.1 hypothetical protein SS1G_12238 | 2.00E-128 | XP_001596024.1 hypothetical protein SS1G_02240 | 7.00E-42 | XP_001593735.1 hypothetical protein SS1G_05163 | 0.00E+00 |
| Saccharomyces cerevisiae S288C | NF | NF | NF | NF | NF | NF | NF | NF |
| Schizosaccharomyces pombe (ASM294v2) | NF | NF | NF | NF | NF | NF | NF | NF |

| | **Photoreceptors** | | | | | |
|---|---|---|---|---|---|---|
| **Species and strain** | **NOP-1 (Opsin)** | **evalue** | **Type 2 rhodopsin-like GPCR** | **evalue** | **PHY (phytochromes PHY-1 and PHY-2))** | **evalue** |
| Paraphelidium tribonemae X-108 | NF | NF | NF | NF | NF | NF |
| Gonapodya prolifera JEL478 | KXS16496.1 family A G protein-coupled receptor-like protein | 9.00E-27 | KXS11833.1 hypothetical protein M427DRAFT_35407, KXS12111.1 family A G protein-coupled receptor-like protein | 5e-05, 9e-05 | KXS14916.1 hypothetical protein M427DRAFT_123888 | 0.00E+00 |
| Anaeromyces robustus S4 | NF | NF | NF | NF | NF | NF |
| Piromyces sp. E2 | NF | NF | NF | NF | NF | NF |
| Synchytrium microbalum JEL517 | NF | NF | XP_031024018.1 uncharacterized protein SmJEL517_g04095 | 2.00E-04 | XP_031027105.1 uncharacterized protein SmJEL517_g00807 | 2.00E-152 |
| Rhizoclosmatium globosum JEL800 | NF | NF | ORY38136.1 family A G protein-coupled receptor-like protein, ORY32468.1 family A G protein-coupled receptor-like protein, | 2e-08, 8e-08 | NF | NF |
| Chytriomyces confervae CBS 675.73 | NF | NF | TPX66892.1 hypothetical protein CcCBS67573_g07690, TPX66891.1 hypothetical protein CcCBS67573_g07691 | 5e-13, 5e-11 | NF | NF |
| Spizellomyces punctatus BR117 | NF | NF | Spi_pu_XP_016612672.1_hypothetical_protein_SPPG_00350 | 0.00E+00 | XP_016606298.1_hypothetical_protein,_variant_2, Spi_pu_XP_016606299.1_hypothetical_protein,_variant_1, Spi_pu_XP_016606300.1_hypothetical_protein_SPPG_06658 | 1.00E-40 |
| Globomyces pollinis-pini Arg68 | NF | NF | jgi\|Glopol1\|363053 | 6.00E-14 | NF | NF |
| Batrachochytrium dendrobatidis JAM81 | NF | NF | gb\|EGF80153.1\|_hypothetical_protein_BATDEDRAFT_88919 | 0.00E+00 | NF | NF |
| Homolaphlyctis polyrhiza JEL142 | NF | NF | AFSM01000868.1_Homolaphlyctis_polyrhiza_JEL_142_strain | 0.00E+00 | NF | NF |
| Amoeboradix gromovi X-113 | NF | NF | NF | NF | NF | NF |
| Sanchytrium tribonematis X-128 | NF | NF | NF | NF | NF | NF |
| Allomyces macrogynus ATCC 38327 | NF | NF | KNE54739.1_hypothetical_protein_AMAG_00698, KNE56389.1_hypothetical_protein_AMAG_02198 | 0.00E+00 | NF | NF |
| Blastocladiella emersonii ATCC 22665 | NF | NF | gi\|148884807\|gb\|EE735065.1\|EE735065.p2 | 6.00E-27 | NF | NF |
| Catenaria anguillulae PL171 | NF | NF | ORZ32981.1_hypothetical_protein_BCR44DRAFT_90252 | 7.00E-07 | NF | NF |
| Olpidium bornovanus S191 | NF | NF | KAG5455987.1 hypothetical protein BJ554DRAFT_4394 | 1.00E-05 | NF | NF |
| Basidiobolus meristosporus CBS 931.73 | NF | NF | NF | NF | ORX89367.1 hypothetical protein K493DRAFT_305838 | 8.00E-54 |
| Basidiobolus heterosporus B8920 | NF | NF | NF | NF | NF | NF |
| Conidiobolus coronatus NRRL 28638 | NF | NF | NF | NF | NF | NF |

| | | | | | | |
|---|---|---|---|---|---|---|
| Entomophthora muscae | NF | NF | NF | NF | NF | NF |
| Coemansia reversa NRRL 1564 | NF | NF | NF | NF | NF | NF |
| Capniomyces stellatus MIS-10-108 | NF | NF | NF | NF | NF | NF |
| Glomus cerebriforme | NF | NF | NF | NF | NF | NF |
| Rhizophagus irregularis DAOM 197198 | NF | NF | NF | NF | NF | NF |
| Mortierella antarctica | NF | NF | NF | NF | NF | NF |
| Mortiereinala alpina ATCC 32222 | NF | NF | NF | NF | NF | NF |
| Mucor circinelloides JCM 22480 | NF | NF | NF | NF | NF | NF |
| Umbelopsis isabellina B7317 | NF | NF | NF | NF | KAG2181921.1_hypothetical_protein_INT43_006846__Umbelopsis_isabellina | NF3e-29 |
| Rhizopus delemar RA 99-880 | NF | NF | NF | NF | NF | NF |
| Rhizopus oryzae 99-892 | NF | NF | NF | NF | NF | NF |
| Phycomyces blakesleeanus NRRL 1555 | NF | NF | NF | NF | NF | NF |
| Cryptococcus neoformans var. grubii H99 | XP_012051341.1 opsin 1 [Cryptococcus neoformans var. grubii H99] | 3.00E-27 | NF | NF | XP_012052525.1 bacteriophytochrome histidine kinase, XP_012052892.1 bacteriophytochrome histidine kinase, varian | 0.00E+00 |
| Ustilago maydis 521 | XP_011386259.1 hypothetical protein UMAG_00371 [Ustilago maydi, XP_011389026.1 hypothetical protein UMAG_02629 [Ustilago maydi | 1e-35, 3e-31 | NF | NF | XP_011391477.1 hypothetical protein UMAG_05732 | 0.00E+00 |
| Puccinia graminis f. sp. tritici CRL 75-36-700-3 | XP_003330211.1 hypothetical protein PGTG_11121 | 3.00E-18 | NF | NF | XP_003889564.1 hypothetical protein PGTG_21762 | 0.00E+00 |
| Coprinopsis cinerea okayama7#130 | NF | NF | NF | NF | XP_001835879.2 atypical/HisK protein kinase | 0.00E+00 |
| Aspergillus nidulans FGSC A4 | XP_660965.1 hypothetical protein AN3361.2 | 2.00E-64 | NF | NF | XP_682277.1 hypothetical protein AN9008.2 | 0.00E+00 |
| Trichoderma atroviride IMI 206040 | XP_013949009.1 hypothetical protein TRIATDRAFT_210598 | 1.00E-20 | NF | NF | XP_013942313.1 hypothetical protein TRIATDRAFT_319399 | 0.00E+00 |
| Neurospora crassa OR74A | AAD45253.1_opsin-1_[Neurospora_crassa] | 0.00E+00 | NF | NF | XP_960393.2 sensor histidine kinase/response regulator, XP_960050.2 phytochrome-like histidine kinase 2 | 0.00E+00 |
| Fusarium oxysporum NRRL 32931 | XP_031048691.1 uncharacterized protein FOYG_03723, XP_031030491.1 uncharacterized protein FOYG_15804 | 2E-118, 8e-31 | NF | NF | XP_031037841.1 uncharacterized protein FOYG_09928 | 0.00E+00 |
| Saitoella complicata NRRL Y-17804 | XP_019021587.1 family A G protein-coupled receptor-like protein | 5.00E-58 | NF | NF | XP_019024539.1 hypothetical protein SAICODRAFT_114514 | 0.00E+00 |
| Sclerotinia sclerotiorum 1980 UF-70 | XP_001597420.1 opsin-1 [Sclerotinia sclerotiorum 1980 UF-70] | 2.00E-93 | NF | NF | XP_001585628.1 hypothetical protein SS1G_13512, XP_001596430.1 hypothetical protein SS1G_02650, XP_001586250.1 hypothetical protein SS1G_12828 | 0.00E+00 |
| Saccharomyces cerevisiae S288C | NF | NF | NF | NF | NF | NF |
| Schizosaccharomyces pombe (ASM294v2) | NF | NF | NF | NF | NF | NF |

**Table S3. Presence and absence of CyclOp pathway elements (green), Carotenoid biosynthesis proteins (yellow) and photoreceptors (red) across 45 fungal proteomes, Related to Figure 3.** NF = Not Found.

References


S1. Avelar, G.M., Schumacher, R.I., Zaini, P.A., Leonard, G., Richards, T.A., and Gomes, S.L. (2014). A Rhodopsin-Guanylyl cyclase gene fusion functions in visual perception in a fungus. Curr. Biol. *24*, 1234–1240.

S2. Torruella, G., Grau-Bové, X., Moreira, D., Karpov, S.A., Burns, J.A., Sebé-Pedrós, A., Völcker, E., and López-García, P. (2018). Global transcriptome analysis of the aphelid Paraphelidium tribonemae supports the phagotrophic origin of fungi. Commun. Biol. *1*, 1–11.

S3. Chang, Y., Wang, S., Sekimoto, S., Aerts, A.L., Choi, C., Clum, A., LaButti, K.M., Lindquist, E.A., Ngan, C.Y., Ohm, R.A., et al. (2015). Phylogenomic analyses indicate that early fungi evolved digesting cell walls of algal ancestors of land plants. Genome Biol. Evol. *7*, 1590–1601.

S4. Mondo, S.J., Dannebaum, R.O., Kuo, R.C., Louie, K.B., Bewick, A.J., LaButti, K., Haridas, S., Kuo, A., Salamov, A., Ahrendt, S.R., et al. (2017). Widespread adenine N6-methylation of active genes in fungi. Nat. Genet. *49*, 964–968.

S5. van de Vossenberg, B.T.L.H., Warris, S., Nguyen, H.D.T., van Gent-Pelzer, M.P.E., Joly, D.L., van de Geest, H.C., Bonants, P.J.M., Smith, D.S., Lévesque, C.A., and van der Lee, T.A.J. (2019). Comparative genomics of chytrid fungi reveal insights into the obligate biotrophic and pathogenic lifestyle of Synchytrium endobioticum. Sci. Rep. *9*, 8672.

S6. Russ, C., Lang, B.F., Chen, Z., Gujja, S., Shea, T., Zeng, Q., Young, S., Cuomo, C.A., and Nusbaum, C. (2016). Genome sequence of Spizellomyces punctatus. Genome Announc. *4*, e00849-16.

S7. Joneson, S., Stajich, J.E., Shiu, S.H., and Rosenblum, E.B. (2011). Genomic transition to pathogenicity in chytrid fungi. PLoS Pathog. *7*.

S8. Galindo, L.J., López-García, P., Torruella, G., Karpov, S., and Moreira, D. (2021). Phylogenomics of a new fungal phylum reveals multiple waves of reductive evolution across Holomycota. Nat. Commun. *12*, 4973.

S9. Ribichich, K.F., Salem-Izacc, S.M., Georg, R.C., Vêncio, R.Z.N., Navarro, L.D., and Gomes, S.L. (2005). Gene discovery and expression profile analysis through sequencing of expressed sequence tags from different developmental stages of the chytridiomycete Blastocladiella emersonii. Eukaryot. Cell *4*, 455–464.

S10. Chang, Y., Rochon, D., Sekimoto, S., Wang, Y., Chovatia, M., Sandor, L., Salamov, A., Grigoriev, I. V, Stajich, J.E., and Spatafora, J.W. (2021). Genome-scale phylogenetic analyses confirm Olpidium as the closest living zoosporic fungus to the non-flagellated, terrestrial fungi. Sci. Rep. *11*, 3217.

S11. Chibucos, M.C., Soliman, S., Gebremariam, T., Lee, H., Daugherty, S., Orvis, J., Shetty, A.C., Crabtree, J., Hazen, T.H., Etienne, K.A., et al. (2016). An integrated genomic and transcriptomic survey of mucormycosis-causing fungi. Nat. Commun. *7*, 12218.

S12. De Fine Licht, H.H., Jensen, A.B., and Eilenberg, J. (2017). Comparative transcriptomics reveal host-specific nucleotide variation in entomophthoralean fungi. Mol. Ecol. *26*, 2092–2110.

S13. Wang, Y., White, M.M., and Moncalvo, J.-M. (2016). Draft genome sequence of Capniomyces stellatus, the obligate gut fungal symbiont of stonefly. Genome Announc. *4*, e00761-16.

S14. Morin, E., Miyauchi, S., San Clemente, H., Chen, E.C.H., Pelin, A., de la Providencia, I., Ndikumana, S., Beaudet, D., Hainaut, M., Drula, E., et al. (2019). Comparative genomics of Rhizophagus irregularis, R. cerebriforme, R. diaphanus and Gigaspora rosea highlights specific genetic features in Glomeromycotina. New Phytol. *222*, 1584–1598.

S15. Chen, E.C.H., Morin, E., Beaudet, D., Noel, J., Yildirir, G., Ndikumana, S., Charron, P., St-Onge, C., Giorgi, J., Krüger, M., et al. (2018). High intraspecific genome diversity in the model arbuscular mycorrhizal symbiont Rhizophagus irregularis. New Phytol. *220*, 1161–1171.

S16. Vandepol, N., Liber, J., Desirò, A., Na, H., Kennedy, M., Barry, K., Grigoriev, I. V, Miller, A.N., O'Donnell, K., Stajich, J.E., et al. (2020). Resolving the Mortierellaceae phylogeny through synthesis

of multi-gene phylogenetics and phylogenomics. Fungal Divers. *104*, 267–289.

S17. Wang, L., Chen, W., Feng, Y., Ren, Y., Gu, Z., Chen, H., Wang, H., Thomas, M.J., Zhang, B., Berquin, I.M., et al. (2011). Genome characterization of the oleaginous fungus Mortierella alpina. PLoS One *6*, 1–16.

S18. Navarro-Mendoza, M.I., Pérez-Arques, C., Panchal, S., Nicolás, F.E., Mondo, S.J., Ganguly, P., Pangilinan, J., Grigoriev, I. V, Heitman, J., Sanyal, K., et al. (2019). Early diverging fungus Mucor circinelloides lacks centromeric histone CENP-A and displays a mosaic of point and regional centromeres. Curr. Biol. *29*, 3791-3802.e6.

S19. Ma, L.-J., Ibrahim, A.S., Skory, C., Grabherr, M.G., Burger, G., Butler, M., Elias, M., Idnurm, A., Lang, B.F., Sone, T., et al. (2009). Genomic analysis of the basal lineage fungus Rhizopus oryzae reveals a whole-genome duplication. PLOS Genet. *5*, 1–11.

S20. Corrochano, L.M., Kuo, A., Marcet-Houben, M., Polaino, S., Salamov, A., Villalobos-Escobedo, J.M., Grimwood, J., Álvarez, M.I., Avalos, J., Bauer, D., et al. (2016). Expansion of signal transduction pathways in fungi by extensive genome duplication. Curr. Biol. *26*, 1577–1584.

S21. Janbon, G., Ormerod, K.L., Paulet, D., Byrnes III, E.J., Yadav, V., Chatterjee, G., Mullapudi, N., Hon, C.-C., Billmyre, R.B., Brunel, F., et al. (2014). Analysis of the genome and transcriptome of Cryptococcus neoformans var. grubii reveals complex RNA expression and microevolution leading to virulence attenuation. PLOS Genet. *10*, 1–26.

S22. Kämper, J., Kahmann, R., Bölker, M., Ma, L.-J., Brefort, T., Saville, B.J., Banuett, F., Kronstad, J.W., Gold, S.E., Müller, O., et al. (2006). Insights from the genome of the biotrophic fungal plant pathogen Ustilago maydis. Nature *444*, 97–101.

S23. Duplessis, S., Cuomo, C.A., Lin, Y.-C., Aerts, A., Tisserant, E., Veneault-Fourrey, C., Joly, D.L., Hacquard, S., Amselem, J., Cantarel, B.L., et al. (2011). Obligate biotrophy features unraveled by the genomic analysis of rust fungi. Proc. Natl. Acad. Sci. *108*, 9166–9171.

S24. Stajich, J.E., Wilke, S.K., Ahrén, D., Au, C.H., Birren, B.W., Borodovsky, M., Burns, C., Canbäck, B., Casselton, L.A., Cheng, C.K., et al. (2010). Insights into evolution of multicellular fungi from the assembled chromosomes of the mushroom Coprinopsis cinerea (Coprinus cinereus). Proc. Natl. Acad. Sci. U. S. A. *107*, 11889–11894.

S25. Galagan, J.E., Calvo, S.E., Cuomo, C., Ma, L.-J., Wortman, J.R., Batzoglou, S., Lee, S.-I., Baştürkmen, M., Spevak, C.C., Clutterbuck, J., et al. (2005). Sequencing of Aspergillus nidulans and comparative analysis with A. fumigatus and A. oryzae. Nature *438*, 1105–1115.

S26. Kubicek, C.P., Herrera-Estrella, A., Seidl-Seiboth, V., Martinez, D.A., Druzhinina, I.S., Thon, M., Zeilinger, S., Casas-Flores, S., Horwitz, B.A., Mukherjee, P.K., et al. (2011). Comparative genome sequence analysis underscores mycoparasitism as the ancestral life style of Trichoderma. Genome Biol. *12*, R40.

S27. Galagan, J.E., Calvo, S.E., Borkovich, K.A., Selker, E.U., Read, N.D., Jaffe, D., FitzHugh, W., Ma, L.-J., Smirnov, S., Purcell, S., et al. (2003). The genome sequence of the filamentous fungus Neurospora crassa. Nature *422*, 859–868.

S28. DeIulio, G.A., Guo, L., Zhang, Y., Goldberg, J.M., Kistler, H.C., Ma, L.-J., and Mitchell, A.P. (2018). Genome expansion in the Fusarium oxysporum species complex driven by accessory chromosomes. mSphere *3*, e00231-18.

S29. Riley, R., Haridas, S., Wolfe, K.H., Lopes, M.R., Hittinger, C.T., Göker, M., Salamov, A.A., Wisecaver, J.H., Long, T.M., Calvey, C.H., et al. (2016). Comparative genomics of biotechnologically important yeasts. Proc. Natl. Acad. Sci. *113*, 9882–9887.

S30. Amselem, J., Cuomo, C.A., van Kan, J.A.L., Viaud, M., Benito, E.P., Couloux, A., Coutinho, P.M., de Vries, R.P., Dyer, P.S., Fillinger, S., et al. (2011). Genomic analysis of the necrotrophic fungal pathogens Sclerotinia sclerotiorum and Botrytis cinerea. PLoS Genet. *7*, e1002230.

S31. Goffeau, A., Barrell, G., Bussey, H., Davis, R.W., Dujon, B., Feldmann, H., Galibert, F., Hoheisel, J.D.,

Jacq, C., Johnston, M., et al. (1996). Life with 6000 genes. Science (80-. ). *274*, 563–567.
S32. Wood, V., Gwilliam, R., Rajandream, M.-A., Lyne, M., Lyne, R., Stewart, A., Sgouros, J., Peat, N., Hayles, J., Baker, S., et al. (2002). The genome sequence of Schizosaccharomyces pombe. Nature *415*, 871–880.
S33. Reichle, R.E., and Fuller, M.S. (1967). The fine structure of Blastocladiella emersonii zoospores. Am. J. Bot. *54*, 81–92.
S34. Cantino, E.C., and Truesdell, L.C. (1970). Organization and fine structure of the side body and its lipid sac in the zoospore of Blastocladiella emersonii. Mycologia *62*, 548–567.
S35. Manier, J.-F. (1977). Cycle, ultrastructure d'une Catenaria (Phycomycètes, Blastocladiales) parasite de Crustacés Cyclopoides. Ann. Parasitol. Hum. Comp. *52*, 363–376.
S36. Fuller, M.S., and Olson, L.W. (1971). The zoospore of Allomyces. Microbiology *66*, 171–183.
S37. Karpov, S.A., Vishnyakov, A.E., Moreira, D., and López-García, P. (2019). The ultrastructure of Sanchytrium tribonematis (Sanchytriaceae, Fungi incertae sedis) confirms its close relationship to Amoeboradix. J. Eukaryot. Microbiol. *66*, 892–898.
S38. Karpov, S.A., López-García, P., Mamkaeva, M.A., Klimov, V.I., Vishnyakov, A.E., Tcvetkova, V.S., and Moreira, D. (2018). The chytrid-like parasites of algae Amoeboradix gromovi gen. et sp. nov. and Sanchytrium tribonematis belong to a new fungal lineage. Protist *169*, 122–140.
S39. Barr, D.J.S., and Hartmann, V.E. (1976). Zoospore ultrastructure of three Chytridium species and Rhizoclosmatium globosum. Can. J. Bot. *54*, 2000–2013.
S40. Letcher, P.M., Vélez, C.G., Barrantes, M.E., Powell, M.J., Churchill, P.F., and Wakefield, W.S. (2008). Ultrastructural and molecular analyses of Rhizophydiales (Chytridiomycota) isolates from North America and Argentina. Mycol. Res. *112*, 759–782.
S41. Longcore, J.E., Simmons, D.R., and Letcher, P.M. (2016). Synchytrium microbalum sp. nov. is a saprobic species in a lineage of parasites. Fungal Biol. *120*, 1156–1164.
S42. Mollicone, M.R.N., and Longcore, J.E. (1999). Zoospore Ultrastructure of Gonapodya polymorpha. Mycologia *91*, 727–734.
