# Supplementary material for: A light-sensing system in the common ancestor of the fungi
Source: Curr Biol. 2022 Jul 25;32(14):3146–3153.e3. doi: 10.1016/j.cub.2022.05.034 (PMC9616733; doi:10.1016/j.cub.2022.05.034)
Supplement: Document S2. Article plus supplemental information [file mmc2.pdf]

Report

Current Biology

A light-sensing system in the common ancestor of the fungi

Graphical abstract

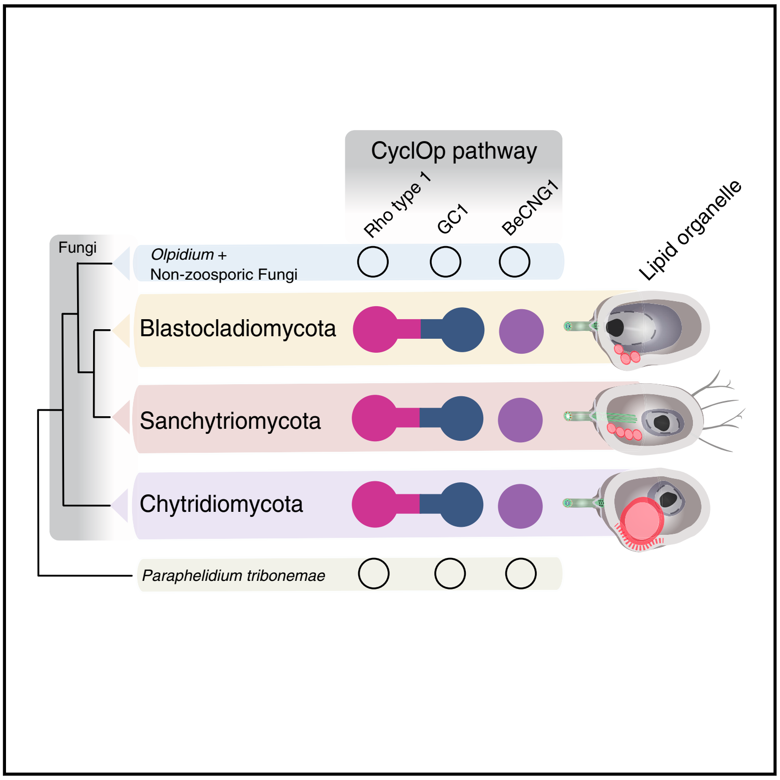

Authors

Luis Javier Galindo, David S. Milner, Suely Lopes Gomes, Thomas A. Richards

Correspondence

luis.galindogonzalez@zoo.ox.ac.uk (L.J.G.),
thomas.richards@zoo.ox.ac.uk (T.A.R.)

In brief

Galindo et al. demonstrate that a light response circuit, which controls swimming behavior, is present across diverse fungi. The circuit functions in coordination with a lipid-filled subcellular body. Divergent fungi possess similar cellular bodies. Phylogenies show that the ancestral fungus possessed this light-sensing system, along with other photoreceptors.

Highlights

- A wide diversity of flagellated fungi possess the CyclOp light response circuit
- The same fungi possess the subcellular equipment to build lipid-based eyespots
- The last common ancestor of fungi possessed the CyclOp eyespot system
- The ancestral fungus could see a rainbow of light wavelengths

Galindo et al., 2022, Current Biology *32*, 3146–3153
July 25, 2022 © 2022 The Author(s). Published by Elsevier Inc.
https://doi.org/10.1016/j.cub.2022.05.034

CellPress

CellPress
OPEN ACCESS

Current Biology

Report

A light-sensing system in the common ancestor of the fungi

Luis Javier Galindo,[1,3,*] David S. Milner,[1] Suely Lopes Gomes,[2] and Thomas A. Richards[1,4,5,*]
[1]Department of Zoology, University of Oxford, Oxford OX1 3SZ, UK
[2]Departamento de Bioquímica, Instituto de Química, Universidade de São Paulo, São Paulo 05508-000, Brazil
[3]Twitter: @Luisjagago
[4]Twitter: @AncestralState
[5]Lead contact
*Correspondence: luis.galindogonzalez@zoo.ox.ac.uk (L.J.G.), thomas.richards@zoo.ox.ac.uk (T.A.R.)
https://doi.org/10.1016/j.cub.2022.05.034

SUMMARY

Diverse light-sensing organs (i.e., eyes) have evolved across animals. Interestingly, several subcellular analogs have been found in eukaryotic microbes.[1] All of these systems have a common “recipe”: a light occluding or refractory surface juxtaposed to a membrane-layer enriched in type I rhodopsins.[1–4] In the fungi, several lineages have been shown to detect light using a diversity of non-homologous photo-responsive proteins.[5–7] However, these systems are not associated with an eyespot-like organelle with one exception found in the zoosporic fungus *Blastocladiella emersonii* (*Be*).[8] *Be* possesses both elements of this recipe: an eyespot composed of lipid-filled structures (often called the side-body complex [SBC]), co-localized with a membrane enriched with a gene-fusion protein composed of a type I (microbial) rhodopsin and guanylyl cyclase enzyme domain (CyclOp-fusion protein).[8,9] Here, we identify homologous pathway components in four Chytridiomycota orders (Chytridiales, Synchytriales, Rhizophydiales, and Monoblepharidiales). To further explore the architecture of the fungal zoospore and its lipid organelles, we reviewed electron microscopy data (e.g., the works of Barr and Hartmann[10] and Reichle and Fuller[11]) and performed fluorescence-microscopy imaging of four CyclOp-carrying zoosporic fungal species, showing the presence of a variety of candidate eyespot-cytoskeletal ultrastructure systems. We then assessed the presence of canonical photoreceptors across the fungi and inferred that the last common fungal ancestor was able to sense light across a range of wavelengths using a variety of systems, including blue-green-light detection. Our data imply, independently of how the fungal tree of life is rooted, that the apparatus for a CyclOp-organelle light perception system was an ancestral feature of the fungi.

RESULTS AND DISCUSSION

Presence of the CyclOp-organelle components across the fungi

The CyclOp protein[9] was first found to be in close spatial association with lipid-filled structures of the side-body complex (SBC) in *Blastocladiella emersonii* (*Be*),[8] so this system is named here as the “CyclOp organelle.” The system was shown to control the phototaxis behavior of *Be* zoospores by amending intracellular cyclic guanosine monophosphate (cGMP) levels, which trigger the function of a cyclic nucleotide-gated ion channel (BeCNG1)[12] and, therefore, regulates a green-light-sensing cascade, which results in flagellum beating.[8]

The proteins needed for light sensing in a CyclOp organelle (CyclOp and BeCNG1 proteins) have been found in the genomes of Blastocladiomycota and Sanchytriomycota[8,13] species. Using reciprocal BLAST and HMM protein sequence searches across the GenBank non-redundant databases[14] and 45 publicly available proteomes from fungi and one aphelid species (a protist closely related to the fungi[15]) (Table S1), we identified all the elements that are known to function in the CyclOp pathway (CyclOp and BeCNG1 proteins) in two chytrid orders: Chytridiales (*Chytriomyces confervae* CBS 675.73 and *Rhizoclosmatium globosum* JEL800) and Synchytriales (*Synchytrium microbalum* JEL517) (Figures 1A, S1A–S1C, and S2). We found multiple distinct CygclOp proteins in *C. confervae* and *R. globosum*, indicating that duplicated gene forms are present in Chytridiales. Furthermore, in the *Globomyces pollinis-pini* (Rhizophydiales) genome, we detected the CyclOp fusion-protein-encoding gene but did not detect a BeCNG1 channel-encoding gene. The BeCNG1 channel protein was also missing from the *Homolaphlyctis polyrhiza* genome assembly, and, furthermore, we detected two unfused domains (rhodopsin type I and GC1) putatively encoded as separate genes 547 nucleotides apart on this genome assembly, demonstrating that this gene has undergone gene fission, a phenomenon shown to be common in fungi.[16] Additionally, a member of one the earliest-diverging chytrid orders, Monoblepharidiales (*Gonapodya prolifera* JEL478), showed a partial CyclOp protein with a complete GC1 domain and a type I rhodopsin domain with a truncated N terminus (Figures 1A and S1A–S1C). The lack of a BeCNG1 protein and a truncated rhodopsin domain of the CyclOp protein indicates that this pathway is no longer functional

 © 2022 The Author(s). Published by Elsevier Inc.
This is an open access article under the CC BY license (http://creativecommons.org/licenses/by/4.0/).

CellPress
OPEN ACCESS

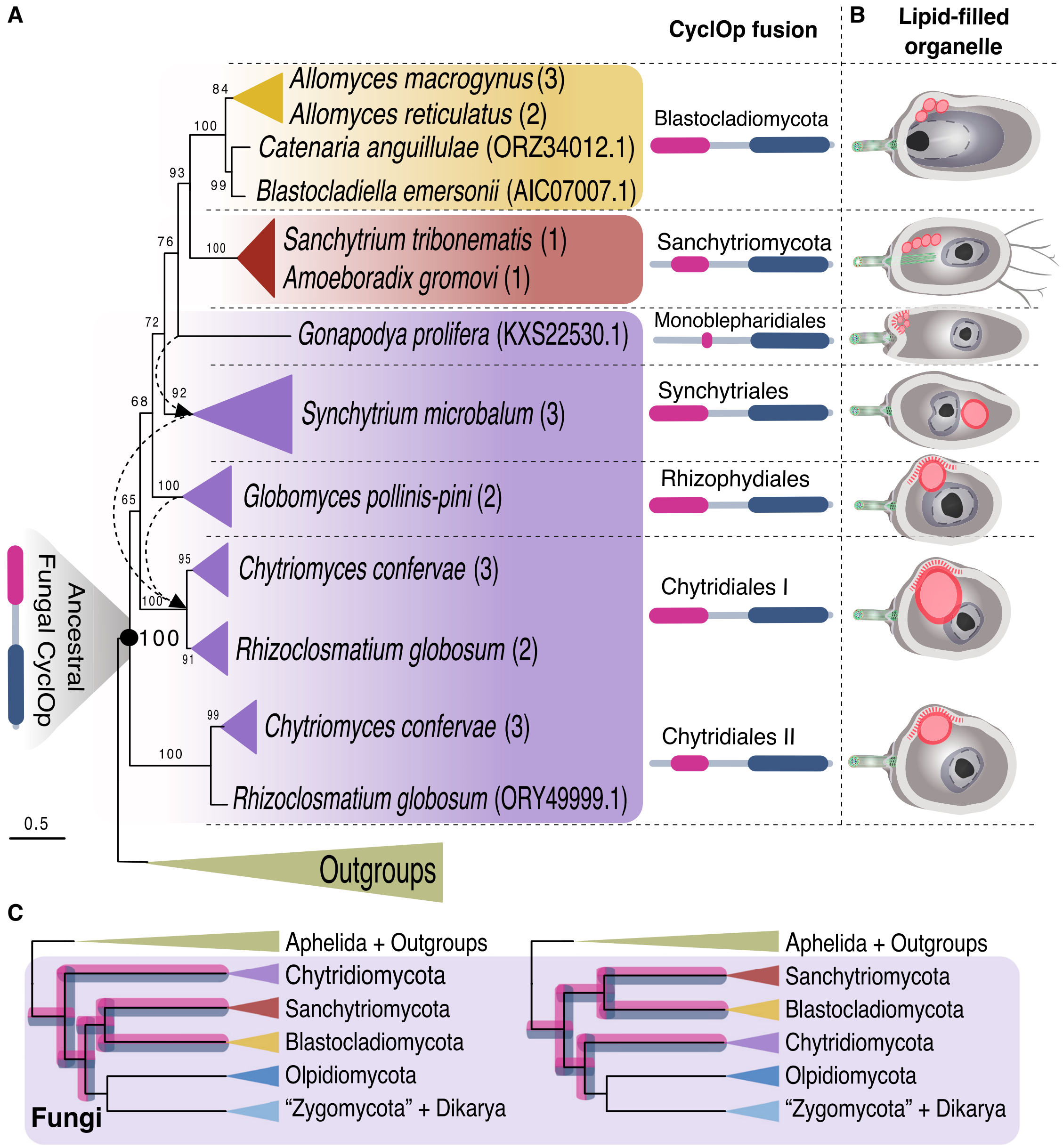

Figure 1. Evolution of the CyclOp system across the ancestral branches of the fungi

(A) Schematic representation of a maximum likelihood phylogenetic tree of the CyclOp protein gene fusion. The tree was reconstructed using 84 sequences with 3,095 amino acid positions and was inferred with IQ-TREE under the LG + F + I + G4 model with 1,000 ultrafast bootstrap replicates to assess statistical support. Dashed-lined arrows indicate the position of a given clade in the trimmed analysis (with only the type I rhodopsin domain; see supplemental information). Bootstrap support values are indicated before the nodes. Numbers within brackets on the fungal clade labels indicate the number of sequences sampled. The overall protein domain structure of the CyclOp was identified using homology-based 3D structure modeling[8] and is represented by rhodopsin (pink) and GC domains of the fusion (blue) (Figures S1–S3).

(B) Depiction of candidate CyclOp-organelle systems within the zoospore ultrastructure based on the review of a wide range of microscopy data. Next to the tree, we show schematic depictions of the presence of lipid-filled organelles (red circles) in the zoospores of fungi that possess the CyclOp gene. These depictions are based on a comprehensive review of published microscopy data (Table S2). Dashed red lines represent the presence of a rumposome (Table S2).

(C) Cladograms for the variant root hypotheses of the fungal tree and the evolution of the CyclOp system. Chytridiomycota-first (left) and Blastocladiomycota + Sanchytrids-first (right) hypotheses.[13] Pink-blue highlight on the branches indicates the presence of the CyclOp system across the tree.

See also Figures S1–S3 and Table S2.

or, alternatively, functions in a variant cGMP cascade in *Gonapodya*. In the Spizellomycetales species *Spizellomyces punctatus*, we found only the channel protein BeCNG1. We did not find the CyclOp system in the zoosporic aphelid *Paraphelidium tribonemae*, any of the Dikarya/Zygomycota fungi sampled, the frog pathogenic chytrid *Batrachochytrium dendrobatidis*, or in the

Olpidiomycota *Olpidium bornovanus*. These observations suggest that the CyclOp system originated in the ancestor of the fungi, has been lost or reduced in some chytrids, and was lost prior to the large-scale radiation of Olpidiomycota, Zygomycota, and Dikarya fungi that led to the evolution of the "terrestrial" fungal clade (Figure 1C).

Phylogenetic analysis demonstrated the monophyletic relationship of both the CyclOp and the BeCNG1 protein sequences from the Chytridiomycota, Blastocladiomycota, and Sanchytriomycota species with strong statistical support (trimmed and/or complete alignment analysis; Figures 1A, S1A–S1C, and S2). The taxonomic distribution of these homologous proteins and their phylogenetic placement support the idea of an ancestral CyclOp-mediated light-sensing pathway involving an eyespot-organelle in chytrids, blastoclads, and sanchytrids, and therefore, this pathway was present in the common ancestor of all the fungi (Figures 1A and 1C).

SBCs and other prominent lipid organelles have been recognized as subcellular systems in a diversity of zoosporic fungi (known as the microbody-lipid globule complex [MLC] in Chytridiomycota species and the SBC in Blastocladiomycota)[17] and are composed of microbodies, mitochondria, and several lipid globules enclosed by a membrane,[18] are associated with ribosomes,[10,17] and are usually located along one side of the nucleus. Homology of the SBC and MLC is not proven.[17] Yet our review of microscopy data identifies similar structures in several representatives of zoosporic fungal phyla[19] (Table S2). The function of this organelle has been linked to the provision of power for zoospore motility by continuous oxidation of lipids,[19] but we argue that these cell structures are also used for the formation of an eyespot-like structure. This idea is further supported by the fact that all these species possess different variations of a prominent lipid organelle, arranged in a similar fashion as the CyclOp organelle of *Be* (Figure 1B).[17] Indeed, ultrastructural studies of *Chytriomyces confervae* have already led to the suggestion that the system resembles an "eyespot"[10] in addition to an energy storage and provision organelle. Therefore, we propose that the lipid globules could act to refract or obscure the light from one side of the cell, allowing such systems to perceive the direction of a light source, implying that the function of the SBC and MLC organelle systems is linked to the photoresponse system. As discussed above, all these taxa also possess homologs of the CyclOp proteins shown to be critical for SBC-orchestrated phototaxis in *Be*.

Structural confocal fluorescence imaging of fungal zoospores

To gain further insights into the intracellular organization of fungal zoospores and to understand how the cytoskeleton is arranged relative to the lipid organelle, we conducted confocal microscopy with antibodies/fluorescent dyes that preferentially stain cytoskeleton elements or lipid droplets. We conducted microscopy imaging on zoospores of four species of zoosporic fungi in which the presence of all components of the CyclOp optogenetic circuit was confirmed. Two Blastocladiomycota cultures (*Be* ATCC 22665 and *Allomyces macrogynus* Australia_3) and two Chytridiomycota cultures (*Chytriomyces confervae* CBS 675.73 and *Rhizoclosmatium globosum* JEL800) were imaged. We note it has been reported that *A. macrogynus* zoospores do not show phototaxis behavior unlike other *Allomyces* species.[20,9,21] To stain the lipid components of zoospores, we used Nile red, and to stain the main components of the cytoskeleton, we used α-tubulin DM1A + Alexa Fluor 647 to stain tubulin and Alexa Fluor 488 Phalloidin to stain actin (Figure 2), both of which have previously been used to effectively study zoospore cytoskeletal systems in *B. dendrobatidis* and *R. globosum*.[22,23]

Our micrographs of *Be* (Figures 2A–2H; supplemental information in FigShare) show a large SBC located at the base of the flagellum and between the microtubular cytoskeleton of the zoospore and its cellular membrane. The SBC is composed of multiple lipid particles in close association (Figures 2A–2D). In *Allomyces*, the SBC is also located at the base of the flagellum and between the microtubule cytoskeleton and the cellular membrane but is composed of a lower number of lipid bodies (Figures 2I–2L; supplemental information in FigShare). *Allomyces* also demonstrates an agglomeration of multiple small apical lipid bodies in the anterior portion of the zoospores, located separately to the SBC.[24] This arrangement of lipid particles imbedded within the SBC matrix in *Blastocladiella* and in *Allomyces* has previously been reported.[11,24,25] However, the relative position of the SBC in relation to the cytoskeleton has not been well characterized. The cytoskeleton of both Blastocladiomycota species demonstrates a complex array of microtubules within the cell body and extending toward the flagellum (Figures 2B, 2F, and 2J). This array of microtubules has previously been observed as a Blastocladiomycota defining trait.[11,24,25] These microtubules run in nine sets of triplets (in the case of *Allomyces*) from the kinetosome to the apical portion of the cell body enclosing the nucleus and the nuclear cap, with the mitochondria and the SCB on the opposite side of the cell. Actin is also present in both Blastocladiomycota species, creating a non-structured network all over the zoospore cell body (Figures 2C, 2G, and 2K), including the flagellum, which is discussed further below.

Our images for the chytrids *C. confervae* (Figures 2M–2P; supplemental information in FigShare) and *R. globosum* (Figures 2Q–2T; supplemental information in FigShare) show similar cytoplasmic organization, consistent with their close taxonomic relationships (order Chytridiales). Both species show one large and prominent lipid body, which corresponds to the MLC located toward the lower-middle region of the cell, and adjacent to the zoospore cell surface, where we know, from previous transmission electron microscopy (TEM) studies, that the enigmatic structure (unique to Chytridiomycota) called the rumposome is present (Figure 1B, dashed red lines).[10,17,26] In these two chytrid species, microtubules are present only in the flagellum of the cells; however, previous TEM studies show that we can expect small microtubular bundles arising from the kinetosome and linking up directly to the rumposome. Higher-resolution imaging approaches would be necessary to observe these unique features that are potentially involved in phototaxis function.[10] In contrast to what we observed for the Blastocladiomycota zoospores, actin is the main cytoskeletal protein in the cell body of Chytridiomycota studied here and organizes itself into actin patches, which were clearly visible in almost all zoospores observed from both species (Figures 2O and 2S; supplemental information in FigShare). These actin patches have previously been observed in chytrids,[22,23] and they may correspond to what some authors refer to as fibrous areas identified from TEM analysis.[10] In the chytrid

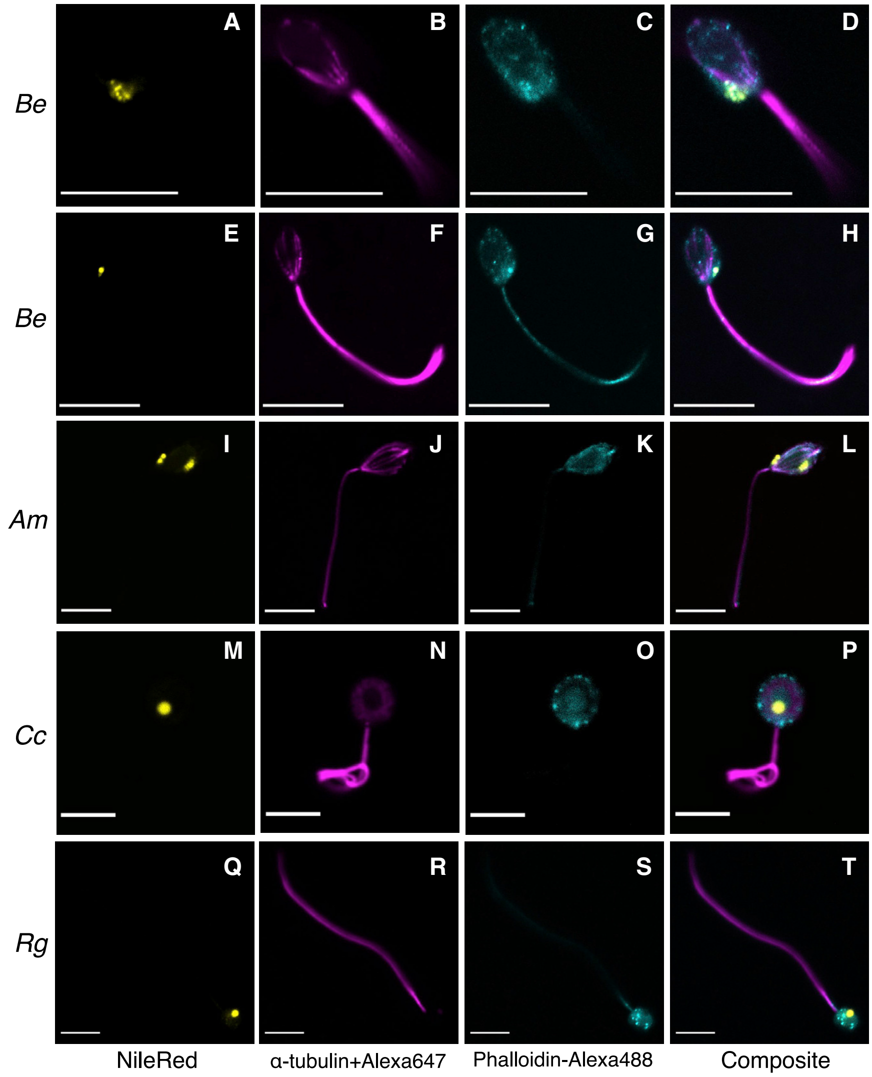

Figure 2. Confocal microscopic images of fluorescently stained fungal zoospores

Rows are organized according to the fungal species to which the zoospores correspond: (A–H) *Blastocladiella emersonii* ATCC 22665 (*Be*), (I–L) *Allomyces macrogynus* Australia_3 (*Am*), (M–P) *Chytriomyces confervae* CBS 675.73 (*Cc*), and (Q–T) *Rhizoclosmatium globosum* JEL800 (*Rg*). Columns are ordered according to the stains used: (A–Q) Nile red staining for lipid droplets, (B–R) α-tubulin DM1A + Alexa Fluor 647 fluorescence staining for microtubules, (C–S) Alexa Fluor 488 Phalloidin for actin, and (D–T) composite image of all fluorescence channels. Scale bars, 10 μm (A–L) and 5 μm (M–T). Two rows are shown for *Be*; the first row is shown to identify the details of the cell body, and the second row shows the extent of the flagellum and the presence of Phalloidin stain (actin) within the flagellum. Supplemental information in FigShare shows a diversity of replicate zoospores imaged using this same approach.

B.dendrobatidis, these actin patches were observed in a minority of the cells, and it has been argued that these are involved in endocytosis in sporangia. Thus, these structures can be interpreted as a signal that zoospores may have initiated their transition to the sporangial growth stage.[23] Some actin signal was also detected in the flagellum of a subset of the *R. globosum*, *B. emersonii*, and *A. macrogynus* zoospores (Figure 2; supplemental information in FigShare), a phenomenon previously observed in other eukaryotes.[27–29]

Presence of other photoreceptor proteins across the fungi

A range of photoreceptive proteins have been identified across the fungi with a diversity of different mechanisms of action and a variety of structures and light sensitivities.[5,30] To compare and contrast the evolution of the CyclOp system with other fungal photosystems, we performed protein searches of the previously mentioned 45 publicly available fungal/aphelid predicted proteomes (Table S1) in order to evaluate the presence of a diversity of candidate photoreceptors. These data allowed us to identify a picture of the evolution of these canonical photoreceptor systems across the fungi. This analysis includes searches for the following: (1) the conserved fungal photoreceptor proteins for blue light, the white-collar complex (WCC; White-Collar protein 1 [WC-1], White-Collar protein 2 [WC-2], and Vivid [VVD]); (2) the cryptochrome (CRY);[5,30] (3) the green-light sensitive type I and II rhodopsins (the opsin NOP-1 and the type II opsin-like GPCR);[31–33] and (4) the red-light-responsive phytochromes PHY-1 and PHY-2[5,34,35] (Figure 3; Table S3, which is also available at FigShare: https://doi.org/10.6084/m9.FigShare.19182086.v4).

Our results show that blue-light sensing complex and variant photoreceptors can be traced back to the ancestor of the fungi, as they are present in all major fungal groups from zoosporic to dikaryan species. In the case of the WCC, WC-1 and WC-2 are found to be co-present in representatives of every major fungal group, consistent with the co-function of this complex, which can also be traced back to the last common ancestor of the fungi. However, the white-collar proteins are not present in the aphelid *Paraphelidium tribonemae* proteome. The VVD protein, which interacts with the WCC,[5,30] evolved in the ancestor of Ascomycota. By contrast, the blue receptor cryptochrome CRY protein is found throughout the fungi and is present in the aphelid *P. tribonemae*, so it must have evolved prior to the rise of the last common ancestor of the fungi (Figure 3B).

Aside from the rhodopsin present in the CyclOp-fusion protein, other distinct/unfused single-domain rhodopsin-protein-encoding genes have been shown to be present in fungal taxa,[7] with the first published demonstration of the participation of a rhodopsin protein in phototaxis in the Blastocladiomycota *Allomyces reticulatus*.[20] Opsins can be generally classified into two types, which share little sequence similarity: type I are typically found in prokaryotes and type II are primarily found in the

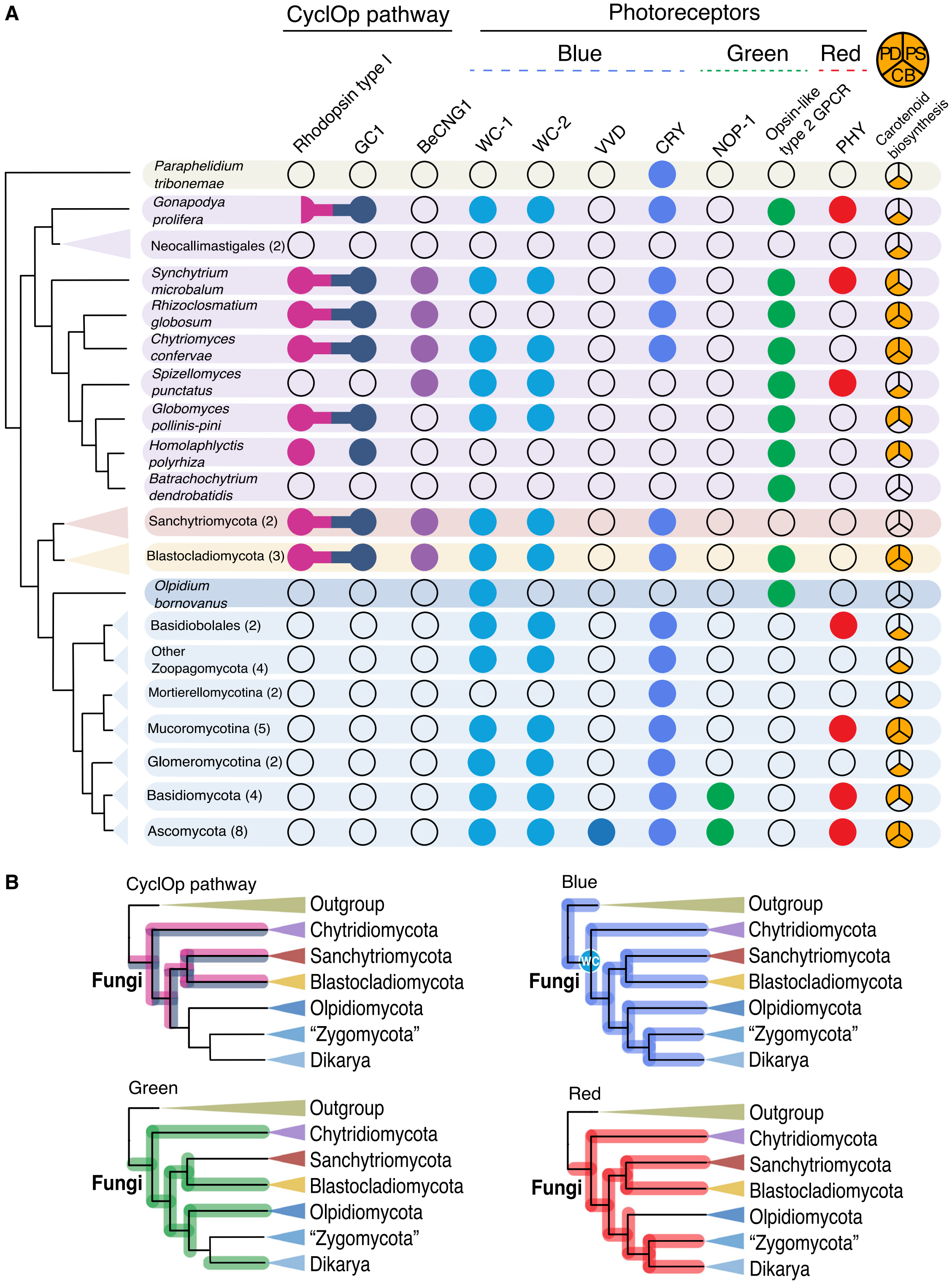

Figure 3. CyclOp pathway and photoreceptors across the fungal tree of life

(A) Coulson plot of the presence/absence of the CyclOp pathway and other photoreceptor systems across the fungi. Colored dots indicate the presence of homologs of a given protein of the CyclOp pathway or specific photoreceptors. The type I rhodopsin and the GC1 domain of the CyclOp fusion are shown in pink and blue, respectively, and the BeCNG1 channel protein in purple. Protein domains that are encoded by a gene fusion are indicated by a connection between the dots, and an incomplete protein domain is represented by a half dot. Dots for photoreceptors are indicated in blue for blue light (WC-1 and its partner WC-2, VVD, and the cryptochrome CRY), green for green light (the opsin NOP-1 and the opsin-like type II GPCR), and red for red light (the phytochromes PHY-1 and PHY-2). The

(legend continued on next page)

Metazoa.[31,36] Our results show that green-light sensing in fungi through the type I NOP-1 opsin has a limited distribution but can be traced back to the origin of dikaryan fungi (Figure 3). However, a type II rhodopsin was also found using structural comparisons in the zoosporic fungi *Spizellomyces punctatus* and later identified in other zoosporic relatives, e.g., *Allomyces macrogynus*, *Batrachochytrium dendrobatidis*, and *Homolaphlyctis polyrhiza*.[31] We searched for this type II rhodopsin-like GPCR gene and found it in a range of zoosporic fungi but not in the sanchytrids and the Neocallimastigales (Figure 3). Thus, it seems that the type II rhodopsin was present in the fungal ancestor and was then lost in multiple taxa (e.g., Zoopagomycota, Mucoromycota, and Dikarya). The type I NOP-1 opsin was then acquired by dikaryan fungi, fulfilling green-light-sensing functions.[32,33,37] The early evolution of blue- and green-light receptors in the fungi fits with its essential role in the regulation of circadian clocks and the predominance of blue-green-light wavelengths, which penetrate the aquatic depths,[38–40] where the fungi first diversified.[41,42]

Lastly, red-light sensing in fungi is performed by the phytochromes PHY-1 and PHY-2.[5,34,35,43] Our results show that red-light sensing can also be traced back to the dawn of the fungi since the genes encoding these proteins are present across the major fungal clades. However, it seems that the phytochromes have been lost several times within the fungi, including in Sanchytriomycota, Blastocladiomycota, Moretierellomycotina, and Glomeromycotina (Figure 3). It is interesting to highlight the absence of photoreceptor-encoding genes (including CyclOp) in Neocallimastigales, which likely reflects adaptation to a lifestyle within the dark digestive tracts of mammalian herbivores,[44,45] whereas the loss of the CyclOp system in *Spizellomyces* may reflect their adaptation to soil environments.[46,47]

Rhodopsin function depends on the presence of a retinal chromophore co-factor.[8,48] We searched the sampled genomes for the carotenoid (β-carotene) biosynthesis pathway enzyme genes necessary for the production of retinal. We found all three enzyme-encoding genes involved in this pathway (bifunctional lycopene cyclase/phytoene synthase, phytoene dehydrogenase, and carotenoid oxygenase)[8] in the proteomes of the two Chytridiales species—all but the bifunctional lycopene cyclase/phytoene synthase in the proteome of *S. microbalum* and all but the carotenoid oxygenase in Rhizophydiales (except *Batrachochytrium*). In the case of Neocallimastigales, Spizellomycetales, and *G. prolifera*, we could find only one of the three enzymes (carotenoid oxygenase) (Figure 3; Table S3).

Conclusions

Our results show a wide taxonomic distribution of the CyclOp system across zoosporic fungi. These data demonstrate that the ancestor of the fungi possessed the key elements of the CyclOp system, independent of the fungal root hypothesis favored (Figure 1C), and therefore may have been able to sense light through this pathway. The exploration of the intracellular organization of fungal zoospores using fluorescence microscopy demonstrates that a diversity of fungi produce zoospores containing lipid organelles, which are also likely to be associated with eyespot function. This result is again consistent with the idea that the last common ancestor of the fungi possessed the cellular equipment to build the cytological platform to support the function of the CyclOp optogenetic circuit. These results build upon a body of work demonstrating that early fungal forms sensed and responded to light in numerous different ways. Indeed, our data indicate that the ancestor of the fungi may have been able to sense blue, green, and red light, due to the presence of conserved canonical fungal photoreceptors. Such a result is also consistent with the hypothesis that early fungal forms were co-habiting and trophically interacting with photosynthetic forms since light sensing would allow early fungi to navigate toward environments inhabited by photosynthetic organisms.[19,41]

STAR★METHODS

Detailed methods are provided in the online version of this paper and include the following:

- KEY RESOURCES TABLE
- RESOURCE AVAILABILITY
  - Lead contact
  - Materials availability
  - Data and code availability
- EXPERIMENTAL MODEL AND SUBJECT DETAILS
- METHOD DETAILS
  - CyclOp pathway gene database searches and phylogenetic analyses
  - Fungal photoreceptors searches across proteomes
  - Synthesis of ultrastructure, protein architecture and evolutionary data
  - Fluorescence microscopy
- QUANTIFICATION AND STATISTICAL ANALYSIS

SUPPLEMENTAL INFORMATION

Supplemental information can be found online at https://doi.org/10.1016/j.cub.2022.05.034.

ACKNOWLEDGMENTS

This work was funded by the Horizon 2020 research and innovation programme under the European Marie Skłodowska-Curie Individual Fellowship H2020-MSCA-IF-2020 (grant agreement no. 101022101—FungEye). T.A.R. is supported by a Royal Society URF (URF/R/191005). S.L.G. was partially supported by the Conselho Nacional de Desenvolvimento Científico e Tecnológico (CNPq). We gratefully acknowledge the Micron Advanced Bioimaging Facility (supported by Wellcome Strategic Awards 091911/B/10/Z and 107457/Z/15/Z), particularly Deidre Kavanagh, for their support and assistance in this work. We would also like to thank Manuel Giménez-Andrés for his advice on microscope image processing. This paper was inspired by an exciting journey into pioneering work using the EM analysis of fungal

orange pie charts represent the presence/absence of the three enzymes involved in carotenoid biosynthesis: phytoene dehydrogenase (PD), phytoene synthase (PS), and carotenoid oxygenase (CB). Numbers between brackets on the fungal clades indicate the number of species proteomes analyzed. Table S3 (also available in FigShare) contains the data used to construct the Coulson plot.

(B) Cladograms indicating the evolutionary path of the diversification of the photoreceptors of fungi. The CyclOp pathway (highlighted in pink and blue) and photoreceptors for green (highlighted in green), blue (highlighted in blue), and red (highlighted in red) are shown. WC, white-collar complex. See also Table S3.

zoospores, started over 50 years ago. We thank these pioneers, chief among them Prof. Martha Powell.

AUTHOR CONTRIBUTIONS

L.J.G. and D.S.M. conducted the experiments. L.J.G. and T.A.R designed the experiments and wrote the paper. S.L.G. provided the reagents. All authors edited and added to the writing process.

DECLARATION OF INTERESTS

The authors declare no competing interests.

Received: November 21, 2021
Revised: April 4, 2022
Accepted: May 12, 2022
Published: June 7, 2022

REFERENCES

1. Richards, T.A., and Gomes, S.L. (2015). Protistology: how to build a microbial eye. Nature *523*, 166–167.
2. Schmidt, M., Gessner, G., Luff, M., Heiland, I., Wagner, V., Kaminski, M., Geimer, S., Eitzinger, N., Reissenweber, T., Voytsekh, O., et al. (2006). Proteomic analysis of the eyespot of *Chlamydomonas reinhardtii* provides novel insights into its components and tactic movements. Plant Cell *18*, 1908–1930.
3. Gavelis, G.S., Hayakawa, S., White, R.A., Gojobori, T., Suttle, C.A., Keeling, P.J., and Leander, B.S. (2015). Eye-like ocelloids are built from different endosymbiotically acquired components. Nature *523*, 204–207.
4. Sineshchekov, O.A., Govorunova, E.G., Jung, K.H., Zauner, S., Maier, U.G., and Spudich, J.L. (2005). Rhodopsin-mediated photoreception in cryptophyte flagellates. Biophys. J. *89*, 4310–4319.
5. Corrochano, L.M. (2019). Light in the fungal world: from photoreception to gene transcription and beyond. Annu. Rev. Genet. *53*, 149–170.
6. Yu, Z., and Fischer, R. (2019). Light sensing and responses in fungi. Nat. Rev. Microbiol. *17*, 25–36.
7. Brown, L.S. (2004). Fungal rhodopsins and opsin-related proteins: eukaryotic homologues of bacteriorhodopsin with unknown functions. Photochem. Photobiol. Sci. *3*, 555–565.
8. Avelar, G.M., Schumacher, R.I., Zaini, P.A., Leonard, G., Richards, T.A., and Gomes, S.L. (2014). A rhodopsin-guanylyl cyclase gene fusion functions in visual perception in a fungus. Curr. Biol. *24*, 1234–1240.
9. Gao, S., Nagpal, J., Schneider, M.W., Kozjak-Pavlovic, V., Nagel, G., and Gottschalk, A. (2015). Optogenetic manipulation of cGMP in cells and animals by the tightly light-regulated guanylyl-cyclase opsin CyclOp. Nat. Commun. *6*, 8046.
10. Barr, D.J.S., and Hartmann, V.E. (1976). Zoospore ultrastructure of three *Chytridium* species and *Rhizoclosmatium globosum*. Can. J. Bot. *54*, 2000–2013.
11. Reichle, R.E., and Fuller, M.S. (1967). The fine structure of *Blastocladiella emersonii* zoospores. Am. J. Bot. *54*, 81–92.
12. Avelar, G.M., Glaser, T., Leonard, G., Richards, T.A., Ulrich, H., and Gomes, S.L. (2015). A cyclic GMP-dependent K+ channel in the blastocladiomycete fungus *Blastocladiella emersonii*. Eukaryot. Cell *14*, 958–963.
13. Galindo, L.J., López-García, P., Torruella, G., Karpov, S., and Moreira, D. (2021). Phylogenomics of a new fungal phylum reveals multiple waves of reductive evolution across Holomycota. Nat. Commun. *12*, 4973.
14. Pruitt, K.D., Tatusova, T., and Maglott, D.R. (2007). NCBI reference sequences (RefSeq): a curated non-redundant sequence database of genomes, transcripts and proteins. Nucleic Acids Res. *35*, D61–D65.
15. Torruella, G., Grau-Bové, X., Moreira, D., Karpov, S.A., Burns, J.A., Sebé-Pedrós, A., Völcker, E., and López-García, P. (2018). Global transcriptome analysis of the aphelid *Paraphelidium tribonemae* supports the phagotrophic origin of fungi. Commun. Biol. *1*, 231.
16. Leonard, G., and Richards, T.A. (2012). Genome-scale comparative analysis of gene fusions, gene fissions, and the fungal tree of life. Proc. Natl. Acad. Sci. USA *109*, 21402–21407.
17. Powell, M.J. (1978). Phylogenetic implications of the microbody-lipid globule complex in zoosporic fungi. Biosystems *10*, 167–180.
18. James, T.Y., Porter, T.M., and Martin, W.W. (2014). Blastocladiomycota. In Systematics and Evolution, D.J. McLaughlin, and J.W. Spatafora, eds. (Springer), pp. 177–207.
19. Powell, M.J. (2017). Chytridiomycota. In Handbook of the Protists, second edition (Springer), pp. 1523–1558.
20. Saranak, J., and Foster, K.W. (1997). Rhodopsin guides fungal phototaxis. Nature *387*, 465–466.
21. Swafford, A.J.M., and Oakley, T.H. (2018). Multimodal sensorimotor system in unicellular zoospores of a fungus. J. Exp. Biol. *221*, jeb163196.
22. Venard, C.M., Vasudevan, K.K., and Stearns, T. (2020). Cilium axoneme internalization and degradation in chytrid fungi. Cytoskeleton (Hoboken) *77*, 365–378.
23. Prostak, S.M., Robinson, K.A., Titus, M.A., and Fritz-Laylin, L.K. (2021). The actin networks of chytrid fungi reveal evolutionary loss of cytoskeletal complexity in the fungal kingdom. Curr. Biol. *31*, 1192–1205.e6.
24. Fuller, M.S., and Olson, L.W. (1971). The zoospore of *Allomyces*. Microbiology *66*, 171–183.
25. Cantino, E.C., and Truesdell, L.C. (1970). Organization and fine structure of the side body and its lipid sac in the zoospore of *Blastocladiella emersonii*. Mycologia *62*, 548–567.
26. Powell, M.J. (1983). Localization of antimonate-mediated precipitates of cations in zoospores of *Chytriomyces hyalinus*. Exp. Mycol. *7*, 266–277.
27. Watanabe, Y., Hayashi, M., Yagi, T., and Kamiya, R. (2004). Turnover of actin in *Chlamydomonas* flagella detected by fluorescence recovery after photobleaching (FRAP). Cell Struct. Funct. *29*, 67–72.
28. Kiesel, P., Alvarez Viar, G., Tsoy, N., Maraspini, R., Gorilak, P., Varga, V., Honigmann, A., and Pigino, G. (2020). The molecular structure of mammalian primary cilia revealed by cryo-electron tomography. Nat. Struct. Mol. Biol. *27*, 1115–1124.
29. Piperno, G., and Luck, D.J. (1979). An actin-like protein is a component of axonemes from *Chlamydomonas* flagella. J. Biol. Chem. *254*, 2187–2190.
30. Idnurm, A., Verma, S., and Corrochano, L.M. (2010). A glimpse into the basis of vision in the kingdom *Mycota*. Fungal Genet. Biol. *47*, 881–892.
31. Ahrendt, S.R., Medina, E.M., Chang, C.-E.A., and Stajich, J.E. (2017). Exploring the binding properties and structural stability of an opsin in the chytrid *Spizellomyces punctatus* using comparative and molecular modeling. PeerJ *5*, e3206.
32. Bieszke, J.A., Spudich, E.N., Scott, K.L., Borkovich, K.A., and Spudich, J.L. (1999). A eukaryotic protein, NOP-1, binds retinal to form an archaeal rhodopsin-like photochemically reactive pigment. Biochemistry *38*, 14138–14145.
33. Wang, Z., Wang, J., Li, N., Li, J., Trail, F., Dunlap, J.C., and Townsend, J.P. (2018). Light sensing by opsins and fungal ecology: NOP-1 modulates entry into sexual reproduction in response to environmental cues. Mol. Ecol. *27*, 216–232.
34. Blumenstein, A., Vienken, K., Tasler, R., Purschwitz, J., Veith, D., Frankenberg-Dinkel, N., and Fischer, R. (2005). The *Aspergillus nidulans* phytochrome FphA represses sexual development in red light. Curr. Biol. *15*, 1833–1838.
35. Brandt, S., von Stetten, D., Günther, M., Hildebrandt, P., and Frankenberg-Dinkel, N. (2008). The fungal phytochrome FphA from *Aspergillus nidulans*. J. Biol. Chem. *283*, 34605–34614.
36. Shichida, Y., and Matsuyama, T. (2009). Evolution of opsins and phototransduction. Philos. Trans. R. Soc. Lond. B Biol. Sci. *364*, 2881–2895.
37. Bieszke, J.A., Braun, E.L., Bean, L.E., Kang, S., Natvig, D.O., and Borkovich, K.A. (1999). The nop-1 gene of *Neurospora crassa* encodes a seven transmembrane helix retinal-binding protein homologous to archaeal rhodopsins. Proc. Natl. Acad. Sci. USA *96*, 8034–8039.

CellPress
OPEN ACCESS

38. Williams, D.L. (2016). Light and the evolution of vision. Eye (Lond) *30*, 173–178.
39. Montenegro-Montero, A., Canessa, P., and Larrondo, L.F. (2015). Around the fungal clock: recent advances in the molecular study of circadian clocks in *Neurospora* and other fungi. Adv. Genet. *92*, 107–184.
40. Liu, Y., and Bell-Pedersen, D. (2006). Circadian rhythms in *Neurospora crassa* and other filamentous fungi. Eukaryot. Cell *5*, 1184–1193.
41. Lutzoni, F., Nowak, M.D., Alfaro, M.E., Reeb, V., Miadlikowska, J., Krug, M., Arnold, A.E., Lewis, L.A., Swofford, D.L., Hibbett, D., et al. (2018). Contemporaneous radiations of fungi and plants linked to symbiosis. Nat. Commun. *9*, 5451.
42. Naranjo-Ortiz, M.A., and Gabaldón, T. (2019). Fungal evolution: major ecological adaptations and evolutionary transitions. Biol. Rev. Camb. Philos. Soc. *94*, 1443–1476.
43. Wang, Z., Li, N., Li, J., Dunlap, J.C., Trail, F., and Townsend, J.P. (2016). The fast-evolving phy-2 gene modulates sexual development in response to light in the model fungus *Neurospora crassa*. mBio *7*, e02148.
44. Powell, M.J. (2017). Blastocladiomycota. In Handbook of the Protists, second edition (Springer), pp. 1497–1521.
45. Gruninger, R.J., Puniya, A.K., Callaghan, T.M., Edwards, J.E., Youssef, N., Dagar, S.S., Fliegerova, K., Griffith, G.W., Forster, R., Tsang, A., et al. (2014). Anaerobic fungi (phylum *Neocallimastigomycota*): advances in understanding their taxonomy, life cycle, ecology, role and biotechnological potential. FEMS Microbiol. Ecol. *90*, 1–17.
46. Paulitz, T.C., and Menge, J.A. (1984). Is *Spizellomyces punctatum* a parasite or saprophyte of vesicular-arbuscular mycorrhizal fungi? Mycologia *76*, 99–107.
47. Lozupone, C.A., and Klein, D.A. (2002). Molecular and cultural assessment of chytrid and *Spizellomyces* populations in grassland soils. Mycologia *94*, 411–420.
48. Needham, D.M., Yoshizawa, S., Hosaka, T., Poirier, C., Choi, C.J., Hehenberger, E., Irwin, N.A.T., Wilken, S., Yung, C.M., Bachy, C., et al. (2019). A distinct lineage of giant viruses brings a rhodopsin photosystem to unicellular marine predators. Proc. Natl. Acad. Sci. USA *116*, 20574–20583.
49. Schindelin, J., Arganda-Carreras, I., Frise, E., Kaynig, V., Longair, M., Pietzsch, T., Preibisch, S., Rueden, C., Saalfeld, S., Schmid, B., et al. (2012). Fiji: an open-source platform for biological-image analysis. Nat. Methods *9*, 676–682.
50. Altschul, S.F., Gish, W., Miller, W., Myers, E.W., and Lipman, D.J. (1990). Basic local alignment search tool. J. Mol. Biol. *215*, 403–410.
51. Finn, R.D., Clements, J., and Eddy, S.R. (2011). HMMER web server: interactive sequence similarity searching. Nucleic Acids Res. *39*, W29–W37.
52. Katoh, K., Misawa, K., Kuma, K.I., and Miyata, T. (2002). MAFFT: a novel method for rapid multiple sequence alignment based on fast Fourier transform. Nucleic Acids Res. *30*, 3059–3066.
53. Capella-Gutiérrez, S., Silla-Martínez, J.M., and Gabaldón, T. (2009). trimAl: a tool for automated alignment trimming in large-scale phylogenetic analyses. Bioinformatics *25*, 1972–1973.
54. Nguyen, L.T., Schmidt, H.A., Von Haeseler, A., and Minh, B.Q. (2015). IQ-TREE: a fast and effective stochastic algorithm for estimating maximum-likelihood phylogenies. Mol. Biol. Evol. *32*, 268–274.
55. Rambaut, A. (2016). FigTree v1.4.3. http://tree.bio.ed.ac.uk/software/figtree/.
56. Clark, K., Karsch-Mizrachi, I., Lipman, D.J., Ostell, J., and Sayers, E.W. (2016). GenBank. Nucleic Acids Res. *44*, D67–D72.
57. Mollicone, M.R.N., and Longcore, J.E. (1999). Zoospore ultrastructure of *Gonapodya polymorpha*. Mycologia *91*, 727–734.
58. Manier, J.-F. (1977). Cycle, ultrastructure d'une *Catenaria* (Phycomycètes, Blastocladiales) parasite de Crustacés Cyclopoides. Ann. Parasitol. Hum. Comp. *52*, 363–376.
59. Karpov, S.A., Vishnyakov, A.E., Moreira, D., and López-García, P. (2019). The ultrastructure of *Sanchytrium tribonematis* (Sanchytriaceae, Fungi *incertae sedis*) confirms its close relationship to *Amoeboradix*. J. Eukaryot. Microbiol. *66*, 892–898.
60. Karpov, S.A., López-García, P., Mamkaeva, M.A., Klimov, V.I., Vishnyakov, A.E., Tcvetkova, V.S., and Moreira, D. (2018). The chytrid-like parasites of algae *Amoeboradix gromovi* gen. et sp. nov. and *Sanchytrium tribonematis* belong to a new fungal lineage. Protist *169*, 122–140.
61. Longcore, J.E., Simmons, D.R., and Letcher, P.M. (2016). *Synchytrium microbalum* sp. nov. is a saprobic species in a lineage of parasites. Fungal Biol. *120*, 1156–1164.
62. Letcher, P.M., Vélez, C.G., Barrantes, M.E., Powell, M.J., Churchill, P.F., and Wakefield, W.S. (2008). Ultrastructural and molecular analyses of *Rhizophydiales* (*Chytridiomycota*) isolates from North America and Argentina. Mycol. Res. *112*, 759–782.

STAR★METHODS

KEY RESOURCES TABLE

REAGENT or RESOURCE	SOURCE	IDENTIFIER
Antibodies		
α-tubulin DM1A	Sigma-Aldrich	Cat#T6199; RRID: AB_477583
Alexa Fluor 647 Goat anti-mouse IgG1 antibody	Thermo Fisher	Cat#A-21240; RRID: AB_2535809
Alexa Fluor 488 Phalloidin	Invitrogen	Cat#A12379; RRID: AB_2759222
Stains		
Nile Red	Thermo Fisher	Cat#N1142
Reagents		
Citifluor AF2 Antifadent Mountant Solution	Electron Microscopy Sciences	AF2
Deposited data		
CyclOp pathway and photoreceptors in fungi, alignment datasets and trees for the CyclOp and BeCNG1 proteins, trees and aligned datasets used for phylogenetic reconstruction of the CyclOp pathway in the Fungi, table of photoreceptors and retinal synthesis genes.	This study	https://doi.org/10.6084/m9.figshare.19182086.v4
Experimental models: Organisms/strains		
Blastocladiella emersonii ATCC 22665	ATCC	ATCC 22665
Chytridium confervae CBS 675.73	Westerdijk Fungal Biodiversity Institute	CBS 675.73
Rhizoclosmatium globosum JEL800	CZEUM	JEL800
Allomyces macrogynus Australia_3	CZEUM	Australia_3
Software and algorithms		
Fiji 2.3.1	Schindelin et al. [49]	https://fiji.sc/; RRID: SCR_002285
Black Zen software	ZEN Digital Imaging for Light Microscopy	https://www.zeiss.com/microscopy/int/products/microscope-software/zen.html; RRID: SCR_018163
BLAST	Altschul et al. [50]	https://blast.ncbi.nlm.nih.gov/Blast.cgi; RRID: SCR_004870
HMMER v3.3.2	Finn et al. [51]	https://www.ebi.ac.uk/Tools/hmmer/search/hmmscan; RRID: SCR_005305
MAFFT version 7	Katoh et al. [52]	https://mafft.cbrc.jp/alignment/server/; RRID: SCR_011811
TrimAl v1.2	Capella-Gutiérrez et al.[53]	http://trimal.cgenomics.org/; RRID: SCR_017334
IQ-TREE version 1.6.12	Nguyen et al. [54]	http://www.iqtree.org/; RRID: SCR_017254
FigTree v1.4.3	Rambaut [55]	http://tree.bio.ed.ac.uk/software/figtree/; RRID: SCR_008515

RESOURCE AVAILABILITY

Lead contact

Further information and requests for resources and reagents should be directed to and will be fulfilled by the Lead Contact, Thomas A. Richards (thomas.richards@zoo.ox.ac.uk).

Materials availability

This study did not generate new unique reagents.

Data and code availability

All data are available in the figures, tables, and data files associated with this manuscript. This study did not result in any unique code. Any additional information required to reanalyze the data reported in this work paper is available from the Lead Contact upon request

EXPERIMENTAL MODEL AND SUBJECT DETAILS

Cultures of *Blastocladiella emersonii* ATCC 22665 (ATCC; American Type Culture Collection) and *Chytriomyces confervae* CBS 675.73 (Westerdijk Fungal Biodiversity Institute) were vegetatively grown in Nunc EasYFlask 25cm^2 culture flasks (ThermoFisher) filled with 25 ml of PYG liquid media (0.13% w/v peptone, 0.13% w/v yeast extract, 0.3% w/v glucose). *Rhizoclosmatium globosum* JEL800 (CZEUM; Collection of Zoosporic Eufungi at University of Michigan) vegetative cells were grown on PYG agar plates (0.13% w/v peptone, 0.13% w/v yeast extract, 0.3% w/v glucose, and 1.5% w/v agar). *Allomyces macrogynus* Australia_3 (CZEUM) was grown in 25cm^2 culture flasks filled with 25 ml of Emerson YpSs/4 liquid media (0.1% w/v yeast extract, 0.375% w/v soluble starch, 0.025% w/v dipotassium phosphate, 0.01% w/v magnesium sulfate). All fungal vegetative growth was performed at 21°C and transferred every two weeks by inoculating 25 μl of previous culture to a new flask/plate containing 25 ml of media.

To induce sporulation of *Blastocladiella emersonii* ATCC 22665 and *Chytriomyces confervae* CBS 675.73, 400 ml of liquid PYG was inoculated with 20 ml of vegetative cells in a 2.8 L Fernbach flask, and incubated, with 150 rpm agitation, for 24 h at 21°C. Zoospores were then separated from sporangia using a 20 μm pluriStrainer (pluriSelect) in 50 ml falcon tubes.

Rhizoclosmatium globosum JEL800 zoospore obtention was performed by growing this strain on PYG agar plates for ~4 days. Plates were then flooded with 7 ml of distilled water at 21°C for 15 min followed by removal of zoospore-containing water to a 15 ml falcon tube (this process was repeated twice per plate).

To induce sporulation in *Allomyces macrogynus* Australia_3, vegetative growth was performed for ~5 days in 25 ml of liquid YpSs/4 media. Sporangia were then separated from the culture using sterile tweezers, washed with Volvic Natural Mineral Water, and placed in 20 ml of Volvic water in a 25cm^2 culture flask at 21°C overnight. Zoospores were separated from sporangia using a 20 μm pluriStrainer (pluriSelect) in 50 ml falcon tubes.

METHOD DETAILS

CyclOp pathway gene database searches and phylogenetic analyses

To study the presence or absence of the CyclOp (BeGC1) fusion proteins and the BeCNG1 light-sensing proteins, BLAST[50] and HMM[51] searches were performed against the complete GenBank non-redundant databases[56] (Last searched on January 2022) using the proteins BeGC1 (GenBank: AIC07007.1) and BeCNG1 (GenBank: AIC07008.1) as queries. Additionally, local BLAST searches were performed on 45 publicly available proteomes from fungal and one aphelid species (Table S1).

The phylogenetic datasets were based on the protein datasets of Avelar et al.[8] in which the authors BLAST searched both the guanylyl-cyclase GC1 and the rhodopsin domains of the CyclOp fusion protein and the BeCNG1 channel protein of *B. emersonii* against a database of more than 900 genomes from eukaryotic and prokaryotic species from across the tree of life (see Table S1 of Avelar et al.[8]). MAFFT[52] was used to incorporate identical sequences from the analysed proteomes into a multiple sequence alignment creating a dataset for the complete CyclOp gene fusion, two trimmed datasets for each domain (type 1 rhodopsin and GC1) of the fusion protein and one for the BeCNG1 protein channel[8] (see Figures S1A–S1C, S2, and S3, with alignments available from FigShare). After trimming with TrimAl[53] with the automated1 option Maximum Likelihood (ML), trees were inferred using IQ-TREE[54] under the LG+F+I+G4 model for the complete CyclOp fusion dataset (Figure S1A) and for the type I rhodopsin domain dataset (Figure S1B), LG+R5+C60 model for the GC1 guanylyl cyclase domain dataset (Figure S1C) and LG+C60+I+G4 for the dataset of the BeCNG1 channel protein (Figure S2). All alignments, datasets and trees can be found at Figshare: https://doi.org/10.6084/m9.FigShare.19182086.v4.

Fungal photoreceptors searches across proteomes

To test the presence of fungal photoreceptors across the fungal tree, localBLAST and HMM searches were performed on 45 publicly available proteomes from fungal and (one) aphelid species (Table S1). The queries for blue-light photoreceptor proteins were White Collar Complex proteins (WCC) WC-1, WC-2, Vivid (VVD) (GenBank: ESA41977.1, CAA70336.1, AAK08514.1) and the cryptochrome (CRY; GenBank: EAA36486.3);[5,30] the green-light type I and II rhodopsin (the opsin NOP-1; GenBank: AAD45253.1 and the type 2 opsin-like GPCR; SPPG_00350T0L [see source reference]);[31–33] and the red light phytochromes PHY-1 and PHY-2[5,34,35] (AAZ57422.1 and AAZ57421.1). All these seed sequences were identified from *Neurospora crassa*, with the exception of the type 2 rhodopsin which came from *Spizellomyces punctatus*[5,31] (Table S3).

To study the possible presence of a carotenoid synthesis pathway for retinal production, a combination of previous datasets for carotenoid biosynthesis and cleavage enzymes from one giant virus (ChoanoV1), two choanoflagellates and two haptophytes,[48] together with the carotenoid biosynthesis enzymes found in *B. emersonii*[8] was used. This dataset also included three enzymes for early sterol and carotenoid biosynthesis (isoprenoid biosynthesis steps).[48] The protein sequences from this dataset were BLAST searched[50] (BLASTp) against the proteomes of the 44 selected fungal species (Table S1). All results were confirmed by reciprocal BLASTp searches of the candidate sequences identified to the NCBI non-redundant protein sequence database and HMMscan searches[51] (see Table S3).

Synthesis of ultrastructure, protein architecture and evolutionary data

Ultrastructural information about intracellular architecture and localization of the side-body complexes and lipid bodies used for the drawings in Figure 1B came from ultrastructural studies of zoosporic fungi[10,11,24,25,57–62] (Table S2). Representation of the CyclOp protein and its domains from Figure 1 are based on the CyclOp domain alignment architecture (Figure S3) and the domain hits obtained for each sequence with HMMR/BLAST web server tools.[51,56]

Fluorescence microscopy

Zoospores were collected from the culture medium by concentrating them by centrifugation at 1000 x g for 5 min followed by removal of the supernatant. In all cases the remaining pellet was fixed in 0.5 ml of 4% w/v paraformaldehyde in 1X PBS and transferred into a 15 ml Falcon tube for 15 min at room temperature. Cells were concentrated by centrifugation and resuspended in PBS for a first washing step. The cell pellet was then resuspended and permeabilized in 0.5 ml of PBS containing 0.1% v/v Triton X-100. A second washing step in 0.5 ml of PBS was performed and then the cells were blocked with 0.5 ml of 1% w/v BSA in PBS and incubated for 45 min at room temperature, followed by the addition of the primary antibody α-tubulin DM1A (Sigma-Aldrich, Cat# T6199, RRID: AB_477583) at a concentration of 1:500 v/v for 120 min at room temperature. After a third washing step of the primary antibody with 1X PBS, the secondary antibody Alexa Fluor 647 Goat anti-mouse IgG1 antibody was added (Thermo Fisher Scientific, Cat#A-21240, RRID: AB_2535809) to the PBS-resuspended fixed zoospore solution for 60 min at room temperature. Alexa Fluor 488 Phalloidin (Invitrogen, Cat# A12379, RRID: AB_2759222) and Nile Red (Thermo Fisher Scientific, Cat# N1142) were also added at a 1:500 v/v concentration for 60 min at room temperature. After two washing steps with 1X PBS, the final cell pellets were resuspended in 100 μl of 20% v/v Citifluor AF2 Antifadent Mountant Solution and 7 μl was placed on a slide and covered with a coverslip, which was then sealed with transparent nail polish on the edges to avoid evaporation. Cells were imaged on a Zeiss LSM-780 inverted high-resolution laser scanning confocal microscope with a Ph3 ×100 oil objective. Exposures were kept constant during experiments, and images were collected using BLACK ZEN Software (ZEN Digital Imaging for Light Microscopy), and analyzed/formatted with Fiji.[49]

QUANTIFICATION AND STATISTICAL ANALYSIS

Best-fitting phylogenetic models were selected with the IQ-TREE[54] TESTNEW algorithm as per BIC for the all datasets, obtaining the following best-fitting models: LG+F+I+G4 for the complete CyclOp fusion dataset (Figure S1A) and for the type I rhodopsin domain dataset (Figure S1B), LG+R5+C60 model for the GC1 guanylyl cyclase domain dataset (Figure S1C) and LG+C60+I+G4 for the dataset of the BeCNG1 channel protein (Figure S2). Statistical support was evaluated using 1000 ultrafast bootstrap replicates and 1000 replicates of the SH-like approximate likelihood ratio test and the resulting trees were visualized with FigTree.[55]

Current Biology, Volume *32*

Supplemental Information

A light-sensing system

in the common ancestor of the fungi

Luis Javier Galindo, David S. Milner, Suely Lopes Gomes, and Thomas A. Richards

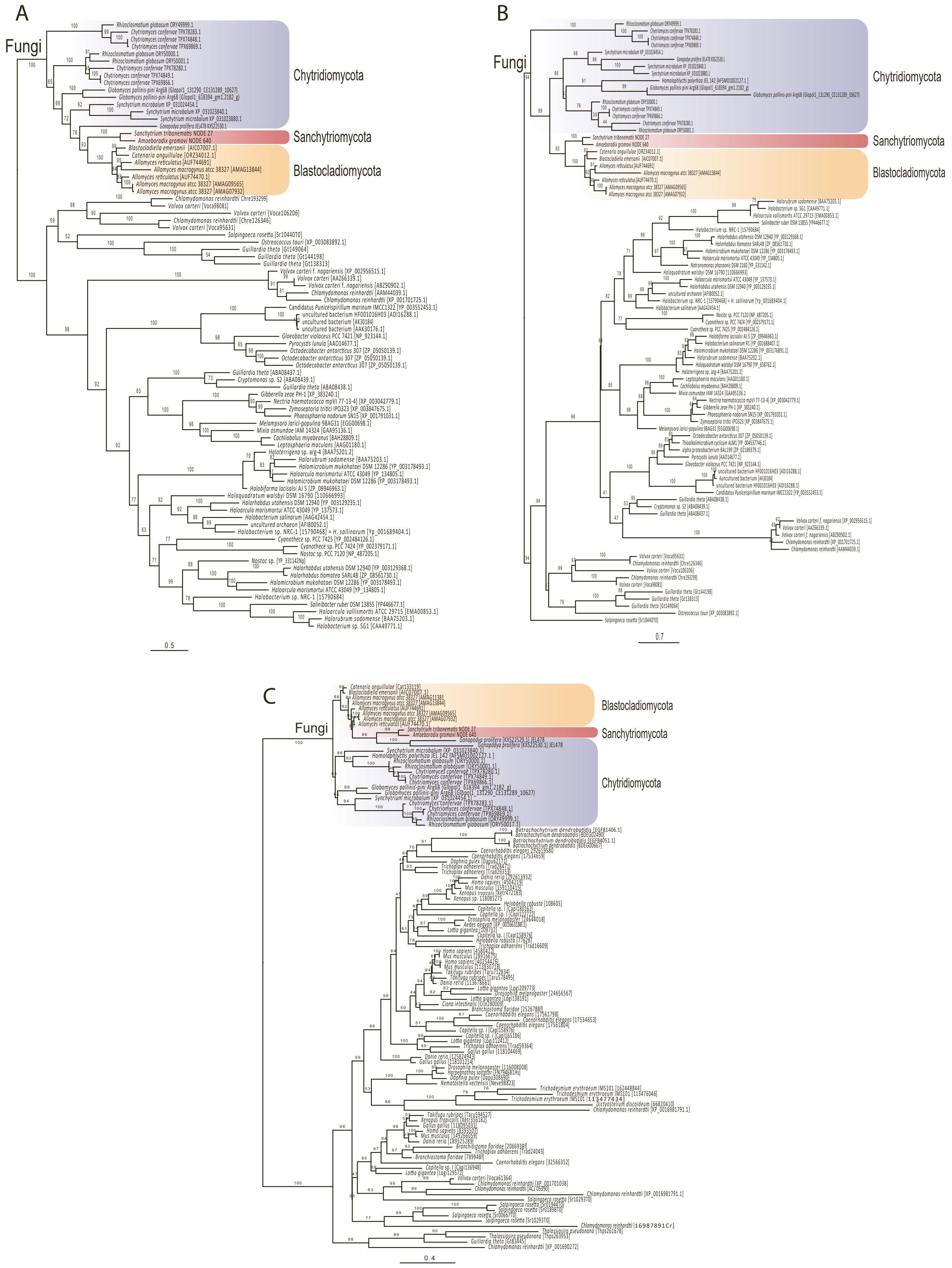
A
Fungi
Chytridiomycota
Sanchytriomycota
Blastocladiomycota
0.5
B
Fungi
Chytridiomycota
Sanchytriomycota
Blastocladiomycota
0.7
C
Fungi
Blastocladiomycota
Sanchytriomycota
Chytridiomycota
0.4

Figure S1. Maximum likelihood tree based on the protein dataset of Avelar et al. (2014)[S1], related to Figure 1. (A) Phylogenetic reconstruction of the CyclOp (BeGC1) protein-fusion, with 84 sequences and 3095 amino acidic positions, the tree was inferred using IQ-TREE under the LG+F+I+G4 model with 1000 ultrafast bootstrap as statistical support. **(B)** Phylogenetic reconstruction of the Type I bacterial rhodopsin of the CyclOp (BeGC1) protein-fusion, with 85 sequences and 127 amino acidic positions, the tree was inferred using IQ-TREE under the LG+F+I+G4 model with 1000 ultrafast bootstrap as statistical support. **(C)** Phylogenetic reconstruction of the GC1 guanylyl-cyclase domain of the CyclOp (BeGC1) protein-fusion, with 107 sequences and 157 amino acidic positions, the tree was inferred using IQ-TREE under the LG+R5+C60 model with 1000 ultrafast bootstrap as statistical support. These results underpin the summary data figure shown in Figure 1.

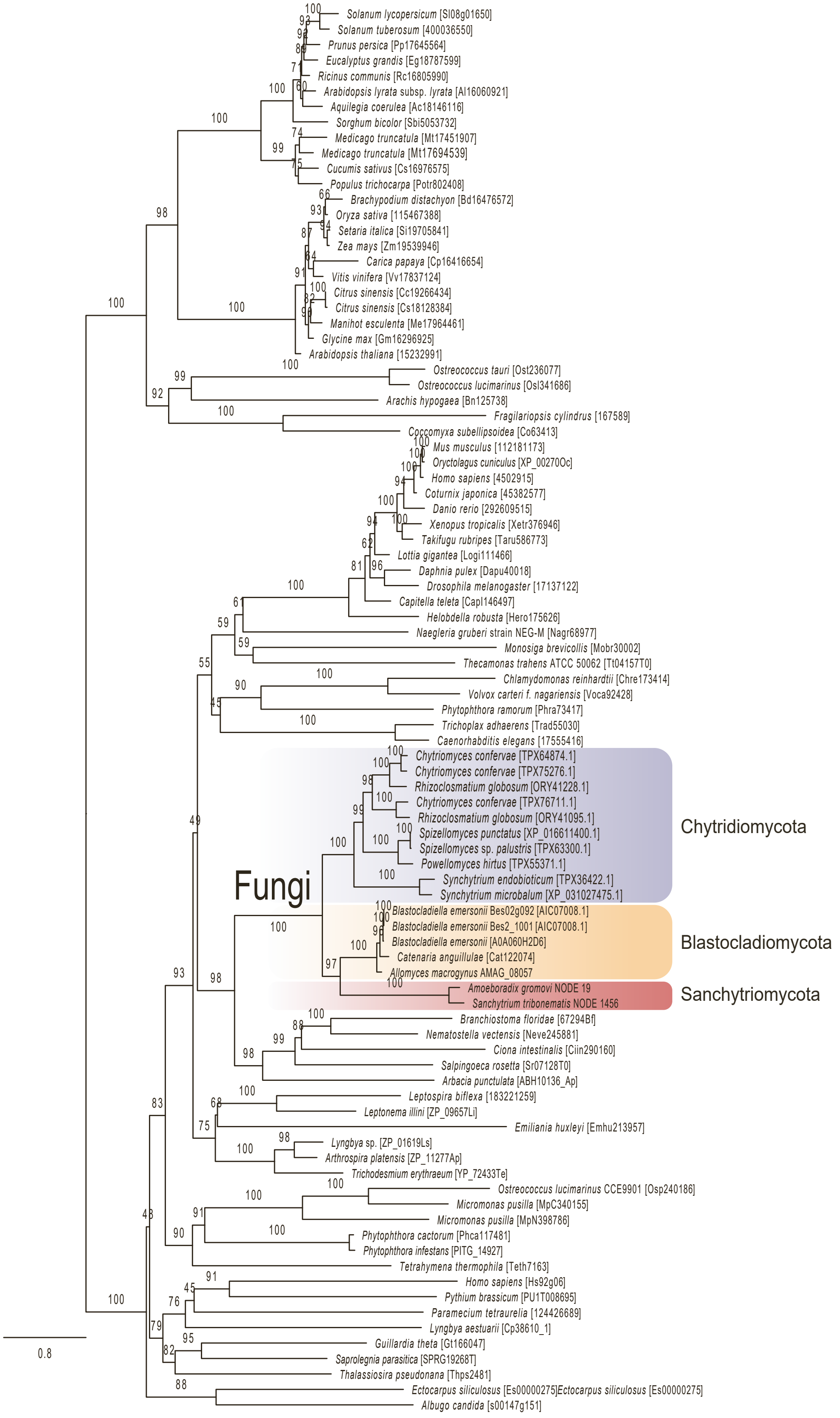

Figure S2. Maximum likelihood tree based on the protein dataset of Avelar et al. (2014)[S1], related to Figure 1. Phylogenetic reconstruction of the cyclic nucleotide gated channel BeCNG1, with 91 sequences and 307 amino acidic positions, the tree was inferred using IQ-TREE under the LG+C60+I+G4 model with 1000 ultrafast bootstrap as statistical support. These results underpin the summary data figure shown in Figure 1.

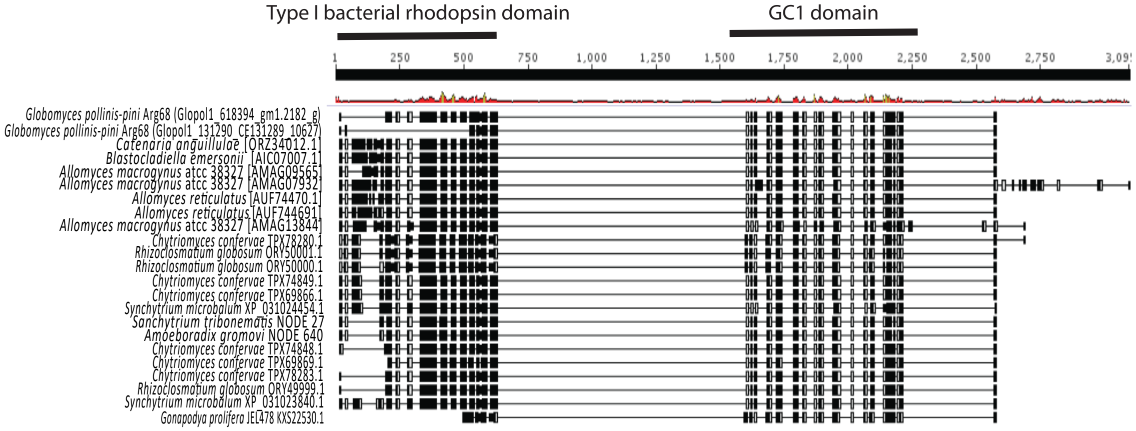

Figure S3. Alignment of CyclOp sequences from Chytridiomycota, Sanchytriomycota and Blastocladiomycota based on the protein dataset of Avelar et al. (2014)[S1], related to Figure 1. The alignment was made using MAFFT. These results underpin the summary data figure shown in Figure 1.

Taxon	Database used	BioProject	BioSample	Sample source	Assembly/SRA/EST ID	Year	Reference publication
Paraphelidium tribonemae X-108	NCBI	PRJNA402032	SAMN07614855 / SAMN07614854	ESE lab, CNRS, Paris-Saclay University	SRR6014119 / SRR6014120	2018	Torruella et al. [S2]
Gonapodya prolifera JEL478	NCBI/JGI	PRJNA207863	SAMN02746072	Joint Genome Institute	GCA_001574975.1 Ganpr1	2015	Chang et al. [S3]
Anaeromyces robustus S4	NCBI	PRJNA330692	SAMN05421914	Joint Genome Institute	GCA_002104895.1 Anaeromyces sp. S4 v1.0	2017	Mondo et al. [S4]
Piromyces sp. E2	NCBI	PRJNA82799	SAMN00788684	Joint Genome Institute	GCA_002157105.1 PirspE2 v1.0	2017	Mondo et al. [S4]
Synchytrium microbalum JEL517	NCBI	PRJNA576245	SAMN08987497	Agriculture and Agri-Food Canada	GCA_006535985.1 ASM653598v1	2019	van de Vossenberg et al. [S5]
Rhizoclosmatium globosum JEL800	NCBI	PRJNA330693	SAMN05421919	Joint Genome Institute	GCA_002104985.1 Rhihy1	2017	Mondo et al. [S4]
Chytriomyces confervae CBS 675.73	NCBI	PRJNA453741	SAMN08987275	Agriculture and Agri-Food Canada	GCA_006535975.1	2019	van de Vossenberg et al. [S5]
Spizellomyces punctatus BR117	NCBI	PRJNA319336	SAMN00716787	Broad Institute	GCA_000182565.2 S_punctatus_V1	2016	Russ et al. [S6]
Globomyces pollinis-pini Arg68	Joint Genome Institute	Project ID: 1098991	Project ID: 1098991	Joint Genome Institute	Glopol1_AssemblyScaffolds.fasta.gz	2016	https://genome.jgi.doe.gov/portal /Glopol1/Glopol1.download.html
Batrachochytrium dendrobatidis JAM81	NCBI	PRJNA41157	SAMN02746048	Joint Genome Institute	GCA_000203795.1 v1.0	2011	Joneson et al. [7]
Homolaphlyctis polyrhiza JEL142	NCBI	PRJNA68115	SAMN02981350	University of Idaho	GCA_000235945.1 HOMPOL_1.0	2011	Joneson et al. [7]
Amoeboradix gromovi X-113	NCBI	PRJNA668693	SAMN16418512	ESE lab, CNRS, Paris-Saclay University	PRJNA668693	2021	Galindo et al.[8]
Sanchytrium tribonematis X-128	NCBI	PRJNA668694	SAMN16418513	ESE lab, CNRS, Paris-Saclay University	PRJNA668694	2021	Galindo et al.[8]
Allomyces macrogynus ATCC 38327	NCBI	PRJNA20563	SAMN02953744	Broad Institute	GCA_000151295.1 A_macrogynus_V3	2015	The Genome Sequence of *Allomyces macrogynus* strain ATCC 38327 / Origins of Multicellularity Sequencing Project, Broad Institute of Harvard and MI
Blastocladiella emersonii ATCC 22665	NCBI	dbEST-Id 25065829 to 25082878.	dbEST-Id 25065829 to 25082878.	Universidade de São Paulo	dbEST:CO961503-CO978552	2005	Ribichich et al. [S9]
Catenaria anguillulae PL171	NCBI	PRJNA330705	SAMN05421822	Joint Genome Institute	GCA_002102555.1 Catan2	2017	Mondo et al. [S4]
Olpidium bornovanus S191	NCBI	NCBI	SAMN05920846	Joint Genome Institute	GCA_017911155.1	2021	Chang et al. [S10]
Basidiobolus meristosporus CBS 931.73	NCBI	PRJNA196075	SAMN02745972	Joint Genome Institute	GCA_002104905.1 Basme2finSC	2017	Mondo et al. [S4]
Basidiobolus heterosporus B8920	NCBI	PRJNA211915	SAMN02371514	Institute for Genome Sciences, University of Maryland	GCA_000697455.1 BasHetB8920-1.0	2016	Chibucos et al.[S11]
Conidiobolus coronatus NRRL 28638	NCBI	PRJNA67455	SAMN00779232	Joint Genome Institute	GCA_001566745.1 Conidiobolus coronatus NRRL28638	2015	Chang et al. [S3]
Entomophthora muscae KVL-14-117	NCBI	PRJEB10825	SAMEA3545222	University of Copenhagen	GCA_900018355.1 Em_KVL-14-118	2017	De Fine Licht et al.[S12]
Coemansia reversa NRRL 1564	NCBI	PRJNA68631	SAMN00631612	Joint Genome Institute	GCA_002705745.1 Coere1	2015	Chang et al. [S3]
Capniomyces stellatus MIS-10-108	NCBI	PRJNA315385	SAMN04558131	University of Toronto	GCA_001661515.1 ASM166151v1	2016	Wang et al.[S13]
Glomus cerebriforme DAOM 227022	NCBI	PRJNA430010	SAMN08364855	INRA	GCA_003550305.1 ASM355030v1	2019	Morin et al.[S14]
Rhizophagus irregularis DAOM 197198	NCBI	PRJNA471752	SAMN02744054	Joint Genome Institute	GCA_000439145.3 ASM43914v3	2018	Chen et al.[S15]
Mortierella antarctica KOD 1229	NCBI	PRJNA340822	SAMN05720774	Joint Genome Institute	GCA_015849325.1_UCR_MspKOD1229_1.0	2020	Vandepol et al.[S16]
Mortiereinala alpina ATCC 32222	NCBI	PRJNA41211	SAMN02981246	Jiangnan Universit / Nankai University / Wake Forest University School of Medicine	GCA_000240685.2 ASM24068v2	2011	Wang et al.[S17]
Mucor circinelloides JCM 22480	NCBI	PRJNA537675	SAMN11512151	Joint Genome Institute	GCA_001599575.1 JCM_22480_assembly_v001	2019	Navarro-Mendoza et al.[S18]
Umbelopsis isabellina B7317	NCBI	PRJNA211912	SAMN02370994	Institute for Genome Sciences, University of Maryland	GCA_000697415.1 UmbIsaB7317-1.0	2014	Chibucos et al.[S11]
Rhizopus delemar RA 99-880	NCBI	PRJNA13066	SAMN02953598	Broad Institute	GCA_000149305.1_RO3	2009	Ma et al.[S19]
Rhizopus oryzae 99-892	NCBI	PRJNA186020	SAMN02352643	Institute for Genome Sciences, University of Maryland	GCA_000697725.1 RhiOry99-892-1.0	2016	Chibucos et al.[S11]
Phycomyces blakesleeanus NRRL 1555	NCBI	PRJNA342701	SAMN00189023	Joint Genome Institute	GCA_001638985.2 Phybl2	2016	Corrochano et al.[S20]
Cryptococcus neoformans var. grubii H99	NCBI	PRJNA177334	SAMN03081441	Broad Institute/ Duke University	GCA_000149245.3 CNA3	2014	Janbon et al.[S21]
Ustilago maydis 521	NCBI	PRJNA14007	SAMN02900459	Broad Institute	GCA_000328475.2 Umaydis521_2.0	2006	Kämper et al.[S22]
Puccinia graminis f. sp. *tritici* CRL 75-36-700-3	NCBI	PRJNA66375	SAMN00013043	Broad Institute	GCA_000149925.1 ASM14992v1	2011	Duplessis et al.[S23]
Coprinopsis cinerea okayama7#130	NCBI	PRJNA1447	SAMN02953595	Broad Institute	GCF_000182895.1_CC3	2010	Stajich et al.[S24]
Aspergillus nidulans FGSC A4	NCBI	PRJNA130	SAMN02953587	Broad Institute	GCF_000149205.2_ASM14920v2	2005	Galagan et al.[S25]

Trichoderma atroviride IMI 206040	NCBI	PRJNA19867	SAMN02744066	Joint Genome Institute	GCF_000171015.1_TRIAT_v2.0	2011	Kubicek et al.[S26]
Neurospora crassa OR74A	NCBI	PRJNA13841	SAMN02953583	Broad Institute	GCF_000182925.2_NC12	2003	Galagan et al.[S27]
Fusarium oxysporum NRRL 32931	NCBI	PRJNA67067	SAMN02981331	Broad Institute	GCF_000271745.1_FO_FOSC_3_a_V1	2018	DeIulio et al.[S28]
Saitoella complicata NRRL Y-17804	NCBI	PRJNA352842	SAMN00760712	Joint Genome Institute	GCA_001661265.1 Saico1	2016	Riley et al.[S29]
Sclerotinia sclerotiorum 1980 UF-70	NCBI	PRJNA15530	SAMN02953621	Broad Institute	GCA_000146945.2 ASM14694v2	2011	Amselem et al.[S30]
Saccharomyces cerevisiae S288C	NCBI	PRJNA128	PRJNA128	Saccharomyces Genome Database	GCA_000146045.2	1996	Goffeau et al.[S31]
Schizosaccharomyces pombe (ASM294v2)	NCBI	PRJNA127	SAMEA3138176	Universite de Montreal	GCA_000002945.2 ASM294v2	2007	Wood et al.[S32]

Table S1. List of the genomes/proteomes used in this study, Related to STAR Methods.

Taxon	Publication	Year	Light or Electron Microscopy?
Blastocladiella emersonii	Reichle et al.[S33]	1967	Light and Electron Microscopy
Blastocladiella emersonii	Cantino et al.[S34]	1970	Electron Microscopy
Catenaria anguillulae	Manier[S35]	1977	Electron Microscopy
Allomyces macrogynus	Fuller et al.[S36]	1971	Electron Microscopy
Sanchytrium tribonematis	Karpov et al.[S37]	2019	Light and Electron Microscopy
Amoeboradix gromovi	Karpov et al.[S38]	2018	Light and Electron Microscopy
Chytriomyces confervae	Barr et al.[S39]	1976	Electron Microscopy
Globomyces pollinis-pini	Letcher et al.[S40]	2008	Light and Electron Microscopy
Rhizoclosmatium globosum	Barr et al.[S39]	1976	Electron Microscopy
Synchytrium microbalum	Longcore et al.[S41]	2016	Light and Electron Microscopy
Gonapodya prolifera	Mollicone et al.[S42]	1999	Light and Electron Microscopy

Table S2. Origin of the microscopic data used to discuss and represent the lipid droplets in Zoospores, Related to Figure 1 and STAR Methods.

<table>
<tr><th></th><th colspan="8">CyclOp pathway</th></tr>
<tr><th>Species and strain</th><th>CyclOp</th><th>evalue</th><th>Rhodopsin type I</th><th>evalue</th><th>GC1</th><th>evalue</th><th>BeCNG1</th><th>evalue</th></tr>
<tr><td>Paraphelidium tribonemae X-108</td><td>NF</td><td>NF</td><td>NF</td><td>NF</td><td>NF</td><td>NF</td><td>NF</td><td>NF</td></tr>
<tr><td>Gonapodya prolifera JEL478</td><td>>KXS22530.1_adenylyl_cyclase_[Gonapodya_prolifera_JEL478]</td><td>1.00E-95</td><td>PARTIAL >KXS22530.1_adenylyl_cyclase_[Gonapodya_prolifera_JEL478]</td><td></td><td>>KXS22530.1_adenylyl_cyclase_[Gonapodya_prolifera_JEL478]</td><td>4.00E-83</td><td>NF</td><td>NF</td></tr>
<tr><td>Anaeromyces robustus S4</td><td>NF</td><td>NF</td><td>NF</td><td>NF</td><td>NF</td><td>NF</td><td>NF</td><td>NF</td></tr>
<tr><td>Piromyces sp. E2</td><td>NF</td><td>NF</td><td>NF</td><td>NF</td><td>NF</td><td>NF</td><td>NF</td><td>NF</td></tr>
<tr><td>Synchytrium microbalum JEL517</td><td>XP_031023840.1 uncharacterized protein SmJEL517_g04188, XP_031024454.1 uncharacterized protein SmJEL517_g03632</td><td>0.00E+00</td><td>XP_031023840.1 uncharacterized protein SmJEL517_g04188, XP_031024454.1 uncharacterized protein SmJEL517_g03632</td><td>2E-27, 9e-12</td><td>XP_031023840.1 uncharacterized protein SmJEL517_g04188, XP_031024454.1 uncharacterized protein SmJEL517_g03632</td><td>2e-102, 1e-85</td><td>XP_031027475.1 uncharacterized protein SmJEL517_g00458</td><td>2.00E-118</td></tr>
<tr><td>Rhizoclosmatium globosum JEL800</td><td>ORY50000.1 adenylyl cyclase [Rhizoclosmatium globosum], ORY50001.1 adenylyl cyclase [Rhizoclosmatium globosum], ORY49999.1 adenylyl cyclase [Rhizoclosmatium globosum]</td><td>1e-141, 9e-138, 5e-107</td><td>ORY50000.1 adenylyl cyclase [Rhizoclosmatium globosum], ORY50001.1 adenylyl cyclase [Rhizoclosmatium globosum], ORY49999.1 adenylyl cyclase [Rhizoclosmatium globosum]</td><td>7e-19, 5e-18, 5e-04</td><td>ORY50000.1 adenylyl cyclase [Rhizoclosmatium globosum], ORY50001.1 adenylyl cyclase [Rhizoclosmatium globosum], ORY49999.1 adenylyl cyclase [Rhizoclosmatium globosum]</td><td>1e-93, 3e-93, 1e-86</td><td>ORY41228.1 hypothetical protein BCR33DRAFT_718890, ORY41095.1 camp-binding domain-like protein</td><td>2e-125, 2e-119</td></tr>
<tr><td>Chytriomyces confervae CBS 675.73</td><td>TPX69866.1 hypothetical protein CcCBS67573_g06742, TPX74849.1 hypothetical protein CcCBS67573_g03867, TPX78280.1 hypothetical protein CcCBS67573_g00474</td><td>2e-141, 8e-141, 3e-135</td><td>TPX69866.1 hypothetical protein CcCBS67573_g06742, TPX74849.1 hypothetical protein CcCBS67573_g03867, TPX78280.1 hypothetical protein CcCBS67573_g00474</td><td>3e-22, 9e-22, 2e-20</td><td>TPX69866.1 hypothetical protein CcCBS67573_g06742, TPX74849.1 hypothetical protein CcCBS67573_g03867, TPX78280.1 hypothetical protein CcCBS67573_g00474</td><td>3e-90, 1e-89, 2e-89</td><td>TPX64874.1 hypothetical protein CcCBS67573_g08290</td><td>4.00E-165</td></tr>
<tr><td>Spizellomyces punctatus BR117</td><td>NF</td><td>NF</td><td>NF</td><td>NF</td><td>NF</td><td>NF</td><td>XP_016611400.1_hypothetical_protein_SPPG_02404</td><td>1.00E-156</td></tr>
<tr><td>Globomyces pollinis-pini Arg68</td><td>jgi|Glopol1|618394|gm1.2182_g, jgi|Glopol1|131290|CE131289_10627</td><td>8e-129, 3e-111</td><td>jgi|Glopol1|618394|gm1.2182_g, jgi|Glopol1|131290|CE131289_10627</td><td>2.00E-21</td><td>jgi|Glopol1|618394|gm1.2182_g, jgi|Glopol1|131290|CE131289_10627</td><td>2e-99, 1e-96</td><td>NF</td><td>NF</td></tr>
<tr><td>Batrachochytrium dendrobatidis JAM81</td><td>NF</td><td>NF</td><td>NF</td><td>NF</td><td>NF</td><td>NF</td><td>NF</td><td>NF</td></tr>
<tr><td>Homolaphlyctis polyrhiza JEL142</td><td>NF</td><td>NF</td><td>>AFSM01002127.1_Homolaphlyctis_polyrhiza_JEL_142_strain_JEL142_JEL142_contig02416,_whole_genome_shotgun_sequence.p1 type:complete len:162 gc:universal</td><td>8.00E-09</td><td>>AFSM01002127.1_Homolaphlyctis_polyrhiza_JEL_142_strain_JEL142_JEL142_contig02416,_whole_genome_shotgun_sequence.p2 type:complete len:159 gc:universal</td><td>1.00E-74</td><td>NF</td><td>NF</td></tr>
<tr><td>Amoeboradix gromovi X-113</td><td>NODE_640_length_5666_cov_0.633196.p2 type:complete len:532</td><td>6.00E-128</td><td>NODE_640_length_5666_cov_0.633196.p2 type:complete len:532</td><td>6.00E-40</td><td>NODE_640_length_5666_cov_0.633196.p2 type:complete len:532</td><td>9.00E-95</td><td>NODE_19_length_39463_cov_0.396073.p9 type:complete len:394</td><td>9.00E-97</td></tr>
<tr><td>Sanchytrium tribonematis X-128</td><td>NODE_27_length_39007_cov_1.590650.p8_type:complete_len:535_gc</td><td>1.00E-125</td><td>NODE_27_length_39007_cov_1.590650.p8_type:complete_len:535_gc</td><td>1.00E-40</td><td>NODE_27_length_39007_cov_1.590650.p8_type:complete_len:535_gc</td><td>1.00E-101</td><td>NODE_1456_length_1026_cov_0.812298.p1_type:3prime_partial_len</td><td>0.00E+00</td></tr>
<tr><td>Allomyces macrogynus ATCC 38327</td><td>KNE62746.1_hypothetical_protein_AMAG_07932, KNE65585.1_hypothetical_protein_AMAG_09565</td><td>1e-140, 6e-137</td><td>KNE62746.1_hypothetical_protein_AMAG_07932, KNE65585.1_hypothetical_protein_AMAG_09565</td><td>2e-63, 1e-62</td><td>KNE62746.1_hypothetical_protein_AMAG_07932, KNE65585.1_hypothetical_protein_AMAG_09565</td><td>3e-144, 5e-143</td><td>KNE62879.1_hypothetical_protein_AMAG_18930, KNE61876.1_hypothetical_protein_AMAG_07148</td><td>0.00E+00</td></tr>
<tr><td>Blastocladiella emersonii ATCC 22665</td><td>gi|59276301|gb|CO961585.1|CO961585.p2 type:internal len:223</td><td>2.00E-84</td><td>gi|59276301|gb|CO961585.1|CO961585.p2 type:internal len:223</td><td>1.00E-12</td><td>gi|59276301|gb|CO961585.1|CO961585.p2 type:internal len:223</td><td>4.00E-100</td><td>gi|148886264|gb|EE736522.1|EE7365</td><td>0.00E+00</td></tr>
</table>

								22.p1 type:internal len:119	
Catenaria anguillulae PL171	ORZ32123.1_nucleotide_cyclase_[Catenaria_anguillulae_PL171]	2.00E-101	ORZ32123.1_nucleotide_cyclase_[Catenaria_anguillulae_PL171]	2.00E-53	ORZ32123.1_nucleotide_cyclase_[Catenaria_anguillulae_PL171]	5.00E-143	ORZ36627.1_hypothetical_protein_BCR44DRAFT_1512424	0.00E+00
Olpidium bornovanus S191	NF	NF	NF	NF	NF	NF	NF	NF
Basidiobolus meristosporus CBS 931.73	NF	NF	NF	NF	NF	NF	NF	NF
Basidiobolus heterosporus B8920	NF	NF	NF	NF	NF	NF	NF	NF
Conidiobolus coronatus NRRL 28638	NF	NF	NF	NF	NF	NF	NF	NF
Entomophthora muscae	NF	NF	NF	NF	NF	NF	NF	NF
Coemansia reversa NRRL 1564	NF	NF	NF	NF	NF	NF	NF	NF
Capniomyces stellatus MIS-10-108	NF	NF	NF	NF	NF	NF	NF	NF
Glomus cerebriforme	NF	NF	NF	NF	NF	NF	NF	NF
Rhizophagus irregularis DAOM 197198	NF	NF	NF	NF	NF	NF	NF	NF
Mortierella antarctica	NF	NF	NF	NF	NF	NF	NF	NF
Mortiereinala alpina ATCC 32222	NF	NF	NF	NF	NF	NF	NF	NF
Mucor circinelloides JCM 22480	NF	NF	NF	NF	NF	NF	NF	NF
Umbelopsis isabellina B7317	NF	NF	NF	NF	NF	NF	NF	NF
Rhizopus delemar RA 99-880	NF	NF	NF	NF	NF	NF	NF	NF
Rhizopus oryzae 99-892	NF	NF	NF	NF	NF	NF	NF	NF
Phycomyces blakesleeanus NRRL 1555	NF	NF	NF	NF	NF	NF	NF	NF
Cryptococcus neoformans var. grubii H99	NF	NF	NF	NF	NF	NF	NF	NF
Ustilago maydis 521	NF	NF	NF	NF	NF	NF	NF	NF
Puccinia graminis f. sp. tritici CRL 75-36-700-3	NF	NF	NF	NF	NF	NF	NF	NF
Coprinopsis cinerea okayama7#130	NF	NF	NF	NF	NF	NF	NF	NF
Aspergillus nidulans FGSC A4	NF	NF	NF	NF	NF	NF	NF	NF
Trichoderma atroviride IMI 206040	NF	NF	NF	NF	NF	NF	NF	NF
Neurospora crassa OR74A	NF	NF	NF	NF	NF	NF	NF	NF
Fusarium oxysporum NRRL 32931	NF	NF	NF	NF	NF	NF	NF	NF
Saitoella complicata NRRL Y-17804	NF	NF	NF	NF	NF	NF	NF	NF
Sclerotinia sclerotiorum 1980 UF-70	NF	NF	NF	NF	NF	NF	NF	NF
Saccharomyces cerevisiae S288C	NF	NF	NF	NF	NF	NF	NF	NF

Schizosaccharo myces pombe (ASM294v2)	NF	NF	NF	NF	NF	NF	NF	NF

	Carotenoid biosynthesis				
**Species and strain**	**Phytoene dehydrogenase-related protein**	**evalue**	**Phytoene synthase**	**evalue**	**Carotenoid oxygenase**
Paraphelidium tribonemae X-108	NF	NF	NF	NF	Partr_v1_DN27125_c1_g1_i1_m15640_putative_dioxygenase
Gonapodya prolifera JEL478	NF	NF	NF	NF	>KXS15063.1 hypothetical protein M427DRAFT_70166 [Gonapodya prolifera JEL478]
Anaeromyces robustus S4	NF	NF	NF	NF	ORX81586.1_hypothetical_protein_BCR32DRAFT_279584_[Anaeromyces
Piromyces sp. E2	NF	NF	NF	NF	ORX81586.1
Synchytrium microbalum JEL517	>XP_031025712.1 uncharacterized protein SmJEL517_g02419	0.00E+00	NF	NF	>XP_031025338.1 uncharacterized protein SmJEL517_g02720 [Synchytrium microbalum]
Rhizoclosmatium globosum JEL800	>ORY40098.1 phytoene desaturase [Rhizoclosmatium globosum]	1.00E-153	>ORY52898.1 terpenoid synthase [Rhizoclosmatium globosum]	7.00E-90	>ORY47689.1 hypothetical protein BCR33DRAFT_735871 [Rhizoclosmatium globosum]
Chytriomyces confervae CBS 675.73	>TPX74165.1 hypothetical protein CcCBS67573_g04564 [Chytriomyces confervae]	1.00E-178	>TPX77582.1 hypothetical protein CcCBS67573_g01152	9.00E-49	>TPX66659.1 hypothetical protein CcCBS67573_g07753 [Chytriomyces confervae]
Spizellomyces punctatus BR117	NF	NF	NF	NF	XP_016607807.1_hypothetical_protein_SPPG_05143
Globomyces pollinis-pini Arg68	jgi|Glopol1|529184|e_gw1.200.18.1	0.00E+00	jgi|Glopol1|573654|fgenesh1_kg.6_#_299_#_TRINITY_DN5419_c0_g1_i1	3.00E-60	NF
Batrachochytrium dendrobatidis JAM81	NF	NF	NF	NF	NF
Homolaphlyctis polyrhiza JEL142	JH393157.1_Rhizophydiales_sp._JEL142_unplaced_genomic_scaffold	3.00E-56	AFSM01007168.1_Homolaphlyctis_polyrhiza_JEL_142_strain_JEL142_J	3.00E-23	NF
Amoeboradix gromovi X-113	NF	NF	NF	NF	NF
Sanchytrium tribonematis X-128	NF	NF	NF	NF	NF
Allomyces macrogynus ATCC 38327	>KNE66202.1_phytoene_desaturase_[Allomyces_macrogynus_ATCC_38327] / >KNE57458.1_phytoene_desaturase_[Allomyces_macrogynus_ATCC_38327]	0.00E+00	>KNE57899.1_lycopene_cyclase_domain-containing_protein_[Allomyces_macrogynus_ATCC_38327] / >KNE59078.1_lycopene_cyclase_domain-containing_protein_[Allomyces_macrogynus_ATCC_38327]	0.00E+00	>KNE55150.1_hypothetical_protein_AMAG_01073_[Allomyces_macrogynus_ATCC_38327] / >KNE59794.1_hypothetical_protein_AMAG_05253_[Allomyces_macrogynus_ATCC_38327]
Blastocladiella emersonii ATCC 22665	>AIC07010.1_putative_phytoene_dehydrogenase_[Blastocladiella_emersonii]	0.00E+00	>AIC07009.1_putative_lycopene_cyclase_phytoene_synthase_[Blastocladiella_emersonii]	0.00E+00	>AIC07011.1_putative_carotenoid_dioxygenase_[Blastocladiella_emersonii]
Catenaria anguillulae PL171	>ORZ34110.1_hypothetical_protein_BCR44DRAFT_1479970_[Catenaria_anguillulae_PL171]	0.00E+00	>ORZ39450.1_Squalene/phytoene_synthase-domain-containing_protein_[Catenaria_anguillulae_PL171]	1.00E-66	>ORZ40243.1_retinal_pigment_epithelial_membrane_protein-domain-containing_protein_[Catenaria_anguillulae_PL171] / ORZ31212.1_carotenoid_oxygenase_[Catenaria_anguillulae_PL171]
Olpidium bornovanus S191	NF	NF	NF	NF	NF
Basidiobolus meristosporus CBS 931.73	NF	NF	NF	NF	ORY02234.1 carotenoid oxygenase [Basidiobolus meristosporus
Basidiobolus heterosporus B8920	NF	NF	NF	NF	JNET01030896.1_Basidiobolus_heterosporus_B8920_jtg7180000624177
Conidiobolus coronatus NRRL 28638	NF	NF	NF	NF	NF
Entomophthora muscae	NF	NF	NF	NF	NF
Coemansia reversa NRRL 1564	NF	NF	NF	NF	PIA18333.1_hypothetical_protein_COEREDRAFT_79821
Capniomyces stellatus MIS-10-108	NF	NF	NF	NF	NF
Glomus cerebriforme	NF	NF	NF	NF	RIA91701.1 carotenoid oxygenase, partial [Glomus cerebriforme]
Rhizophagus irregularis DAOM 197198	NF	NF	NF	NF	XP_025172812.1 hypothetical protein GLOIN_2v1781123
Mortierella antarctica	NF	NF	NF	NF	KAF9987450.1 hypothetical protein BGZ75_000607
Mortiereinala alpina ATCC 32222	NF	NF	NF	NF	ADAG01001055.1|m.19462_ADAG01001055.1|g.19462_type:complete

Mucor circinelloides JCM 22480	KAF1801867.1 phytoene dehydrogenase [Mucor circinelloides]	0.00E+00	KAF1801868.1 Lycopene beta-cyclase [Mucor circinelloides]	3.00E-95	KAF1807426.1 putative carotene oxygenase [Mucor circinelloides]
Umbelopsis isabellina B7317	JNEQ01000058.1_Umbelopsis_isabellina_B7317_jtg7180000025250f_71	1.00E-123	JNEQ01000058.1_Umbelopsis_isabellina_B7317_jtg7180000025250f_71	2.00E-42	JNEQ01000040.1_Umbelopsis_isabellina_B7317_ctg7180000025259_1
Rhizopus delemar RA 99-880	EIE91958.1 hypothetical protein RO3G_16669	0.00E+00	EIE78626.1 hypothetical protein RO3G_03330	7.00E-56	NF
Rhizopus oryzae 99-892	KK997965.1|m.1783 KK997965.1|g.1783 type:complete len:288 gc	4.00E-105	KK997965.1|m.1782 KK997965.1|g.1782 type:complete len:313 gc	4.00E-36	KK998411.1|m.11289 KK998411.1|g.11289 type:complete len:426
Phycomyces blakesleeanus NRRL 1555	XP_018294564.1 Phytoene dehydrogenase	0.00E+00	XP_018294563.1 Phytoene Synthase/Lycopene cyclase	5.00E-89	XP_018284903.1 hypothetical protein PHYBLDRAFT_183749
Cryptococcus neoformans var. grubii H99	NF	NF	NF	NF	NF
Ustilago maydis 521	XP_011390692.1 putative phytoene dehydrogenase	2.00E-165	XP_011388293.1 hypothetical protein UMAG_06287	4.00E-45	NF
Puccinia graminis f. sp. tritici CRL 75-36-700-3	XP_003337345.2 hypothetical protein PGTG_19044	3.00E-180	XP_003329970.2 hypothetical protein PGTG_11907	6.00E-56	NF
Coprinopsis cinerea okayama7#130	NF	NF	NF	NF	NF
Aspergillus nidulans FGSC A4	NF	NF	NF	NF	XP_659308.1 hypothetical protein AN1704.2
Trichoderma atroviride IMI 206040	NF	NF	NF	NF	XP_013942699.1 hypothetical protein TRIATDRAFT_223967
Neurospora crassa OR74A	XP_964713.1 phytoene desaturase [Neurospora crassa OR74A]	8.00E-148	XP_965725.3 phytoene synthase [Neurospora crassa OR74A]	6.00E-60	XP_001727958.1 carotenoid oxygenase 2 [Neurospora crassa OR74A]
Fusarium oxysporum NRRL 32931	XP_031030490.1 uncharacterized protein FOYG_15803	3.00E-174	XP_031030489.1 uncharacterized protein FOYG_15802	6.00E-76	XP_031040765.1 uncharacterized protein FOYG_08800
Saitoella complicata NRRL Y-17804	XP_019021262.1 phytoene dehydrogenase	0.00E+00	XP_019022354.1 terpenoid synthase	2.00E-67	NF
Sclerotinia sclerotiorum 1980 UF-70	XP_001594533.1 hypothetical protein SS1G_04340	9.00E-158	XP_001594534.1 hypothetical protein SS1G_04341	3.00E-12	NF
Saccharomyces cerevisiae S288C	NF	NF	NF	NF	NF
Schizosaccharomyces pombe (ASM294v2)	NF	NF	NF	NF	NF

	Photoreceptors							
**Species and strain**	**WC-1 (White Collar 1)**	**evalue**	**WC-2 (White Collar 2)**	**evalue**	**VVD (VIVID)**	**evalue**	**CRY (cryptochrome)**	**evalue**
Paraphelidium tribonemae X-108	NF	NF	NF	NF	NF	NF	Partr_v1_DN26245_c1_g1_i3_m48392_putative_cryptochrome	2.00E-30
Gonapodya prolifera JEL478	KXS17110.1 hypothetical protein M427DRAFT_153942	4E-54, 2e-44	KXS18876.1 hypothetical protein M427DRAFT_53357, KXS09899.1 hypothetical protein M427DRAFT_149316	3e-16, 4e-12	NF	NF	KXS15620.1 cryptochrome [Gonapodya prolifera JEL478]	5.00E-73
Anaeromyces robustus S4	NF	NF	NF	NF	NF	NF	NF	NF
Piromyces sp. E2	NF	NF	NF	NF	NF	NF	NF	NF
Synchytrium microbalum JEL517	XP_031027930.1 uncharacterized protein SmJEL517_g00414	5.00E-105	XP_031026964.1 uncharacterized protein SmJEL517_g01144	3.00E-09	NF	NF	XP_031023438.1 uncharacterized protein SmJEL517_g04615	2.00E-87
Rhizoclosmatium globosum JEL800	NF	NF	NF	NF	NF	NF	ORY50805.1 cryptochrome [Rhizoclosmatium globosum]	2.00E-65
Chytriomyces confervae CBS 675.73	TPX74047.1 hypothetical protein CcCBS67573_g04675	2.00E-66	TPX53564.1 hypothetical protein CcCBS67573_g09686	1.00E-13	NF	NF	TPX70061.1 hypothetical protein CcCBS67573_g06678	2.00E-56
Spizellomyces punctatus BR117	XP_016610190.1_hypothetical_protein_SPPG_09046	3.00E-84	XP_016606504.1_PAS_domain_S-box_protein, XP_016612182.1_white_collar_2_protein	8e-17, 3e-13	NF	NF	NF	NF
Globomyces pollinis-pini Arg68	jgi|Glopol1|579169|fgenesh1_kg.24_#_249_#_TRINITY_DN4747_c0_g1_i7	1.00E-74	jgi|Glopol1|437558|CE437557_9169	2.00E-14	NF	NF	NF	NF
Batrachochytrium	NF	NF	NF	NF	NF	NF	NF	NF

dendrobatidis JAM81								
Homolaphlyctis polyrhiza JEL142	NF	NF	NF	NF	NF	NF	NF	NF
Amoeboradix gromovi X-113	NODE_13_length_42895_cov_0.518413.p8 type:complete l	6.00E-70	NODE_154_length_16838_cov_0.293327.p8 type:complete len:220 gc	6.00E-22	NF	NF	NODE_14_length_42432_cov_0.433799.p8 type:complete len:490	1.00E-15
Sanchytrium tribonematis X-128	NODE_754_length_4199_cov_1.046829.p1_type:complete_len:558_gc:universal, NODE_244_length_11882_cov_0.560214.p4_type:complete_len:343_gc, NODE_1173_length_1905_cov_0.460133.p1_type:3prime_partial_len	4E-62, 1e-59, 5e-51	NODE_287_length_10726_cov_0.240614.p3_type:complete_len:219_gc, NODE_10_length_61504_cov_0.956502.p34_type:complete_len:204_gc	3.00E-23	NF	NF	NODE_31_length_37178_cov_0.413576.p6_type:complete_len:479, NODE_911_length_3104_cov_0.287188.p1_type:complete_len:509	2e-71, 4e-20
Allomyces macrogynus ATCC 38327	KNE69080.1_PAS_domain_S-box_protein	1.00E-53	KNE64375.1_PAS_domain_S-box_protein, KNE70175.1_hypothetical_protein_AMAG_15144	9E-20, 5e-14	NF	NF	KNE59778.1_hypothetical_protein_AMAG_05242,	2.00E-25
Blastocladiella emersonii ATCC 22665	NF	NF	gi|59287008|gb|CO972292.1|CO972292.p1 type:internal	1.00E-08	NF	NF	NF	NF
Catenaria anguillulae PL171	ORZ34267.1_PAS_domain-domain-containing_protein_[Catenaria_	1.00E-119	ORZ34902.1_hypothetical_protein_BCR44DRAFT_49868	1.00E-18	NF	NF	ORZ31450.1_FAD_binding_domain_of_DNA_photolyase-domain-containing_protein, ORZ37966.1_DNA_photolyase,_FAD-binding/Cryptochrome	5E-29, 1e-20
Olpidium bornovanus S191	KAG5462463.1 hypothetical protein BJ554DRAFT_5016, partial [Olpidium bornovanus]	3.00E-30	NF	NF	NF	NF	NF	NF
Basidiobolus meristosporus CBS 931.73	ORY03092.1 hypothetical protein K493DRAFT_311836 [Basidiobolus meristosporus CBS 931.73], ORX92749.1 hypothetical protein K493DRAFT_285400, ORX78940.1 hypothetical protein K493DRAFT_293436, ORX82576.1 hypothetical protein K493DRAFT_270021, ORX82533.1 hypothetical protein K493DRAFT_91526, ORY05666.1 hypothetical protein K493DRAFT_274936, ORY06962.1 hypothetical protein K493DRAFT_251462	3E-162, 2e-135, 3e-134, 4e-131, 6e-131, 2e-129, 3e-128	ORY05759.1 hypothetical protein K493DRAFT_159907, ORX90491.1 hypothetical protein K493DRAFT_317922, ORY00637.1 hypothetical protein K493DRAFT_278641	4E-35, 3e-34, 6e-34	NF	NF	ORX90230.1 cryptochrome [Basidiobolus meristosporus CBS 931.73], ORX77140.1 cryptochrome [Basidiobolus meristosporus CBS 931.73]	3e-84, 4e-84
Basidiobolus heterosporus B8920	JNET01032621.1_Basidiobolus_heterosporus_B8920_jtg7180000793710	7.00E-164	JNET01033074.1_Basidiobolus_heterosporus_B8920_jtg7180000785454	3.00E-36	NF	NF	JNET01017388.1_Basidiobolus_heterosporus_B8920_ctg7180000781388	1.00E-24
Conidiobolus coronatus NRRL 28638	KXN70665.1 hypothetical protein CONCODRAFT_75611, KXN67054.1 hypothetical protein CONCODRAFT_19863	8E-141, 2e-82	KXN71654.1 hypothetical protein CONCODRAFT_57247 [Conidiobolus	1.00E-37	NF	NF	NF	NF
Entomophthora muscae	KAF7747831.1 blue light receptor [Entomophthora muscae], KAF7750602.1 blue light receptor [Entomophthora muscae, KAF7748558.1 blue light receptor [Entomophthora muscae]	2E-126, 2e-118, 1e-117	KAF7744128.1 blue light receptor [Entomophthora muscae], KAF7744124.1 blue light receptor [Entomophthora muscae]	5e-41, 5e-41	NF	NF	KAF7758463.1 hypothetical protein DSO57_009719, KAF7723467.1 hypothetical protein DSO57_008515	4e-31, 5e-31
Coemansia reversa NRRL 1564	NF	NF	NF	NF	NF	NF	NF	NF
Capniomyces stellatus MIS-10-108	NF	NF	NF	NF	NF	NF	NF	NF
Glomus cerebriforme	RIA85642.1 PAS domain-containing protein [Glomus cerebriforme]	8.00E-170	RIA81527.1 hypothetical protein C1645_810010 [Glomus cerebriforme]	1.00E-58	NF	NF	NF	NF
Rhizophagus irregularis DAOM 197198	XP_025178457.1 hypothetical protein GLOIN_2v1604095	1.00E-164	XP_025179286.1 hypothetical protein GLOIN_2v1477793	2.00E-39	NF	NF	ORY50805.1 cryptochrome [Rhizoclosmatium globosum]	2.00E-65
Mortierella antarctica	NF	NF	NF	NF	NF	NF	NF	NF
Mortiereinala alpina ATCC 32222	NF	NF	NF	NF	NF	NF	KAF9986046.1 hypothetical protein BGZ75_002275	1.00E-21

Mucor circinelloides JCM 22480	KAF1797780.1 hypothetical protein FB192DRAFT_1333251, KAF1804453.1 hypothetical protein FB192DRAFT_1357808, KAF1802616.1 putative white-collar-1b protein	3e-149, 2e-139, 5e-131	KAF1800623.1 hypothetical protein FB192DRAFT_1459016	1.00E-50	NF	NF	KAF1806586.1 hypothetical protein FB192DRAFT_1351151	4.00E-81
Umbelopsis isabellina B7317	JNEQ01000052.1_Umbelopsis_isabellina_B7317_ctg7180000025278_1	6.00E-85	JNEQ01000055.1_Umbelopsis_isabellina_B7317_jtg7180000025233f_71, JNEQ01000041.1_Umbelopsis_isabellina_B7317_ctg7180000025260_	6e-40, 3e-35	NF	NF	NF	NF
Rhizopus delemar RA 99-880	EIE91627.1 hypothetical protein RO3G_16338 [Rhizopus delemar, EIE85287.1 hypothetical protein RO3G_09997 [Rhizopus delemar, EIE89562.1 hypothetical protein RO3G_14273 [Rhizopus delemar	2e-145, 1e-131, 3e-112	EIE76746.1 hypothetical protein RO3G_01450, EIE79173.1 hypothetical protein RO3G_03878, EIE77397.1 hypothetical protein RO3G_02101, EIE89380.1 hypothetical protein RO3G_14091, EIE77889.1 hypothetical protein RO3G_02593	2e-29, 5e-29, 6e-29, 6e-27, 1e-23	NF	NF	EIE91586.1 hypothetical protein RO3G_16297	6.00E-66
Rhizopus oryzae 99-892	KK998731.1|m.24138 KK998731.1|g.24138	2.00E-63	KK998731.1|m.24138 KK998731.1|g.24138 type:complete len:355, KK997983.1|m.2044 KK997983.1|g.2044 type:complete len:350, KK998731.1|m.24143 KK998731.1|g.24143 type:complete len:269, KK998423.1|m.11777 KK998423.1|g.11777 type:complete len:251	2e-63, 4e-39, 1e-30,1e-29	NF	NF	KK997983.1|m.2048 KK997983.1|g.2048 type:complete len:333	7.00E-53
Phycomyces blakesleeanus NRRL 1555	XP_018283657.1 GATA-type zinc finger transcription factor, XP_018295305.1 hypothetical protein PHYBLDRAFT_76314, XP_018288826.1 GATA-type zinc finger transcription factor	6e-153, 2e-130, 4e-118	XP_018290827.1 GATA-type zinc finger transcription factor, XP_018296558.1 GATA-type zinc finger transcription factor, XP_018291784.1 GATA-type zinc finger transcription factor, XP_018296714.1 GATA-type zinc finger transcription factor	1e-53, 7e-51, 7e-26, 3e-25	NF	NF	XP_018298182.1 hypothetical protein PHYBLDRAFT_85761	6.00E-82
Cryptococcus neoformans var. grubii H99	XP_012048765.1 white collar 1 protein	1.00E-110	XP_012050148.1 white collar 2 protein [Cryptococcus neoformans]	8.00E-14	NF	NF	NF	NF
Ustilago maydis 521	XP_011389633.1 hypothetical protein UMAG_03180	1.00E-94	XP_011389049.1 hypothetical protein UMAG_02664	9.00E-31	NF	NF	XP_011392255.1 hypothetical protein UMAG_05917, XP_011387082.1 hypothetical protein UMAG_01131	6e-87, 3e-84
Puccinia graminis f. sp. tritici CRL 75-36-700-3	XP_003321153.2 hypothetical protein PGTG_02195	3.00E-88	XP_003321153.2 hypothetical protein PGTG_02195	4.00E-32	NF	NF	XP_003326630.1 hypothetical protein PGTG_07608	3.00E-100
Coprinopsis cinerea okayama7#130	XP_001832659.1 photoreceptor A [Coprinopsis cinerea okayama7#130]	1.00E-72	XP_001831710.2 hypothetical protein CC1G_12230	2.00E-12	NF	NF	NF	NF
Aspergillus nidulans FGSC A4	XP_661040.1 hypothetical protein AN3436.2	3.00E-157	XP_661211.1 hypothetical protein AN3607.2	4.00E-60	XP_661039.1 hypothetical protein AN3435.2	2.00E-53	NF	NF
Trichoderma atroviride IMI 206040	XP_013938043.1 blue light photoreceptor BLR1	0.00E+00	XP_013938453.1 blue light receptor BLR2	6.00E-147	XP_013942393.1 putative PAS-domain protein envoy	3.00E-43	XP_013941790.1 photolyase [Trichoderma atroviride IMI 206040]	5.00E-99
Neurospora crassa OR74A	XP_011395153.1 white collar 1 protein, variant 3, XP_011395152.1 white collar 1 protein, variant 1, XP_011395152.1 white collar 1 protein, variant 2, XP_011395151.1 white collar 1 protein	0.00E+00	XP_963819.3 zinc finger white collar protein WC2	0.00E+00	AAK08514.1_vivid_PAS_protein_VVD_[Neurospora_crassa]	0.00E+00	XP_965722.3 cryptochrome DASH [Neurospora crassa OR74A]	0.00E+00
Fusarium oxysporum NRRL 32931	XP_031050442.1 uncharacterized protein FOYG_01134	0.00E+00	XP_031041715.1 cutinase palindrome-binding protein	0.00E+00	XP_031037528.1 uncharacterized protein FOYG_09732	2.00E-47	XP_031038092.1 uncharacterized protein FOYG_10105	0.00E+00

Saitoella complicata NRRL Y-17804	XP_019027442.1 hypothetical protein SAICODRAFT_106826, XP_019022537.1 hypothetical protein SAICODRAFT_83374, partial	2E-141, 3e-97	XP_019027026.1 hypothetical protein SAICODRAFT_16945	8.00E-72	NF	NF	NF	NF
Sclerotinia sclerotiorum 1980 UF-70	XP_001586924.1 hypothetical protein SS1G_11953	0.00E+00	XP_001587208.1 hypothetical protein SS1G_12238	2.00E-128	XP_001596024.1 hypothetical protein SS1G_02240	7.00E-42	XP_001593735.1 hypothetical protein SS1G_05163	0.00E+00
Saccharomyces cerevisiae S288C	NF	NF	NF	NF	NF	NF	NF	NF
Schizosaccharomyces pombe (ASM294v2)	NF	NF	NF	NF	NF	NF	NF	NF

Photoreceptors

Species and strain	NOP-1 (Opsin)	evalue	Type 2 rhodopsin-like GPCR	evalue	PHY (phytochromes PHY-1 and PHY-2))	evalue
Paraphelidium tribonemae X-108	NF	NF	NF	NF	NF	NF
Gonapodya prolifera JEL478	KXS16496.1 family A G protein-coupled receptor-like protein	9.00E-27	KXS11833.1 hypothetical protein M427DRAFT_35407, KXS12111.1 family A G protein-coupled receptor-like protein	5e-05, 9e-05	KXS14916.1 hypothetical protein M427DRAFT_123888	0.00E+00
Anaeromyces robustus S4	NF	NF	NF	NF	NF	NF
Piromyces sp. E2	NF	NF	NF	NF	NF	NF
Synchytrium microbalum JEL517	NF	NF	XP_031024018.1 uncharacterized protein SmJEL517_g04095	2.00E-04	XP_031027105.1 uncharacterized protein SmJEL517_g00807	2.00E-152
Rhizoclosmatium globosum JEL800	NF	NF	ORY38136.1 family A G protein-coupled receptor-like protein, ORY32468.1 family A G protein-coupled receptor-like protein,	2e-08, 8e-08	NF	NF
Chytriomyces confervae CBS 675.73	NF	NF	TPX66892.1 hypothetical protein CcCBS67573_g07690, TPX66891.1 hypothetical protein CcCBS67573_g07691	5e-13, 5e-11	NF	NF
Spizellomyces punctatus BR117	NF	NF	Spi_pu_XP_016612672.1_hypothetical_protein_SPPG_00350	0.00E+00	XP_016606298.1_hypothetical_protein,_variant_2, Spi_pu_XP_016606299.1_hypothetical_protein,_variant_1, Spi_pu_XP_016606300.1_hypothetical_protein_SPPG_06658	1.00E-40
Globomyces pollinis-pini Arg68	NF	NF	jgi|Glopol1|363053	6.00E-14	NF	NF
Batrachochytrium dendrobatidis JAM81	NF	NF	gb|EGF80153.1|_hypothetical_protein_BATDEDRAFT_88919	0.00E+00	NF	NF
Homolaphlyctis polyrhiza JEL142	NF	NF	AFSM01000868.1_Homolaphlyctis_polyrhiza_JEL_142_strain	0.00E+00	NF	NF
Amoeboradix gromovi X-113	NF	NF	NF	NF	NF	NF
Sanchytrium tribonematis X-128	NF	NF	NF	NF	NF	NF
Allomyces macrogynus ATCC 38327	NF	NF	KNE54739.1_hypothetical_protein_AMAG_00698, KNE56389.1_hypothetical_protein_AMAG_02198	0.00E+00	NF	NF
Blastocladiella emersonii ATCC 22665	NF	NF	gi|148884807|gb|EE735065.1|EE735065.p2	6.00E-27	NF	NF
Catenaria anguillulae PL171	NF	NF	ORZ32981.1_hypothetical_protein_BCR44DRAFT_90252	7.00E-07	NF	NF
Olpidium bornovanus S191	NF	NF	KAG5455987.1 hypothetical protein BJ554DRAFT_4394	1.00E-05	NF	NF
Basidiobolus meristosporus CBS 931.73	NF	NF	NF	NF	ORX89367.1 hypothetical protein K493DRAFT_305838	8.00E-54
Basidiobolus heterosporus B8920	NF	NF	NF	NF	NF	NF
Conidiobolus coronatus NRRL 28638	NF	NF	NF	NF	NF	NF

Entomophthora muscae	NF	NF	NF	NF	NF	NF
Coemansia reversa NRRL 1564	NF	NF	NF	NF	NF	NF
Capniomyces stellatus MIS-10-108	NF	NF	NF	NF	NF	NF
Glomus cerebriforme	NF	NF	NF	NF	NF	NF
Rhizophagus irregularis DAOM 197198	NF	NF	NF	NF	NF	NF
Mortierella antarctica	NF	NF	NF	NF	NF	NF
Mortiereinala alpina ATCC 32222	NF	NF	NF	NF	NF	NF
Mucor circinelloides JCM 22480	NF	NF	NF	NF	NF	NF
Umbelopsis isabellina B7317	NF	NF	NF	NF	KAG2181921.1_hypothetical_protein_INT43_006846__Umbelopsis_isabellina	NF3e-29
Rhizopus delemar RA 99-880	NF	NF	NF	NF	NF	NF
Rhizopus oryzae 99-892	NF	NF	NF	NF	NF	NF
Phycomyces blakesleeanus NRRL 1555	NF	NF	NF	NF	NF	NF
Cryptococcus neoformans var. grubii H99	XP_012051341.1 opsin 1 [Cryptococcus neoformans var. grubii H99]	3.00E-27	NF	NF	XP_012052525.1 bacteriophytochrome histidine kinase, XP_012052892.1 bacteriophytochrome histidine kinase, varian	0.00E+00
Ustilago maydis 521	XP_011386259.1 hypothetical protein UMAG_00371 [Ustilago maydi, XP_011389026.1 hypothetical protein UMAG_02629 [Ustilago maydi	1e-35, 3e-31	NF	NF	XP_011391477.1 hypothetical protein UMAG_05732	0.00E+00
Puccinia graminis f. sp. tritici CRL 75-36-700-3	XP_003330211.1 hypothetical protein PGTG_11121	3.00E-18	NF	NF	XP_003889564.1 hypothetical protein PGTG_21762	0.00E+00
Coprinopsis cinerea okayama7#130	NF	NF	NF	NF	XP_001835879.2 atypical/HisK protein kinase	0.00E+00
Aspergillus nidulans FGSC A4	XP_660965.1 hypothetical protein AN3361.2	2.00E-64	NF	NF	XP_682277.1 hypothetical protein AN9008.2	0.00E+00
Trichoderma atroviride IMI 206040	XP_013949009.1 hypothetical protein TRIATDRAFT_210598	1.00E-20	NF	NF	XP_013942313.1 hypothetical protein TRIATDRAFT_319399	0.00E+00
Neurospora crassa OR74A	AAD45253.1_opsin-1_[Neurospora_crassa]	0.00E+00	NF	NF	XP_960393.2 sensor histidine kinase/response regulator, XP_960050.2 phytochrome-like histidine kinase 2	0.00E+00
Fusarium oxysporum NRRL 32931	XP_031048691.1 uncharacterized protein FOYG_03723, XP_031030491.1 uncharacterized protein FOYG_15804	2E-118, 8e-31	NF	NF	XP_031037841.1 uncharacterized protein FOYG_09928	0.00E+00
Saitoella complicata NRRL Y-17804	XP_019021587.1 family A G protein-coupled receptor-like protein	5.00E-58	NF	NF	XP_019024539.1 hypothetical protein SAICODRAFT_114514	0.00E+00
Sclerotinia sclerotiorum 1980 UF-70	XP_001597420.1 opsin-1 [Sclerotinia sclerotiorum 1980 UF-70]	2.00E-93	NF	NF	XP_001585628.1 hypothetical protein SS1G_13512, XP_001596430.1 hypothetical protein SS1G_02650, XP_001586250.1 hypothetical protein SS1G_12828	0.00E+00
Saccharomyces cerevisiae S288C	NF	NF	NF	NF	NF	NF
Schizosaccharomyces pombe (ASM294v2)	NF	NF	NF	NF	NF	NF

Table S3. Presence and absence of CyclOp pathway elements (green), Carotenoid biosynthesis proteins (yellow) and photoreceptors (red) across 45 fungal proteomes, Related to Figure 3. NF = Not Found.

References

S1. Avelar, G.M., Schumacher, R.I., Zaini, P.A., Leonard, G., Richards, T.A., and Gomes, S.L. (2014). A Rhodopsin-Guanylyl cyclase gene fusion functions in visual perception in a fungus. Curr. Biol. *24*, 1234–1240.

S2. Torruella, G., Grau-Bové, X., Moreira, D., Karpov, S.A., Burns, J.A., Sebé-Pedrós, A., Völcker, E., and López-García, P. (2018). Global transcriptome analysis of the aphelid Paraphelidium tribonemae supports the phagotrophic origin of fungi. Commun. Biol. *1*, 1–11.

S3. Chang, Y., Wang, S., Sekimoto, S., Aerts, A.L., Choi, C., Clum, A., LaButti, K.M., Lindquist, E.A., Ngan, C.Y., Ohm, R.A., et al. (2015). Phylogenomic analyses indicate that early fungi evolved digesting cell walls of algal ancestors of land plants. Genome Biol. Evol. *7*, 1590–1601.

S4. Mondo, S.J., Dannebaum, R.O., Kuo, R.C., Louie, K.B., Bewick, A.J., LaButti, K., Haridas, S., Kuo, A., Salamov, A., Ahrendt, S.R., et al. (2017). Widespread adenine N6-methylation of active genes in fungi. Nat. Genet. *49*, 964–968.

S5. van de Vossenberg, B.T.L.H., Warris, S., Nguyen, H.D.T., van Gent-Pelzer, M.P.E., Joly, D.L., van de Geest, H.C., Bonants, P.J.M., Smith, D.S., Lévesque, C.A., and van der Lee, T.A.J. (2019). Comparative genomics of chytrid fungi reveal insights into the obligate biotrophic and pathogenic lifestyle of Synchytrium endobioticum. Sci. Rep. *9*, 8672.

S6. Russ, C., Lang, B.F., Chen, Z., Gujja, S., Shea, T., Zeng, Q., Young, S., Cuomo, C.A., and Nusbaum, C. (2016). Genome sequence of Spizellomyces punctatus. Genome Announc. *4*, e00849-16.

S7. Joneson, S., Stajich, J.E., Shiu, S.H., and Rosenblum, E.B. (2011). Genomic transition to pathogenicity in chytrid fungi. PLoS Pathog. *7*.

S8. Galindo, L.J., López-García, P., Torruella, G., Karpov, S., and Moreira, D. (2021). Phylogenomics of a new fungal phylum reveals multiple waves of reductive evolution across Holomycota. Nat. Commun. *12*, 4973.

S9. Ribichich, K.F., Salem-Izacc, S.M., Georg, R.C., Vêncio, R.Z.N., Navarro, L.D., and Gomes, S.L. (2005). Gene discovery and expression profile analysis through sequencing of expressed sequence tags from different developmental stages of the chytridiomycete Blastocladiella emersonii. Eukaryot. Cell *4*, 455–464.

S10. Chang, Y., Rochon, D., Sekimoto, S., Wang, Y., Chovatia, M., Sandor, L., Salamov, A., Grigoriev, I. V, Stajich, J.E., and Spatafora, J.W. (2021). Genome-scale phylogenetic analyses confirm Olpidium as the closest living zoosporic fungus to the non-flagellated, terrestrial fungi. Sci. Rep. *11*, 3217.

S11. Chibucos, M.C., Soliman, S., Gebremariam, T., Lee, H., Daugherty, S., Orvis, J., Shetty, A.C., Crabtree, J., Hazen, T.H., Etienne, K.A., et al. (2016). An integrated genomic and transcriptomic survey of mucormycosis-causing fungi. Nat. Commun. *7*, 12218.

S12. De Fine Licht, H.H., Jensen, A.B., and Eilenberg, J. (2017). Comparative transcriptomics reveal host-specific nucleotide variation in entomophthoralean fungi. Mol. Ecol. *26*, 2092–2110.

S13. Wang, Y., White, M.M., and Moncalvo, J.-M. (2016). Draft genome sequence of Capniomyces stellatus, the obligate gut fungal symbiont of stonefly. Genome Announc. *4*, e00761-16.

S14. Morin, E., Miyauchi, S., San Clemente, H., Chen, E.C.H., Pelin, A., de la Providencia, I., Ndikumana, S., Beaudet, D., Hainaut, M., Drula, E., et al. (2019). Comparative genomics of Rhizophagus irregularis, R. cerebriforme, R. diaphanus and Gigaspora rosea highlights specific genetic features in Glomeromycotina. New Phytol. *222*, 1584–1598.

S15. Chen, E.C.H., Morin, E., Beaudet, D., Noel, J., Yildirir, G., Ndikumana, S., Charron, P., St-Onge, C., Giorgi, J., Krüger, M., et al. (2018). High intraspecific genome diversity in the model arbuscular mycorrhizal symbiont Rhizophagus irregularis. New Phytol. *220*, 1161–1171.

S16. Vandepol, N., Liber, J., Desirò, A., Na, H., Kennedy, M., Barry, K., Grigoriev, I. V, Miller, A.N., O'Donnell, K., Stajich, J.E., et al. (2020). Resolving the Mortierellaceae phylogeny through synthesis

of multi-gene phylogenetics and phylogenomics. Fungal Divers. *104*, 267–289.

S17. Wang, L., Chen, W., Feng, Y., Ren, Y., Gu, Z., Chen, H., Wang, H., Thomas, M.J., Zhang, B., Berquin, I.M., et al. (2011). Genome characterization of the oleaginous fungus Mortierella alpina. PLoS One *6*, 1–16.

S18. Navarro-Mendoza, M.I., Pérez-Arques, C., Panchal, S., Nicolás, F.E., Mondo, S.J., Ganguly, P., Pangilinan, J., Grigoriev, I. V, Heitman, J., Sanyal, K., et al. (2019). Early diverging fungus Mucor circinelloides lacks centromeric histone CENP-A and displays a mosaic of point and regional centromeres. Curr. Biol. *29*, 3791-3802.e6.

S19. Ma, L.-J., Ibrahim, A.S., Skory, C., Grabherr, M.G., Burger, G., Butler, M., Elias, M., Idnurm, A., Lang, B.F., Sone, T., et al. (2009). Genomic analysis of the basal lineage fungus Rhizopus oryzae reveals a whole-genome duplication. PLOS Genet. *5*, 1–11.

S20. Corrochano, L.M., Kuo, A., Marcet-Houben, M., Polaino, S., Salamov, A., Villalobos-Escobedo, J.M., Grimwood, J., Álvarez, M.I., Avalos, J., Bauer, D., et al. (2016). Expansion of signal transduction pathways in fungi by extensive genome duplication. Curr. Biol. *26*, 1577–1584.

S21. Janbon, G., Ormerod, K.L., Paulet, D., Byrnes III, E.J., Yadav, V., Chatterjee, G., Mullapudi, N., Hon, C.-C., Billmyre, R.B., Brunel, F., et al. (2014). Analysis of the genome and transcriptome of Cryptococcus neoformans var. grubii reveals complex RNA expression and microevolution leading to virulence attenuation. PLOS Genet. *10*, 1–26.

S22. Kämper, J., Kahmann, R., Bölker, M., Ma, L.-J., Brefort, T., Saville, B.J., Banuett, F., Kronstad, J.W., Gold, S.E., Müller, O., et al. (2006). Insights from the genome of the biotrophic fungal plant pathogen Ustilago maydis. Nature *444*, 97–101.

S23. Duplessis, S., Cuomo, C.A., Lin, Y.-C., Aerts, A., Tisserant, E., Veneault-Fourrey, C., Joly, D.L., Hacquard, S., Amselem, J., Cantarel, B.L., et al. (2011). Obligate biotrophy features unraveled by the genomic analysis of rust fungi. Proc. Natl. Acad. Sci. *108*, 9166–9171.

S24. Stajich, J.E., Wilke, S.K., Ahrén, D., Au, C.H., Birren, B.W., Borodovsky, M., Burns, C., Canbäck, B., Casselton, L.A., Cheng, C.K., et al. (2010). Insights into evolution of multicellular fungi from the assembled chromosomes of the mushroom Coprinopsis cinerea (Coprinus cinereus). Proc. Natl. Acad. Sci. U. S. A. *107*, 11889–11894.

S25. Galagan, J.E., Calvo, S.E., Cuomo, C., Ma, L.-J., Wortman, J.R., Batzoglou, S., Lee, S.-I., Baştürkmen, M., Spevak, C.C., Clutterbuck, J., et al. (2005). Sequencing of Aspergillus nidulans and comparative analysis with A. fumigatus and A. oryzae. Nature *438*, 1105–1115.

S26. Kubicek, C.P., Herrera-Estrella, A., Seidl-Seiboth, V., Martinez, D.A., Druzhinina, I.S., Thon, M., Zeilinger, S., Casas-Flores, S., Horwitz, B.A., Mukherjee, P.K., et al. (2011). Comparative genome sequence analysis underscores mycoparasitism as the ancestral life style of Trichoderma. Genome Biol. *12*, R40.

S27. Galagan, J.E., Calvo, S.E., Borkovich, K.A., Selker, E.U., Read, N.D., Jaffe, D., FitzHugh, W., Ma, L.-J., Smirnov, S., Purcell, S., et al. (2003). The genome sequence of the filamentous fungus Neurospora crassa. Nature *422*, 859–868.

S28. DeIulio, G.A., Guo, L., Zhang, Y., Goldberg, J.M., Kistler, H.C., Ma, L.-J., and Mitchell, A.P. (2018). Genome expansion in the Fusarium oxysporum species complex driven by accessory chromosomes. mSphere *3*, e00231-18.

S29. Riley, R., Haridas, S., Wolfe, K.H., Lopes, M.R., Hittinger, C.T., Göker, M., Salamov, A.A., Wisecaver, J.H., Long, T.M., Calvey, C.H., et al. (2016). Comparative genomics of biotechnologically important yeasts. Proc. Natl. Acad. Sci. *113*, 9882–9887.

S30. Amselem, J., Cuomo, C.A., van Kan, J.A.L., Viaud, M., Benito, E.P., Couloux, A., Coutinho, P.M., de Vries, R.P., Dyer, P.S., Fillinger, S., et al. (2011). Genomic analysis of the necrotrophic fungal pathogens Sclerotinia sclerotiorum and Botrytis cinerea. PLoS Genet. *7*, e1002230.

S31. Goffeau, A., Barrell, G., Bussey, H., Davis, R.W., Dujon, B., Feldmann, H., Galibert, F., Hoheisel, J.D.,

Jacq, C., Johnston, M., et al. (1996). Life with 6000 genes. Science (80-.). *274*, 563–567.

S32. Wood, V., Gwilliam, R., Rajandream, M.-A., Lyne, M., Lyne, R., Stewart, A., Sgouros, J., Peat, N., Hayles, J., Baker, S., et al. (2002). The genome sequence of Schizosaccharomyces pombe. Nature *415*, 871–880.

S33. Reichle, R.E., and Fuller, M.S. (1967). The fine structure of Blastocladiella emersonii zoospores. Am. J. Bot. *54*, 81–92.

S34. Cantino, E.C., and Truesdell, L.C. (1970). Organization and fine structure of the side body and its lipid sac in the zoospore of Blastocladiella emersonii. Mycologia *62*, 548–567.

S35. Manier, J.-F. (1977). Cycle, ultrastructure d'une Catenaria (Phycomycètes, Blastocladiales) parasite de Crustacés Cyclopoides. Ann. Parasitol. Hum. Comp. *52*, 363–376.

S36. Fuller, M.S., and Olson, L.W. (1971). The zoospore of Allomyces. Microbiology *66*, 171–183.

S37. Karpov, S.A., Vishnyakov, A.E., Moreira, D., and López-García, P. (2019). The ultrastructure of Sanchytrium tribonematis (Sanchytriaceae, Fungi incertae sedis) confirms its close relationship to Amoeboradix. J. Eukaryot. Microbiol. *66*, 892–898.

S38. Karpov, S.A., López-García, P., Mamkaeva, M.A., Klimov, V.I., Vishnyakov, A.E., Tcvetkova, V.S., and Moreira, D. (2018). The chytrid-like parasites of algae Amoeboradix gromovi gen. et sp. nov. and Sanchytrium tribonematis belong to a new fungal lineage. Protist *169*, 122–140.

S39. Barr, D.J.S., and Hartmann, V.E. (1976). Zoospore ultrastructure of three Chytridium species and Rhizoclosmatium globosum. Can. J. Bot. *54*, 2000–2013.

S40. Letcher, P.M., Vélez, C.G., Barrantes, M.E., Powell, M.J., Churchill, P.F., and Wakefield, W.S. (2008). Ultrastructural and molecular analyses of Rhizophydiales (Chytridiomycota) isolates from North America and Argentina. Mycol. Res. *112*, 759–782.

S41. Longcore, J.E., Simmons, D.R., and Letcher, P.M. (2016). Synchytrium microbalum sp. nov. is a saprobic species in a lineage of parasites. Fungal Biol. *120*, 1156–1164.

S42. Mollicone, M.R.N., and Longcore, J.E. (1999). Zoospore Ultrastructure of Gonapodya polymorpha. Mycologia *91*, 727–734.
